# A robust test for X-chromosome genetic association accounting for X-chromosome inactivation and imprinting

**DOI:** 10.1017/S0016672320000026

**Published:** 2020-04-01

**Authors:** Yu Zhang, Si-Qi Xu, Wei Liu, Wing Kam Fung, Ji-Yuan Zhou

**Affiliations:** 1 State Key Laboratory of Organ Failure Research, Ministry of Education, and Guangdong Provincial Key Laboratory of Tropical Disease Research, Department of Biostatistics, School of Public Health, Southern Medical University, Guangzhou, China; 2 Department of Statistics and Actuarial Science, The University of Hong Kong, Hong Kong, China

**Keywords:** association test, imprinting effects, inactivation, X chromosome

## Abstract

The X chromosome is known to play an important role in many sex-specific diseases. However, only a few single-nucleotide polymorphisms on the X chromosome have been found to be associated with diseases. Compared to the autosomes, conducting association tests on the X chromosome is more intractable due to the difference in the number of X chromosomes between females and males. On the other hand, X-chromosome inactivation takes place in female mammals, which is a phenomenon in which the expression of one copy of two X chromosomes in females is silenced in order to achieve the same gene expression level as that in males. In addition, imprinting effects may be related to certain diseases. Currently, there are some existing approaches taking X-chromosome inactivation into account when testing for associations on the X chromosome. However, none of them allows for imprinting effects. Therefore, in this paper, we propose a robust test, *Z*_XCII_, which accounts for both X-chromosome inactivation and imprinting effects without requiring specifying the genetic models in advance. Simulation studies are conducted in order to investigate the validity and performance of *Z*_XCII_ under various scenarios of different parameter values. The simulation results show that *Z*_XCII_ controls the type I error rate well when there is no association. Furthermore, with regards to power, *Z*_XCII_ is robust in all of the situations considered and generally outperforms most of the existing methods in the presence of imprinting effects, especially under complete imprinting effects.

## 1. Introduction

The X chromosome has been found to play an important role in many complex diseases (Ober *et al.*, 2008; Wise *et al.*, 2013). However, the development of methods for detecting associations with X-linked markers has lagged behind that for autosomal markers due to the complexity of the inheritance patterns of the X chromosome (Wise *et al.*, 2013; Schurz *et al.*, 2019). One primary characteristic of the X chromosome in mammals is that females have two copies of the X chromosome while males only have one, which increases the difficulty of X-linked association studies (Clayton, 2009; Ziegler, 2009; Loley *et al.*, 2011). In addition, the phenomenon of X-chromosome inactivation (XCI) in females may constitute a risk factor for diseases, which is defined as the expression silencing of one of the two copies of the X chromosome in females. Thus, the X-chromosome gene dosage in female XX cells equals that in male XY cells, namely dosage compensation (Chow *et al.*, 2005; Payer & Lee, 2008; Pessia *et al.*, 2012). As such, the genetic effect of homozygous females can be regarded as the same as that of hemizygous males under XCI. It has been reported that most of the genes on the X chromosome are subject to XCI, while only about 15% of X-linked genes escape from inactivation (XCI-E) (Carrel & Willard, 2005). Random X-chromosome inactivation (XCI-R) is the general process of XCI by which one of the two copies of the X chromosome in each cell is randomly inactivated. But the XCI patterns in some females may become skewed from that of the XCI-R in an age- and tissue-dependent manner, and the same allele can be inactivated in more than 75% of cells in some cases (Migeon, 1998; Minks *et al.*, 2008; Starmer & Magnuson, 2009; Wang *et al.*, 2014), which is denoted by XCI-S for convenience.

At present, there are some association tests available for single-nucleotide polymorphisms (SNPs) on the X chromosome. Zheng *et al.* (2007) proposed six methods for testing associations on the X chromosome by combining the genetic effects in females and males. Among them, the allele-based tests *Z*_*A*_ and *Z*_*mfA*_ require the assumption of a Hardy–Weinberg equilibrium (HWE), while the genotype-based methods *Z*_*C*_ and *Z*_*mfG*_ are robust to departures from a HWE. Furthermore, note that all four methods mentioned above rely on the assumption that females and males have the same risk alleles. Thus, two other methods (*Z*˜_*mfA*_ and *Z*˜_*mfG*_) were developed and are applicable to the situation in which females and males have different risk alleles. On the other hand, the six methods of Zheng *et al.* (2007) only consider the information on XCI-E and do not take account of XCI, which may lead to loss of power if XCI is present. Clayton (2008) was the first to suggest that XCI should be considered in X-chromosome association studies. Clayton's methods (*T*_*A*_ and *T*_*AD*_) are equivalent to the score tests of generalized linear models accounting for XCI-R and give the same codes for homozygous females and hemizygous males. When the allele frequencies of the same allele differ between the sexes, the test statistics 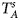, 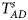 and *S*_*A*_, stratified by sex, have been proposed by Loley *et al.* (2011) and König *et al.* (2014). In addition, a software toolset *XWAS* (Gao *et al.*, 2015) includes four tests (*FM*_01_, *FM*_02_, *FM*_*F*_ and *FM*_*S*_) based on logistic regressions. However, those approaches only consider XCI-R and ignore XCI-S. In order to simultaneously incorporate three biological patterns on the X chromosome (XCI-E, XCI-R and XCI-S), Wang *et al.* (2014) developed a maximum likelihood ratio method. However, this method is time-consuming because it is a permutation-based procedure for obtaining an empirical *P*-value. Meanwhile, Chen *et al.* (2017) proposed a robust method (*Xcat*) based on a generalized genetic model with the approximate *P*-value being easily obtained. Recently, Wang *et al.* (2019) proposed a robust test, *Z*_*max*_, by taking account of different dosage compensation patterns, which requires neither the assumption of a HWE nor the specification of underlying genetic models.

Imprinting is an epigenetic phenomenon that results in the differential expression of paternal and maternal alleles (Falls *et al.*, 1999). Researchers have found evidence for the existence of imprinting effects on some diseases, such as Angelman, Beckwith–Wiedemann and Prader–Willi syndromes (Falls *et al.*, 1999; Dong *et al.*, 2005; Ziegler & König, 2006; Wallace *et al.*, 2010). On the other hand, it is likely that imprinted genes on the X chromosome are crucial to some diseases, such as Turner's syndrome (Donnelly *et al.*, 2002; Loesch *et al.*, 2005). For some sex-specific diseases, such as autism, alleles on the paternal chromosome seem to be preferentially expressed, which is likely to explain why females are always less susceptible than males (Skuse, 2000). Imprinting is generally detected through testing for parent-of-origin effects (Hager *et al.*, 2008). Thus, we use the term ‘parent-of-origin effects’ instead of ‘imprinting effects’ in the following sections. However, there is no method available for taking parent-of-origin effects into account when conducting association tests on the X chromosome.

Therefore, in this paper, we propose a robust method, *Z*_XCII_, which is an extension of *Xcat* to the generalized linear model simultaneously accounting for imprinting and three biological patterns (XCI-E, XCI-R and XCI-S) into X-chromosome association tests without the need to specify the genetic models on the X chromosome. We investigate the performance of the proposed method and compare it with several existing tests through extensive simulation studies. Simulation results show that the proposed method controls the size well under all of the scenarios considered when there is no association. Moreover, with regards to power, *Z*_XCII_ is robust in all of the situations considered and generally outperforms most of the existing methods in the presence of imprinting effects, especially under complete imprinting effects.

## 2. Materials and methods

For a candidate SNP on the X chromosome with the mutant allele *A* and the normal allele *a*, there are four ordered genotypes for female offspring: *a*/*a*, *a*/*A*, *A*/*a* and *A*/*A*, where the left (right) allele of the slash is paternal (maternal). To distinguish the parent of origin of the mutant allele *A* in heterozygous female offspring, the information on their parental genotypes is required. With regards to male offspring, there are only two kinds of genotypes, *a* and *A*, which are maternal. Thus, we do not need to collect their parental genotypes. Assume that *G*_*f*1_ and *G*_*f*2_ are the numbers of allele *A* on the paternal and maternal X chromosomes in female offspring, respectively, and *G*_*m*_ is the number of allele *A* on the X chromosome in male offspring. The values of *G*_*f*1_, *G*_*f*2_ and *G*_*m*_ for different genotypes in the offspring generation are shown in Table 1. The disease status of an individual (female or male) in the offspring generation is denoted by *Y* with 1 (0) representing being affected (unaffected). In this paper, an affected daughter together with her parents is called a case–parent trio and an unaffected daughter together with her parents is considered as a control–parent trio (Deng & Chen, 2001; Li *et al.*, 2016). Table 2 gives the genotype counts for the female offspring, where *n*_*f*_ is the total number of daughter–parent trios consisting of *r*_*f*_ case–parent trios and *s*_*f*_ control–parent trios. The genotype counts for the male offspring are also listed in Table 2, where *n*_*m*_ is the total number of males including *r*_*m*_ cases and *s*_*m*_ controls. As such, there are *n*_*r*_ = *r*_*f*_ + *r*_*m*_ cases and *n*_*s*_ = *s*_*f*_ + *s*_*m*_ controls in total. Therefore, the sample size is *N* = *n*_*r*_ + *n*_*s*_ = *n*_*f*_ + *n*_*m*_. Let *ϕ*_*f*0_, *ϕ*_*f*01_, *ϕ*_*f*10_ and *ϕ*_*f*2_ be the penetrances of genotypes *a*/*a*, *a*/*A*, *A*/*a* and *A*/*A* in female offspring, respectively, and let *ϕ*_*m*0_ and *ϕ*_*m*1_ be the penetrances of genotypes *a* and *A* in male offspring, respectively. To test the association between the disease status *Y* and the SNP under study, we make the following two assumptions, just like *Xcat* (Chen *et al.*, 2017): (1) in the presence of association between the disease and the SNP, the generalized genetic model is assumed to hold in female offspring with ordered penetrances, either increasing (*ϕ*_*f*0_ ⩽ *ϕ*_*f*01_, *ϕ*_*f*10_ ⩽ *ϕ*_*f*2_) or decreasing (*ϕ*_*f*0_ ⩾ *ϕ*_*f*01_, *ϕ*_*f*10_ ⩾ *ϕ*_*f*2_); and (2) the mutant allele in female offspring is the same as that in male offspring.

**Table 1.** Values of *G*_*f*1_, *G*_*f*2_ and *G*_*m*_ for different genotypes in the offspring generation.

Female				Male	
Genotype	*G*_*f*1_	*G*_*f*2_	*Logit*(*Pr*(*Y* = 1|*G*_*f*1_, *G*_*f*2_, ***X***_*f*_))	Genotype	*G*_*m*_
*a*/*a*	0	0	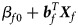	*a*	0
*a*/*A*	0	1	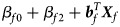	*A*	1
*A*/*a*	1	0	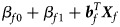		
*A*/*A*	1	1	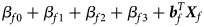

**Table 2.** Genotype counts for the single-nucleotide polymorphism on the X chromosome stratified by sex in the offspring generation.

	Female					Male		
Group	*a*/*a*	*a*/*A*	*A*/*a*	*A*/*A*	Total	*a*	*A*	Total
Case	*r_a_*_/*a*_	*r_a_*_/*A*_	*r_A_*_/*a*_	*r_A_*_/*A*_	*r*_*f*_	*r*_*a*_	*r*_*A*_	*r*_*m*_
Control	*s_a_*_/*a*_	*s_a_*_/*A*_	*s_A_*_/*a*_	*s_A_*_/*A*_	*s*_*f*_	*s*_*a*_	*s*_*A*_	*s*_*m*_
Total	*n_a_*_/*a*_	*n_a_*_/*A*_	*n_A_*_/*a*_	*n_A_*_/*A*_	*n*_*f*_	*n*_*a*_	*n*_*A*_	*n*_*m*_

A logistic regression model is proposed to describe the association between the disease and the SNP in female offspring:1
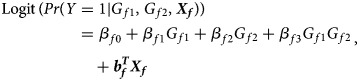
where *β*_*f*0_ is the intercept, *β*_*f*1_, *β*_*f*2_ and *β*_*f*3_ are the respective regression coefficients for *G*_*f*1_, *G*_*f*2_ and the interaction term *G*_*f*1_*G*_*f*2_, ***X****_f_* is a vector of covariates and ***b***_*f*_ is a vector of the regression coefficients for ***X****_f_*. The estimates of these coefficients can be obtained with the iteratively reweighted least squares method (Wood, 2006) using the *glm* function in *R* language (http://www.r-project.org). The null hypothesis of no association between the disease and the SNP in female offspring is *H*_*f*0_∶*β*_*f*1_ = *β*_*f*2_ = *β*_*f*3_ = 0. If at least one of these equations is not satisfied, then the association exists, which indicates the alternative hypothesis (*H*_*f*1_). Logit(*Pr*(*Y* = 1|*G*_*f*1_, *G*_*f*2_, ***X***_*f*_)) outcomes for different genotypes in female offspring are presented in the fourth column of Table 1. Thus, under *H*_*f*1_, the parent-of-origin effects at the SNP locus can be expressed by:2
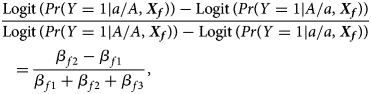
when ***X****_f_* is fixed at the same level. For example, *β*_*f*1_ = *β*_*f*2_ represents no parent-of-origin effects, while *β*_*f*2_ = *β*_*f*3_ = 0 denotes complete maternal parent-of-origin effect and *β*_*f*1_ = *β*_*f*3_ = 0 indicates complete paternal parent-of-origin effect. Moreover, we can use3
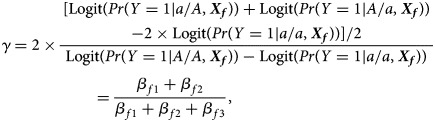
to measure the degree of inactivation under XCI in a similar way to Wang *et al.* (2019). On the other hand, the difference between *β*_*f*3_ and 0 can be interpreted as the deviation of the genetic model from the additive one under XCI-E. To be specific, Table 3 gives the explanations of the regression coefficients for several situations of XCI and XCI-E under no parent-of-origin effects (*β*_*f*1_ = *β*_*f*2_ = *β*). *β*_*f*1_ = *β*_*f*2_ = −*β*_*f*3_ means XCI-S with *γ* = 2 representing 100% of the cells having the mutant allele active or a dominant model under XCI-E. *β*_*f*1_ = *β*_*f*2_ = *β* and 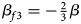 stand for XCI-S with *γ* = 1.5, where 75% of the cells have the mutant allele active. *β*_*f*1_ = *β*_*f*2_ ≠ 0 and *β*_*f*3_ = 0 correspond to XCI-R with *γ* = 1 or an additive model under XCI-E. *β*_*f*1_ = *β*_*f*2_ = *β* and *β*_*f*3_ = 2*β* imply XCI-S with *γ* = 0.5, where 25% of the cells have the mutant allele active. *β*_*f*1_ = *β*_*f*2_ = 0 and *β*_*f*3_ ≠ 0 indicate XCI-S with *γ* = 0 representing that 100% of the cells have the normal allele active or a recessive model under XCI-E. However, in the presence of parent-of-origin effects, the explanation of the regression coefficients is more complicated, since parent-of-origin effects may contribute to the XCI. For example, *β*_*f*1_ = 0.5 and *β*_*f*2_ = *β*_*f*3_ = 0 are indicative of the complete maternal parent-of-origin effect, whereas *γ* is obtained to be 1 (suggesting XCI-R) in this case. Therefore, XCI-R may be also caused by the complete maternal parent-of-origin effect.

**Table 3.** Explanation of the regression coefficients under no parent-of-origin effects.

Coefficients	*γ*	XCI	XCI-E
*β*_*f*1_ = *β*_*f*2_ = −*β*_*f*3_	2	XCI-S (100% of the cells have the mutant allele active)	Dominant model
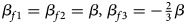	1.5	XCI-S (75% of the cells have the mutant allele active)	—
*β*_*f*1_ = *β*_*f*2_ ≠ 0, *β*_*f*3_ = 0	1	XCI-R (random XCI)	Additive model
*β*_*f*1_ = *β*_*f*2_ = *β*, *β*_*f*3_ = 2*β*	0.5	XCI-S (25% of the cells have the mutant allele active)	—
*β*_*f*1_ = *β*_*f*2_ = 0, *β*_*f*3_ ≠ 0	0	XCI-S (100% of the cells have the normal allele active)	Recessive model

XCI = X-chromosome inactivation.

Recall that when the disease is associated with the SNP, the generalized genetic model with ordered penetrances is assumed to hold in female offspring. As such, we have
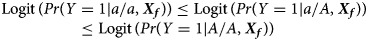
and
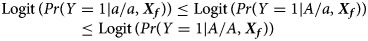
which are equivalent to 0 ⩽ *β*_*f*1_ ⩽ *β*_*f*1_ + *β*_*f*2_ + *β*_*f*3_ and 0 ⩽ *β*_*f*2_ ⩽ *β*_*f*1_ + *β*_*f*2_ + *β*_*f*3_, respectively, with at least one inequality being strict. Adding these two inequalities together, we get 0 ⩽ *β*_*f*1_ + *β*_*f*2_ ⩽ 2(*β*_*f*1_ + *β*_*f*2_ + *β*_*f*3_) and thus *β*_*f*1_ + *β*_*f*2_ + 2*β*_*f*3_ ⩾ 0. Therefore, the alternative hypothesis becomes *H*_*f*1_∶*β*_*f*1_ ⩾ 0, *β*_*f*2_ ⩾ 0, *β*_*f*1_ + *β*_*f*2_ + 2*β*_*f*3_ ⩾ 0, with at least one inequality being strict, which can be expressed in matrix form as follows:4
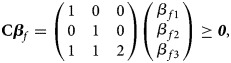
where 
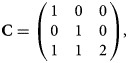
 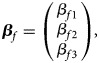 and ***0*** is a vector with all of the elements being 0. To test for the association, we first consider the following test statistics:5
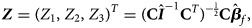
where 
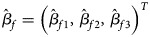
 with 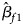, 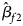 and 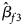 being the maximum likelihood estimates of *β*_*f*1_, *β*_*f*2_ and *β*_*f*3_, respectively. 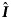 is the empirical Fisher's information matrix (Wood, 2006).

Under the null hypothesis of no association, *Z*_1_, *Z*_2_ and *Z*_3_ are independent of one another and asymptotically have standard normal distributions. Note that 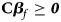 leads to ***Z*** ⩾ ***0*** under *H*_*f*1_, and we thus only calculate the right-sided *P*-values for *Z*_1_, *Z*_2_ and *Z*_3_, respectively. Then, we combine them using the Fisher's method (Fisher, 1954). Thus, the test statistic for female offspring can be constructed as:6
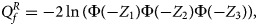
where Φ(⋅) is the cumulative distribution function of the standard normal distribution. Under the null hypothesis, 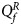 has an asymptotic *χ*^2^ distribution with degrees of freedom (*df*) being 6. As such, the *P*-value of 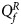 is 
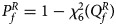
, where 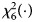 is the cumulative distribution function of the *χ*^2^ distribution with *df* being 6.

For male offspring, we model the relationship between the disease and the SNP using a logistic regression as:7
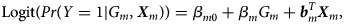
where *β*_*m*0_ is the intercept, *β*_*m*_ is the regression coefficient for *G_m_*, ***X****_m_* is a vector of covariates and ***b***_*m*_ is a vector of the regression coefficients for ***X****_m_*. When there is no association between the disease and the SNP, the null hypothesis for male offspring is *H*_*m*0_:*β*_*m*_ = 0. Then, the test statistic for male offspring is8
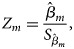
where 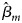 is the maximum likelihood estimate of *β*_*m*_ and 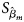 is the standard error of 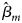. *Z*_*m*_ follows a standard normal distribution under *H*_*m*0_. When there are no covariates, Eq. (8) can be simplified to9
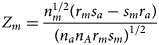
as in Zheng *et al.* (2007) and Chen *et al.* (2017).

For combining the test statistics of female and male offspring, we need to turn the *P*-value for female offspring (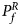) into a *Z*-score, which is 
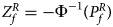
. Then, under the assumption that the mutant allele in female offspring is the same as that in male offspring, the combined test statistics *Z*^*R*^ can be constructed as follows:10
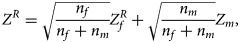
where 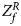 and *Z*_*m*_ are weighted by their respective proportions of the sample size. Under the overall null hypothesis that there is no association between the disease and the SNP in both female and male offspring (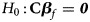 and *β*_*m*_ = 0), *Z*^*R*^ is asymptotically distributed as *N*(0, 1). Since the mutant allele is assumed to be *A*, with the overall one-sided alternative hypothesis 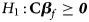 (with at least one inequality being strict) or *β*_*m*_ > 0, we only need to calculate the right-sided *P*-value of *Z*^*R*^ when the mutant allele is known in advance.

So far, we have only considered the situation when the mutant allele is *A*. When the mutant allele is *a*, the overall alternative hypothesis turns to be 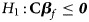 (with at least one inequality being strict) or *β*_*m*_ < 0. Therefore, the corresponding test statistic for female offspring is 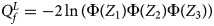, which combines the left-sided *P*-values of *Z*_1_, *Z*_2_ and *Z*_3_, and the *P*-value of 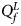 is 
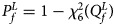
. Again, we combine the transformed *Z*-score (
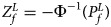
) for female offspring and *Z*_*m*_ for male offspring to obtain the overall test statistic as:11
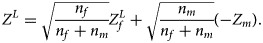
*Z*^*L*^ is asymptotically distributed as *N*(0, 1) under the overall null hypothesis. With this *H*_1_, just like *Z*^*R*^, only the right-sided *P*-value of *Z*^*L*^ is needed when the mutant allele is known to be *a* in advance.

However, we generally have no information on the mutant allele before conducting the association studies. In this case, we propose the test statistic as:12
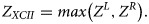
Although *Z*^*L*^ and *Z*^*R*^ are obviously dependent on each other, note that the components of ***Z***_*t*_ = (*Z*_1_, *Z*_2_, *Z*_3_, *Z*_*m*_)^*T*^ are independent of each other, and the functions −*Z*^*L*^ and *Z*^*R*^ of ***Z***_*t*_ are non-decreasing functions. Thus, the *P*-value of *Z*_*XCII*_ can be approximately bounded by13
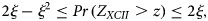
where *ξ* = 1 − Φ(*z*) according to Owen (2009) and Esary *et al.* (1967). Therefore, we can simply get the approximated *P*-value of *Z*_*XCII*_ by 2*ξ*.

## 3. Simulation study

### 3.1. Settings

We conduct a simulation study to investigate the size and power of the proposed *Z*_XCII_ method and compare it with the existing ones. Notice that in Zheng *et al.* (2007), and 
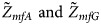
 are less powerful than the other four test statistics (*Z*_*A*_, *Z*_*C*_, *Z*_*mfA*_ and *Z*_*mfG*_) under the assumption that the mutant allele in females is the same as that in males. Thus, in this simulation study, *Z*˜_*mfA*_ and *Z*˜_*mfG*_ are excluded. 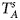 and *FM*_*S*_ are also excluded because they are asymptotically equivalent to *Z*_*C*_ (Loley *et al.*, 2011) and *Z*_*mfG*_ (Zheng *et al.*, 2007; Gao *et al.*, 2015; Wang *et al.*, 2019), respectively. On the other hand, the permutation-based method in Wang *et al.* (2014) is excluded due to the intensive computations involved. Finally, we choose 14 methods (*Z*_XCII_, *Z*_*max*_, *Xcat*, *S*_*A*_, *FM*_02_, *Z*_*C*_, *Z*_*mfG*_, *T*_*A*_, *T*_*AD*_, 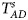, *FM*_01_, *FM*_*F*_, *Z*_*mfA*_ and *Z*_*A*_) for the comparison. The references for the selected methods are listed in Table S1.

Note that most of the methods we compare do not consider the covariates, such as *Xcat*, *S*_*A*_, *Z*_*C*_, *Z*_*mfG*_, *T*_*A*_, *T*_*AD*_, *Z*_*mfA*_ and *Z*_*A*_. Thus, we do not include any covariate for simplicity in this simulation study and directly generate the genotype counts in Table 2. Let 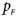 and 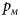 denote the frequencies of the mutant allele *A* for females and males in the parental generation, respectively. Under random mating, the genotype frequencies of *a*/*a*, *a*/*A*, *A*/*a* and *A*/*A* for female offspring are 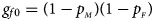, 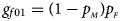, 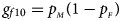 and 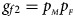, respectively, and the genotype frequencies of *a* and *A* for male offspring are 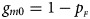 and 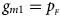, respectively. Note that if random mating holds in the parental generation, HWE holds in the offspring generation only under the assumption that the frequency of the same allele in females and that of males are equal (Puig *et al.*, 2017). On the other hand, we consider the situation where 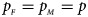 but HWE does not hold in the female offspring. The corresponding frequencies of the four genotypes are *g*_*f*0_ = (1 − *p*)^2^ + *ρp*(1 − *p*), *g*_*f*01_ = (1 − *ρ*)*p*(1 − *p*), *g*_*f*10_ = (1 − *ρ*)*p*(1 − *p*) and *g*_*f*2_ = *p*^2^ + *ρp*(1 − *p*), respectively, when the inbreeding coefficient *ρ* ≠ 0. Furthermore, the genotype frequencies for male offspring are *g*_*m*0_ = 1 − *p* and *g*_*m*1_ = *p*, respectively.

Note that the relationships among the penetrances and the regression coefficients are 
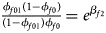
, 
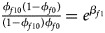
 and 
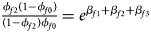
 for *a*/*A*, *A*/*a* and *A*/*A*, respectively, for female offspring and 
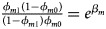
 for male offspring. Thus, genotype counts for female offspring in Table 2 can be generated according to a quadrinomial distribution with probabilities 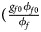, 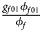, 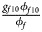, 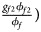 for cases and 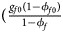, 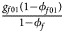, 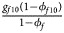, 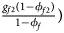 for controls, where *ϕ*_*f*_ = *g*_*f*0_*ϕ*_*f*0_ + *g*_*f*01_*ϕ*_*f*01_ + *g*_*f*10_*ϕ*_*f*10_ + *g*_*f*2_*ϕ*_*f*2_ is the disease prevalence of females. Similarly, we can obtain genotype counts for male offspring through a binomial distribution with probabilities 
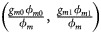
 for cases and 
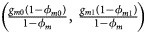
 for controls, where *ϕ*_*m*_ = *g*_*m*0_*ϕ*_*m*0_ + *g*_*m*1_*ϕ*_*m*1_ is the disease prevalence of males.

We consider various simulation settings. 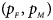 is taken to be (0.15, 0.25), (0.20, 0.20), (0.25, 0.15), (0.25, 0.35), (0.30, 0.30) and (0.35, 0.25). Then, under random mating, the corresponding allele frequencies for females and males in the offspring generation are (0.20, 0.15), (0.20, 0.20), (0.20, 0.25), (0.30, 0.25), (0.30, 0.30) and (0.30, 0.35), respectively. When 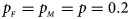 and 0.3, we set *ρ* = −0.05 and *ρ* = 0.05 for simulating the departure from HWE. *ϕ*_*f*0_ and *ϕ*_*m*0_ are set to be 0.120. For simulating the size, let all of the other penetrances be 0.120. When XCI exists, we suppose *ϕ*_*f*2_ = *ϕ*_*m*1_ = 0.240. The values of *γ* under XCI with different values of *ϕ*_*f*01_ and *ϕ*_*f*10_ are shown in Table S2. To investigate the power, we first consider the situations where there are both XCI and parent-of-origin effects: (1) (*ϕ*_*f*01_, *ϕ*_*f*10_) = (0.120, 0.240) (XCI with *γ* = 1 and complete maternal parent-of-origin effect); (2) (*ϕ*_*f*01_, *ϕ*_*f*10_) = (0.192, 0.216) (XCI with *γ* = 1.499 and incomplete maternal parent-of-origin effect); (3) (*ϕ*_*f*01_, *ϕ*_*f*10_) = (0.144, 0.204) (XCI with *γ* = 1.001 and incomplete maternal parent-of-origin effect); (4) (*ϕ*_*f*01_, *ϕ*_*f*10_) = (0.132, 0.156) (XCI with *γ* = 0.492 and incomplete maternal parent-of-origin effect); (5) (*ϕ*_*f*01_, *ϕ*_*f*10_) = (0.240, 0.120) (XCI with *γ* = 1 and complete paternal parent-of-origin effect); (6) (*ϕ*_*f*01_, *ϕ*_*f*10_) = (0.216, 0.192) (XCI with *γ* = 1.499 and incomplete paternal parent-of-origin effect); (7) (*ϕ*_*f*01_, *ϕ*_*f*10_) = (0.204, 0.144) (XCI with *γ* = 1.001 and incomplete paternal parent-of-origin effect); and (8) (*ϕ*_*f*01_, *ϕ*_*f*10_) = (0.156, 0.132) (XCI with *γ* = 0.492 and incomplete paternal parent-of-origin effect). Next, we take account of the scenarios where XCI exists but there are no parent-of-origin effects with *ϕ*_*f*01_ = *ϕ*_*f*10_ = *ϕ*: (1) *ϕ* = 0.240 (XCI with *γ* = 2); (2) *ϕ* = 0.204 (XCI with *γ* = 1.503); (3) *ϕ* = 0.168 (XCI with *γ* = 0.935); (4) *ϕ* = 0.144 (XCI with *γ* = 0.500); and (5) *ϕ* = 0.120 (XCI with *γ* = 0). Furthermore, we consider the situation where there is neither XCI nor parent-of-origin effects, which is (*ϕ*_*f*01_, *ϕ*_*f*10_, *ϕ*_*f*2_, *ϕ*_*m*1_) = (0.180, 0.180, 0.240, 0.180). The sample size *N* for each replication is selected to be 1000, including *n*_*r*_ = 500 cases and *n*_*s*_ = 500 controls. To investigate the effect of sex ratio, we fix the sex ratio in the control group as *s*_*f*_ : *s*_*m*_ = 1:1, while it varies in the case group as *r*_*f*_ : *r*_*m*_ = 3:2, 1:1 and 2:3. We use the significance level *α* = 10^−5^, and the number of replications is fixed to be 10^6^ and 10^4^ for estimating the size and power, respectively. The definitions of these parameters and the detailed biological meanings of the situations we consider are provided in Tables S3 and S4, respectively.

### 3.2. Size

Table 4 gives the estimated sizes of *Z*_XCII_, *Z*_max_, *Xcat*, *S*_*A*_, *FM*_02_, *Z*_*C*_, *Z*_*mfG*_, *T*_*A*_, *T*_*AD*_, 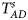, *FM*_01_, *FM*_*F*_, *Z*_*mfA*_ and *Z*_*A*_ under different simulation settings when random mating holds in the parental generation. From Table 4, we can see that *Z*_*XCII*_, *Z*_*max*_, *Xcat*, *FM*_02_, *Z*_*C*_, 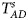, *FM*_01_, *FM*_*F*_, *Z*_*mfA*_ and *Z*_*A*_ generally control the size well, except that some of them produce a slightly conservative size under some situations. The sizes of *S*_*A*_ and *Z*_*mfG*_ are inflated when 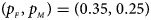 and the sex ratio is 3 : 2, and they stay close to the nominal level 10^−5^ for all of the other situations. *T*_*A*_ and *T*_*AD*_ can have inflated size when 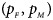 is equal to (0.25, 0.15) and (0.35, 0.25), which may be caused by the different allele frequencies between females and males in the offspring generation. However, they have a well-controlled size under the other situations. Table S5 reports the estimated sizes of different methods when 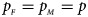 but HWE does not hold in female offspring. In addition, *Z*_*XCII*_, *Z*_*max*_, *Xcat*, *S*_*A*_, *FM*_02_, *Z*_*C*_, *Z*_*mfG*_, *T*_*A*_, *T*_*AD*_, 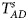, *FM*_01_ and *FM*_*F*_ generally control the size well. *Z*_*mfA*_ and *Z*_*A*_ can have inflated size when *ρ* = 0.05 and *p* = 0.30 since the allele-based test relies on the assumption of HWE in females.

**Table 4.** Estimated size (× 10^−5^) under random mating at significance level *α* = 10^−5^ based on 10^6^ replicates.^a^

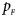	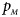	Sex ratio	*Z*_*XCII*_	*Z*_*max*_	*Xcat*	*S*_*A*_	*FM*_02_	*Z*_*C*_	*Z*_*mfG*_	*T*_*A*_	*T*_*AD*_	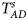	*FM*_01_	*FM*_*F*_	*Z*_*mfA*_	*Z*_*A*_
0.15	0.25	3:2	**0**.**3**	0.7	0.8	1.0	0.7	1.2	0.8	0.4	0.7	1.0	0.6	0.5	1.0	0.9
		1:1	1.0	1.0	1.1	1.1	0.6	1.0	1.4	0.4	0.9	0.9	1.1	0.6	1.0	1.1
		2:3	**0**.**2**	0.8	0.4	0.6	0.5	0.9	0.4	0.4	0.6	0.5	0.6	0.4	0.5	0.8
0.20	0.20	3:2	0.4	0.8	0.9	1.3	0.5	1.1	1.2	1.0	1.2	1.1	0.9	0.5	1.1	0.8
		1:1	0.7	0.8	0.6	0.8	0.8	0.5	0.9	0.8	1.2	0.7	0.5	0.4	0.8	0.8
		2:3	0.5	0.9	**0**.**3**	0.8	1.0	0.8	1.0	1.2	0.9	0.7	0.8	0.5	0.9	0.9
0.25	0.15	3:2	**0**.**3**	0.7	**0**.**2**	0.8	0.9	0.7	0.8	**2**.**9**	1.5	0.6	0.6	0.4	0.5	1.2
		1:1	**0**.**2**	0.5	0.7	1.1	**0**.**3**	0.8	0.9	1.5	0.7	0.6	0.7	0.8	0.5	0.6
		2:3	0.7	0.9	0.9	0.8	0.9	1.1	1.1	**2**.**5**	**2**.**1**	0.6	0.8	0.6	0.9	1.2
0.25	0.35	3:2	1.4	1.0	1.1	1.1	0.9	1.2	1.1	0.9	0.9	1.3	0.9	1.1	1.1	1.6
		1:1	0.5	1.0	1.0	0.8	0.8	0.7	0.9	0.6	0.7	0.9	0.9	0.7	1.4	1.3
		2:3	0.7	0.7	0.7	0.4	1.0	1.1	0.9	1.2	0.9	0.6	0.4	0.6	0.6	1.2
0.30	0.30	3:2	1.0	1.0	0.8	1.0	1.2	1.0	1.3	1.0	0.5	0.7	0.8	0.4	0.9	0.7
		1:1	0.6	0.7	**0**.**3**	0.6	0.7	0.4	1.0	1.1	**0**.**2**	0.6	0.7	**0**.**2**	0.8	0.9
		2:3	0.7	0.9	0.8	1.2	0.6	0.7	0.8	0.6	0.9	**0**.**2**	1.0	**0**.**3**	1.1	1.0
0.35	0.25	3:2	1.2	1.6	1.2	**1**.**7**	1.6	0.9	**1**.**7**	**2**.**1**	**1**.**7**	0.4	1.1	0.8	1.1	1.3
		1:1	1.3	0.9	0.7	1.4	1.2	0.9	1.6	**2**.**1**	0.8	0.4	1.2	0.7	1.2	1.2
		2:3	0.4	0.8	0.5	0.5	1.4	0.7	0.7	**1**.**7**	1.1	0.4	0.6	0.5	0.6	1.1

a Numbers that are outside of the 95% confidence interval (0.38 × 10^−5^, 1.62 × 10^−5^) are highlighted in bold.

### 3.3. Power

**Fig. 1.**
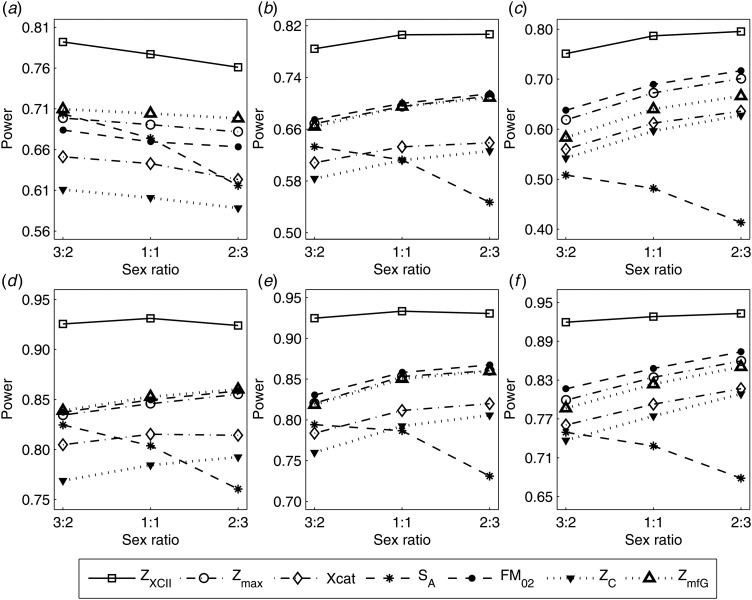
Estimated powers of *Z*_XCII_, *Z*_max_, *Xcat*, *S*_*A*_, *FM*_02_, *Z*_*C*_ and *Z*_*mfG*_ against sex ratio (*r*_*f*_ : *r*_*m*_ = 3:2, 1:1 and 2:3) under random mating when there is X-chromosome inactivation with *γ* = 1 and complete maternal parent-of-origin effects. The simulation is based on 10,000 replicates with *N* = 1000, *ϕ*_*f*0_ = *ϕ*_*m*0_ = *ϕ*_*f*01_ = 0.120 and *ϕ*_*f*10_ = *ϕ*_*f*2_ = *ϕ*_*m*1_ = 0.240. (*a*) 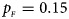, 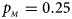. (*b*) 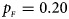, 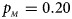. (*c*) 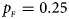, 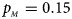. (*d*) 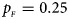, 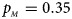. (*e*) 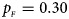, 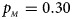. (*f*) 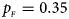, 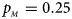.

**Fig. 2.**
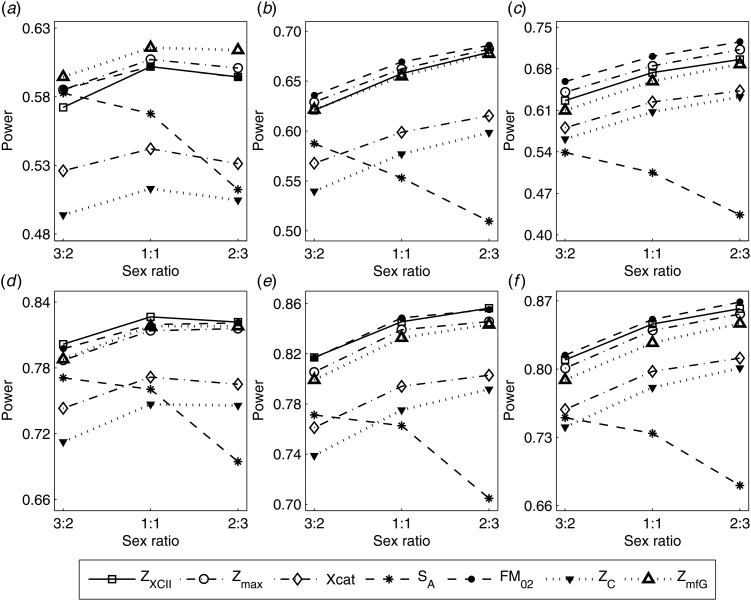
Estimated powers of *Z*_XCII_, *Z*_max_, *Xcat*, *S*_*A*_, *FM*_02_, *Z*_*C*_ and *Z*_*mfG*_ against sex ratio (*r*_*f*_ : *r*_*m*_ = 3:2, 1:1 and 2:3) under random mating when there is X-chromosome inactivation with *γ* = 1.001 and incomplete maternal parent-of-origin effects. The simulation is based on 10,000 replicates with *N* = 1000, *ϕ*_*f*0_ = *ϕ*_*m*0_ = 0.120, *ϕ*_*f*01_ = 0.144, *ϕ*_*f*10_ = 0.204 and *ϕ*_*f*2_ = *ϕ*_*m*1_ = 0.240. (*a*) 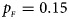, 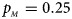. (*b*) 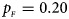, 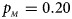. (*c*) 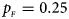, 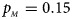. (*d*) 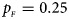, 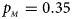. (*e*) 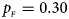, 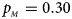. (*f*) 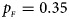, 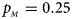.

**Fig. 3.**
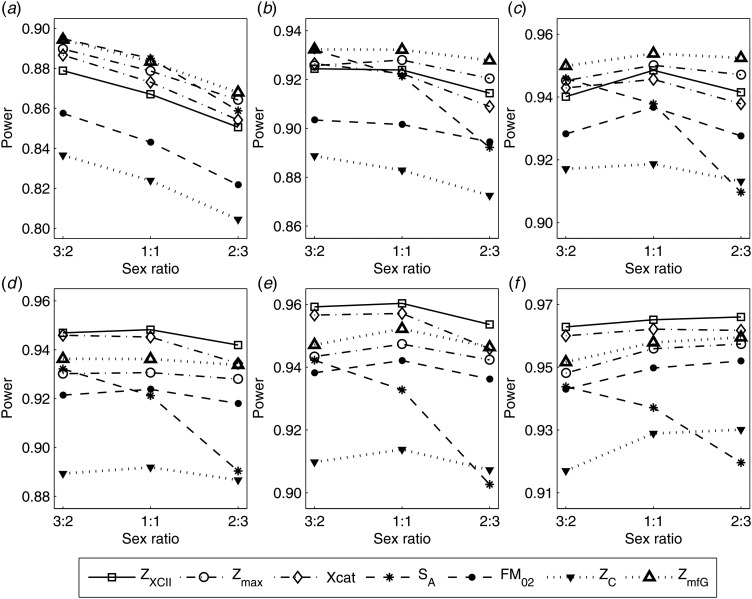
Estimated powers of *Z*_XCII_, *Z*_max_, *Xcat*, *S*_*A*_, *FM*_02_, *Z*_*C*_ and *Z*_*mfG*_ against sex ratio (*r*_*f*_ : *r*_*m*_ = 3:2, 1:1 and 2:3) under random mating when there is X-chromosome inactivation with *γ* = 2 and no parent-of-origin effects. The simulation is based on 10,000 replicates with *N* = 1000, *ϕ*_*f*0_ = *ϕ*_*m*0_ = 0.120 and *ϕ*_*f*01_ = *ϕ*_*f*10_ = *ϕ*_*f*2_ = *ϕ*_*m*1_ = 0.240. (*a*) 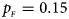, 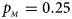. (*b*) 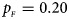, 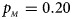. (*c*) 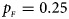, 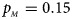. (*d*) 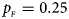, 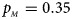. (*e*) 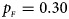, 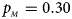. (*f*) 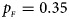, 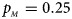.

**Fig. 4.**
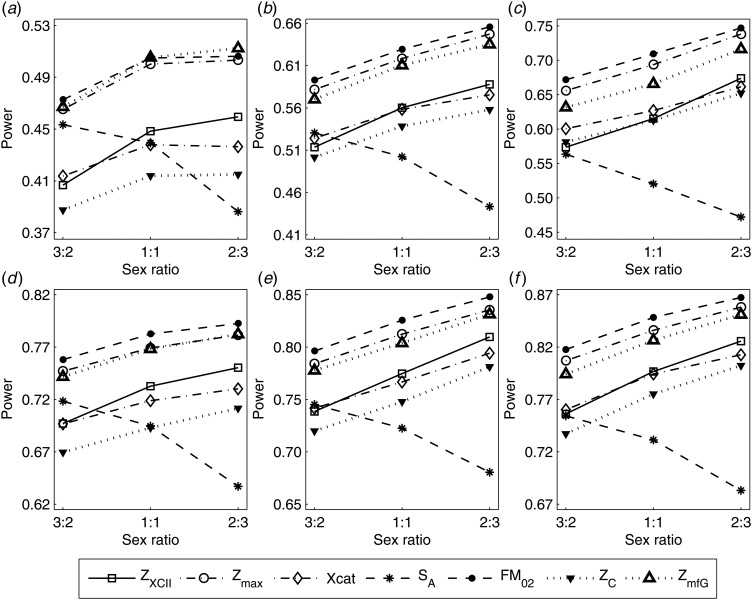
Estimated powers of *Z*_XCII_, *Z*_max_, *Xcat*, *S*_*A*_, *FM*_02_, *Z*_*C*_ and *Z*_*mfG*_ against sex ratio (*r*_*f*_ : *r*_*m*_ = 3:2, 1:1 and 2:3) under random mating when there is X-chromosome inactivation with *γ* = 0.935 and no parent-of-origin effects. The simulation is based on 10,000 replicates with *N* = 1000, *ϕ*_*f*0_ = *ϕ*_*m*0_ = 0.120, *ϕ*_*f*01_ = *ϕ*_*f*10_ = 0.168 and *ϕ*_*f*2_ = *ϕ*_*m*1_ = 0.240. (*a*) 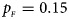, 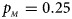. (*b*) 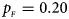, 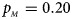. (*c*) 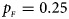, 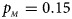. (*d*) 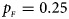, 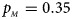. (*e*) 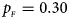, 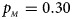. (*f*) 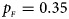, 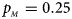.

**Fig. 5.**
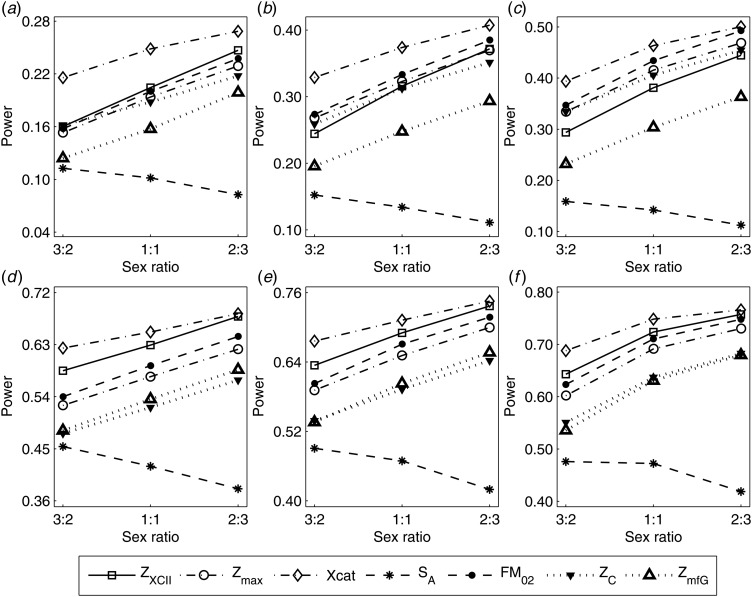
Estimated powers of *Z*_XCII_, *Z*_max_, *Xcat*, *S*_*A*_, *FM*_02_, *Z*_*C*_ and *Z*_*mfG*_ against sex ratio (*r*_*f*_ : *r*_*m*_ = 3:2, 1:1 and 2:3) under random mating when there is X-chromosome inactivation with *γ* = 0 and no parent-of-origin effects. The simulation is based on 10,000 replicates with *N* = 1000, *ϕ*_*f*0_ = *ϕ*_*m*0_ = *ϕ*_*f*01_ = *ϕ*_*f*10_ = 0.120 and *ϕ*_*f*2_ = *ϕ*_*m*1_ = 0.240. (*a*) 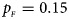, 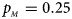. (*b*) 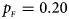, 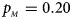. (*c*) 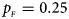, 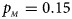. (*d*) 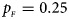, 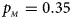. (*e*) 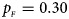, 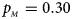. (*f*) 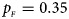, 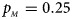.

**Fig. 6.**
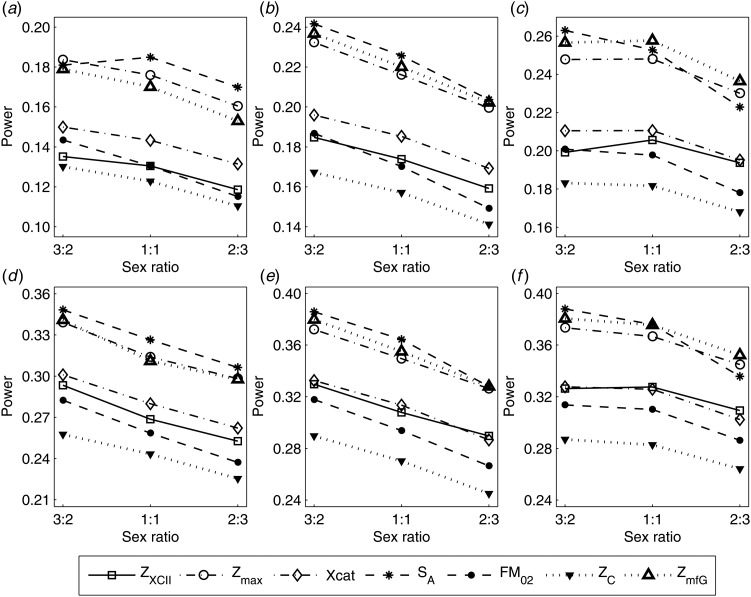
Estimated powers of *Z*_XCII_, *Z*_max_, *Xcat*, *S*_*A*_, *FM*_02_, *Z*_*C*_ and *Z*_*mfG*_ against sex ratio (*r*_*f*_ : *r*_*m*_ = 3:2, 1:1 and 2:3) under random mating when there is neither X-chromosome inactivation nor parent-of-origin effects. The simulation is based on 10,000 replicates with *N* = 1000, *ϕ*_*f*0_ = *ϕ*_*m*0_ = 0.120, *ϕ*_*f*01_ = *ϕ*_*f*10_ = *ϕ*_*m*1_ = 0.180 and *ϕ*_*f*2_ = 0.240. (*a*) 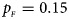, 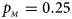. (*b*) 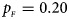, 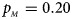. (*c*) 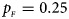, 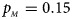. (*d*) 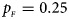, 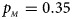. (*e*) 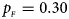, 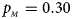. (*f*) 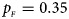, 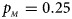.

To clearly illustrate the power results, we show the estimated powers of *Z*_XCII_, *Z*_max_, *Xcat*, *S*_*A*_, *FM*_02_, *Z*_*C*_ and *Z*_*mfG*_ with relatively better performance in Figures 1–6 and Figures S1–S22, and those of *T*_*A*_, *T*_*AD*_, 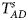, *FM*_01_, *FM*_*F*_, *Z*_*mfA*_ and *Z*_*A*_ with inflated size or lower powers are displayed in Figures S23–S50. Figure 1 gives the estimated powers of *Z*_XCII_, *Z*_max_, *Xcat*, *S*_*A*_, *FM*_02_, *Z*_*C*_ and *Z*_*mfG*_ against sex ratio under random mating when there is XCI with *γ* = 1 and complete maternal parent-of-origin effect. It is shown in Figure 1 that *Z*_XCII_ has the highest power among all seven methods. The powers of *Z*_*max*_, *FM*_02_ and *Z*_*mfG*_ are similar to each other and are generally higher than those of *Xcat*, *S*_*A*_ and *Z*_*C*_. On the other hand, the powers are influenced by the sex ratio. When the proportion of males in the case group gets larger (*r*_*f*_:*r*_*m*_ changing from 3:2 to 2:3), the power of *Z*_XCII_ becomes smaller in Figure 1(*a*), while it remains nearly unchanged in the other subplots of Figure 1, and the powers of *Z*_max_, *Xcat*, *FM*_02_, *Z*_*C*_ and *Z*_*mfG*_ are almost unchanged in Figure 1(*a*), while they are larger in the other subplots. However, with the number of males in the case group, *S*_*A*_ is less powerful. It is also found that all of the methods have higher powers with increasing allele frequency (comparing the first row with the second row). Figure 2 displays the corresponding estimated powers when there is XCI with *γ* = 1.001 and incomplete maternal parent-of-origin effect. From Figure 2, we can see that the powers of *Z*_XCII_, *Z*_max_, *FM*_02_ and *Z*_*mfG*_ are very close to each other, which are generally larger than those of *Xcat*, *S*_*A*_ and *Z*_*C*_. Compared to Figure 1, the effect of the sex ratio on *Z*_XCII_ is greater as the power of *Z*_XCII_ increases with larger male proportion in the case group in the second and third columns of Figure 2.

When there are XCI and no parent-of-origin effects under random mating, the estimated powers of *Z*_XCII_, *Z*_max_, *Xcat*, *S*_*A*_, *FM*_02_, *Z*_*C*_ and *Z*_*mfG*_ with *γ* = 2, 0.935 and 0 are shown in Figures 3–5, respectively. From Figure 3, *Z*_*mfG*_ has the highest power in the first row of Figure 3, while *Z*_XCII_ is the most powerful in the second row. In fact, the powers of *Z*_XCII_, *Z*_max_, *Xcat* and *Z*_*mfG*_ are very close to each other, which are larger than those of *FM*_02_ and *Z*_*C*_. *S*_*A*_ has relatively good performance in the first row of Figure 3, while it performs worse in the second row. In Figure 4, we find that *Z*_XCII_ generally has higher power than *Xcat*, *S*_*A*_ and *Z*_*C*_, although it has less power than *Z*_max_, *FM*_02_ and *Z*_*mfG*_. *Xcat* is always the most powerful in all of the subplots of Figure 5. In the first row of Figure 5, *Z*_XCII_, *Z*_max_, *FM*_02_ and *Z*_*C*_ have similar powers, which perform much better than *S*_*A*_ and *Z*_*mfG*_. In the second row of Figure 5, *Z*_XCII_ is more powerful than the other five methods, except for *Xcat*. Furthermore, by comparing Figures 3–5, we find that the powers get larger with increasing *γ*-value. By comparing Figure 1 (complete maternal parent-of-origin effect), Figure 2 (incomplete maternal parent-of-origin effect) and Figure 4 (no parent-of-origin effects) with *γ* being fixed close to 1 (XCI-R), the power of *Z*_XCII_ becomes smaller and smaller. Figure 6 plots the estimated powers of *Z*_XCII_, *Z*_max_, *Xcat*, *S*_*A*_, *FM*_02_, *Z*_*C*_ and *Z*_*mfG*_ against the sex ratio under random mating when there is neither XCI nor parent-of-origin effects. *Z*_XCII_ has similar power to *Xcat* and *FM*_02_ in most situations. *Z*_*max*_, *S*_*A*_ and *Z*_*mfG*_ always outperform the other methods, while the power of *Z*_*C*_ is always the lowest among those methods. The relatively low power of *Z*_*XCII*_ is due to no XCI and no parent-of-origin effects.

The power results of *Z*_XCII_, *Z*_max_, *Xcat*, *S*_*A*_, *FM*_02_, *Z*_*C*_ and *Z*_*mfG*_ with *γ* = 1.499 and 0.492 under random mating and incomplete maternal parent-of-origin effect are given in Figures S1 and S2, respectively. When there are no parent-of-origin effects, Figures S3 and S4 plot the estimated powers under XCI with *γ* = 1.503 and 0.500, respectively. The powers of these seven methods under random mating and paternal parent-of-origin effects are shown in Figures S5–S8. The results are similar to those under maternal parent-of-origin effects, except that the powers of *Z*_XCII_ seem to be more strongly affected by the difference between 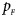 and 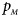 under paternal parent-of-origin effects. For example, the difference in power between Figure S5(*c*) and Figure S5(*a*) is much larger than that between Figure 1(*c*) and Figure 1(*a*).

Figures S9–S22 present the powers under the simulation settings where 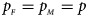 but HWE does not hold in female offspring. The left column of each figure represents the powers when *ρ* = −0.05, while the right column denotes the powers when *ρ* = 0.05. When comparing the two columns of each figure with the middle column in the corresponding figure under random mating (*ρ* = 0), we find that the powers with *ρ* = −0.05, 0 and 0.05 have similar trends, while the powers slightly increase as *ρ* changes from –0.05 to 0.05. This is probably due to the increase of genotype frequency of *A*/*A*. Finally, Figures S23–S50 display the powers of the other seven methods (*T*_*A*_, *T*_*AD*_, 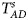, *FM*_01_, *FM*_*F*_, *Z*_*mfA*_ and *Z*_*A*_), which control the size less well or have relatively low powers.

## 4. Discussion

In this paper, we propose a robust test, *Z*_XCII_, for testing associations between certain diseases and an X-linked SNP by simultaneously accounting for XCI and parent-of-origin effects. Our proposed method is an extension of *Xcat* for the situation where parent-of-origin effects have influence on the process of XCI. Two reasonable assumptions are made for *Z*_XCII_, just like *Xcat* (Chen *et al.*, 2017): the generalized genetic model is hypothesized for female offspring and the mutant allele in female offspring is the same as that in male offspring. A good feature of the proposed method that should be emphasized is that there is no need to specify the patterns of XCI or parent-of-origin effects. The simulation studies are conducted in order to investigate the validity and performance of *Z*_XCII_ under various scenarios of parameter values. The simulation results demonstrate that *Z*_XCII_ is robust in all of the situations considered. It controls the size well and generally outperforms most of the 13 existing methods in power in the presence of parent-of-origin effects, especially complete parent-of-origin effects, although it suffers from slight loss in power when there are no parent-of-origin effects. Thus, the proposed method is a preferred choice when we are not sure whether or not there are parent-of-origin effects in practice.

It should be noted that *Z*_XCII_ is an extension of *Xcat*. We first use the Fisher's method to combine *Z*_1_, *Z*_2_ and *Z*_3_ in female offspring (denoted by *Z*_*f*_) and then obtain the proposed *Z*_XCII_ by weighting *Z*_*f*_ in female offspring and *Z*_*m*_ in male offspring, while *Xcat* applies the Fisher's method directly to incorporate the test statistics for females and males (Chen *et al.*, 2017). In fact, we have used the other methods to directly combine the test statistics for females and males, such as Fisher's approach used in Chen *et al.* (2017) and Stouffer's method (Owen, 2009). However, we find that *Z*_XCII_ is optimal for most of the situations considered. On the other hand, compared to *Xcat*, the regression-based method allows us to adjust for covariates, which is another potential advantage of the proposed method. According to the simulation results (omitted here for brevity), we also found that *Z*_XCII_ and other methods are not applicable to the association study for rare alleles. We may need to use the SKAT (Wu *et al.*, 2011) or the extensions of SKAT (Larson *et al.*, 2019) for dealing with this situation, which will be our subsequent work. In addition, note that the proposed *Z*_XCII_ is only suitable for qualitative traits. If we want to analyse quantitative traits in future, we will need to change the logistic regression to multiple linear regression and conduct simulations to compare it with existing methods for quantitative traits. Finally, just like Wang *et al.* (2014), in order to simplify our model, we assumed that XCI-E is regarded as a binary variable to distinguish whether or not XCI is present. However, many genes have been observed to be of ‘variable escape’, with the levels of escape varying between individuals, cells and tissues or over time. How to consider these variable levels of XCI-E in our model will be our future work.

## Supplementary material

For supplementary material accompanying this paper visit http://dx.doi.org/10.1017/S0016672320000026. click here to view supplementary material
