# Supplementary material for: A robust test for X-chromosome genetic association accounting for X-chromosome inactivation and imprinting
Source: Genet Res (Camb). 2020 Apr 1;102:e2. doi: 10.1017/S0016672320000026 (PMC7132553; doi:10.1017/S0016672320000026)
Supplement: Supplementary file 1 [file S0016672320000026sup001.pdf]

Genetics Research

A robust test for X-chromosome genetic association accounting for X-inactivation and imprinting

Yu Zhang[1,⋆], Si-Qi Xu[1,2,⋆], Wei Liu[1], Wing Kam Fung[2] and Ji-Yuan Zhou[1,*]

[1] State Key Laboratory of Organ Failure Research, Ministry of Education, and Guangdong Provincial Key Laboratory of Tropical Disease Research, Department of Biostatistics, School of Public Health, Southern Medical University, Guangzhou, China;

[2] Department of Statistics and Actuarial Science, The University of Hong Kong, Hong Kong, China

⋆ These authors contributed equally

* Author for correspondence: Ji-Yuan Zhou, State Key Laboratory of Organ Failure Research, Ministry of Education, and Guangdong Provincial Key Laboratory of Tropical Disease Research, Department of Biostatistics, School of Public Health, Southern Medical University, No. 1023, South Shatai Road, Baiyun District, Guangzhou 510515, China (E-mail: zhoujiyuan5460@hotmail.com).

Short title: Association test with XCI and imprinting

Supplementary Table S1. *References of existing association tests on X chromosome*

Test statistic	Reference
Z_{max}	Wang *et al.* (2019)
Xcat	Chen *et al.* (2017)
S_A	Loley *et al.* (2011)
FM_{02}	Gao *et al.* (2015)
Z_C	Zheng *et al.* (2007)
Z_{mfG}	Zheng *et al.* (2007)
T_A	Clayton (2008)
T_{AD}	Clayton (2008)
T_{AD}^s	Loley *et al.* (2011)
FM_{01}	Gao *et al.* (2015)
FM_{F}	Gao *et al.* (2015)
Z_{mfA}	Zheng *et al.* (2007)
Z_A	Zheng *et al.* (2007)

Supplementary Table S2. *Values of γ under XCI with different values of ϕ_{f01} and ϕ_{f10}.*

	ϕ_{f10}										
ϕ_{f01}	0.120	0.132	0.144	0.156	0.168	0.180	0.192	0.204	0.216	0.228	0.240
0.120	0	0.130	0.250	0.362	0.467	0.567	0.661	0.751	0.838	0.920	1
0.132	0.130	0.260	0.380	0.492	0.597	0.697	0.791	0.881	0.967	1.050	1.130
0.144	0.250	0.380	0.500	0.612	0.718	0.817	0.911	1.001	1.088	1.170	1.250
0.156	0.362	0.492	0.612	0.724	0.830	0.929	1.024	1.114	1.200	1.282	1.362
0.168	0.467	0.597	0.718	0.830	0.935	1.034	1.129	1.219	1.305	1.388	1.467
0.180	0.567	0.697	0.817	0.929	1.034	1.134	1.228	1.318	1.404	1.487	1.567
0.192	0.661	0.791	0.911	1.024	1.129	1.228	1.323	1.413	1.499	1.582	1.661
0.204	0.751	0.881	1.001	1.114	1.219	1.318	1.413	1.503	1.589	1.672	1.751
0.216	0.838	0.967	1.088	1.200	1.305	1.404	1.499	1.589	1.675	1.758	1.838
0.228	0.920	1.050	1.170	1.282	1.388	1.487	1.582	1.672	1.758	1.841	1.920
0.240	1	1.130	1.250	1.362	1.467	1.567	1.661	1.751	1.838	1.920	2

Supplementary Table S3. *Definitions of parameters*

Parameter	Definition
ρ	Inbreeding coefficient
N	Sample size
n_r	Number of cases in offspring generation
n_s	Number of controls in offspring generation
n_f	Number of daughter-parent trios
n_m	Number of male offspring
$r_f : r_m$	Sex ratio in the case group
$s_f : s_m$	Sex ratio in the control group
β_{f0}	Intercept of the regression model for females
β_{f1}	Regression coefficient for genotype a/A in the regression model for females
β_{f2}	Regression coefficient for genotype A/a in the regression model for females
β_{f3}	Regression coefficient of the interaction term in the regression model for females
β_{m0}	Intercept of the regression model for males
β_m	Regression coefficient for genotype A in the regression model for males
γ	Degree of the inactivation under X chromosome inactivation
p_F	Allele frequency of mutant allele A for females in the parental generation
p_M	Allele frequency of mutant allele A for males in the parental generation
g_{f0}	Genotype frequency of genotype a/a for females in the offspring generation
g_{f01}	Genotype frequency of genotype a/A for females in the offspring generation
g_{f10}	Genotype frequency of genotype A/a for females in the offspring generation
g_{f2}	Genotype frequency of genotype A/A for females in the offspring generation
g_{m0}	Genotype frequency of genotype a for males in the offspring generation
g_{m1}	Genotype frequency of genotype A for males in the offspring generation
ϕ_f	Disease prevalence of females in the offspring generation
ϕ_{f0}	Penetrance for females given genotype a/a in the offspring generation
ϕ_{f01}	Penetrance for females given genotype a/A in the offspring generation
ϕ_{f10}	Penetrance for females given genotype A/a in the offspring generation
ϕ_{f2}	Penetrance for females given genotype A/A in the offspring generation
ϕ_m	Disease prevalence of males in the offspring generation
ϕ_{m0}	Penetrance for males given genotype a in the offspring generation
ϕ_{m1}	Penetrance for males given genotype A in the offspring generation

Supplementary Table S4. *Biological meanings of the situations we considered ($\phi_{f0} = \phi_{m0} = 0.120$)*

$(\phi_{f01}, \phi_{f10}, \phi_{f2}, \phi_{m1})$	Meaning
$(0.120, 0.240, 0.240, 0.240)$	Random X chromosome inactivation and complete maternal parent-of-origin effect
$(0.192, 0.216, 0.240, 0.240)$	75% of the cells have the mutant allele active and incomplete maternal parent-of-origin effect
$(0.144, 0.204, 0.240, 0.240)$	Random X chromosome inactivation and incomplete maternal parent-of-origin effect
$(0.132, 0.156, 0.240, 0.240)$	25% of the cells have the mutant allele active and incomplete maternal parent-of-origin effect
$(0.240, 0.120, 0.240, 0.240)$	Random X chromosome inactivation and complete paternal parent-of-origin effect
$(0.216, 0.192, 0.240, 0.240)$	75% of the cells have the mutant allele active and incomplete paternal parent-of-origin effect
$(0.204, 0.144, 0.240, 0.240)$	Random X chromosome inactivation and incomplete paternal parent-of-origin effect
$(0.156, 0.132, 0.240, 0.240)$	25% of the cells have the mutant allele active and incomplete paternal parent-of-origin effect
$(0.240, 0.240, 0.240, 0.240)$	100% of the cells have the mutant allele active and no parent-of-origin effects
$(0.204, 0.204, 0.240, 0.240)$	75% of the cells have the mutant allele active and no parent-of-origin effects
$(0.168, 0.168, 0.240, 0.240)$	Random X chromosome inactivation and no parent-of-origin effects
$(0.144, 0.144, 0.240, 0.240)$	25% of the cells have the mutant allele active and no parent-of-origin effects
$(0.120, 0.120, 0.240, 0.240)$	100% of the cells have the normal allele active and no parent-of-origin effects
$(0.180, 0.180, 0.240, 0.180)$	Neither X chromosome inactivation nor parent-of-origin effects

Supplementary Table S5. *Estimated size* ($\times 10^{-5}$) *when* $p_F = p_M = p$ *but HWE does not hold in female offspring at significance level* $\alpha = 10^{-5}$ *based on* 10^6 *replicates.*[a]

ρ	p	Sex ratio	Z_{XCII}	$Z_{\max}$	Xcat	S_A	FM_{02}	Z_C	Z_{mfG}	T_A	T_{AD}	T^s_{AD}	FM_{01}	FM_{F}	Z_{mfA}	Z_A
-0.05	0.20	3:2	0.4	0.8	0.7	1.0	0.6	1.1	1.0	0.8	0.7	0.8	0.8	0.6	0.9	0.7
		1:1	**0.3**	0.8	1.0	0.6	0.7	**1.8**	0.7	0.8	1.1	1.0	0.5	1.2	0.4	**0.3**
		2:3	0.8	0.9	0.9	0.9	0.8	0.9	1.1	0.9	1.0	0.8	0.5	0.5	0.4	0.5
	0.30	3:2	1.0	1.2	1.2	0.9	1.5	**1.7**	1.2	1.6	**1.8**	1.4	1.0	1.5	0.8	0.7
		1:1	0.9	0.8	0.5	1.0	0.5	0.6	0.9	0.9	0.7	0.6	1.0	0.6	0.9	0.9
		2:3	**0.3**	0.5	**0.0**	0.6	0.5	0.5	0.7	0.6	**0.3**	0.4	0.5	**0.3**	0.4	**0.3**
0.05	0.20	3:2	0.6	1.2	0.8	1.0	1.0	0.8	1.2	1.2	0.9	1.0	0.7	0.6	1.1	1.0
		1:1	0.6	1.1	1.1	0.9	0.6	1.3	0.9	1.0	1.4	1.0	0.8	0.9	1.1	1.0
		2:3	0.7	0.7	0.7	1.0	0.7	0.5	1.0	0.8	0.7	0.6	0.9	0.5	1.4	1.2
	0.30	3:2	0.9	1.4	1.4	1.6	0.9	1.2	1.5	1.0	1.3	0.7	1.3	0.9	**2.3**	**2.2**
		1:1	1.3	1.4	1.3	1.5	1.1	1.6	**1.7**	1.1	1.6	1.1	1.5	1.3	**1.7**	**1.9**
		2:3	0.5	1.0	0.5	0.7	1.2	0.8	1.0	1.0	0.5	0.4	0.4	0.5	1.2	0.9

[a] Numbers that are outside of the 95% confidence interval ($0.38 \times 10^{-5}, 1.62 \times 10^{-5}$) are highlighted in bold.

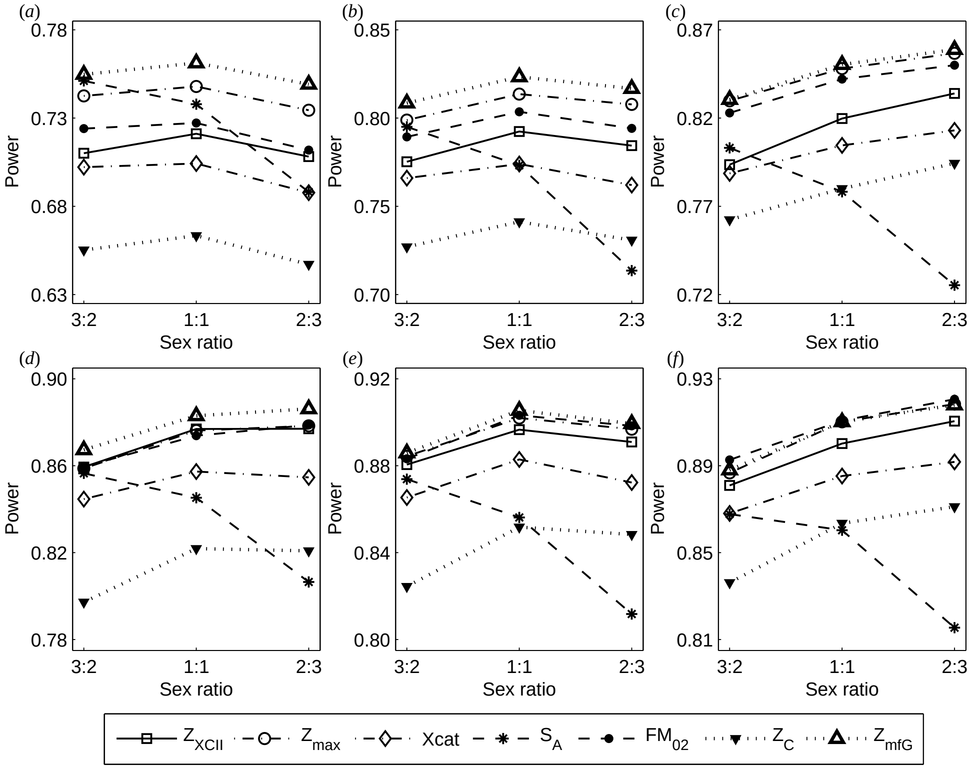

Supplementary Figure S1. Estimated powers of Z_{XCII}, $Z_{\max}$, Xcat, S_A, FM_{02}, Z_C and Z_{mfG} against sex ratio ($r_f : r_m = 3 : 2$, $1 : 1$ and $2 : 3$) under random mating when there are XCI with $\gamma = 1.499$ and incomplete maternal parent-of-origin effect. The simulation is based on 10 000 replicates with $N = 1000$, $\phi_{f0} = \phi_{m0} = 0.120$, $\phi_{f01} = 0.192$, $\phi_{f10} = 0.216$ and $\phi_{f2} = \phi_{m1} = 0.240$. (*a*) $p_F = 0.15$, $p_M = 0.25$. (*b*) $p_F = 0.20$, $p_M = 0.20$. (*c*) $p_F = 0.25$, $p_M = 0.15$. (*d*) $p_F = 0.25$, $p_M = 0.35$. (*e*) $p_F = 0.30$, $p_M = 0.30$. (*f*) $p_F = 0.35$, $p_M = 0.25$.

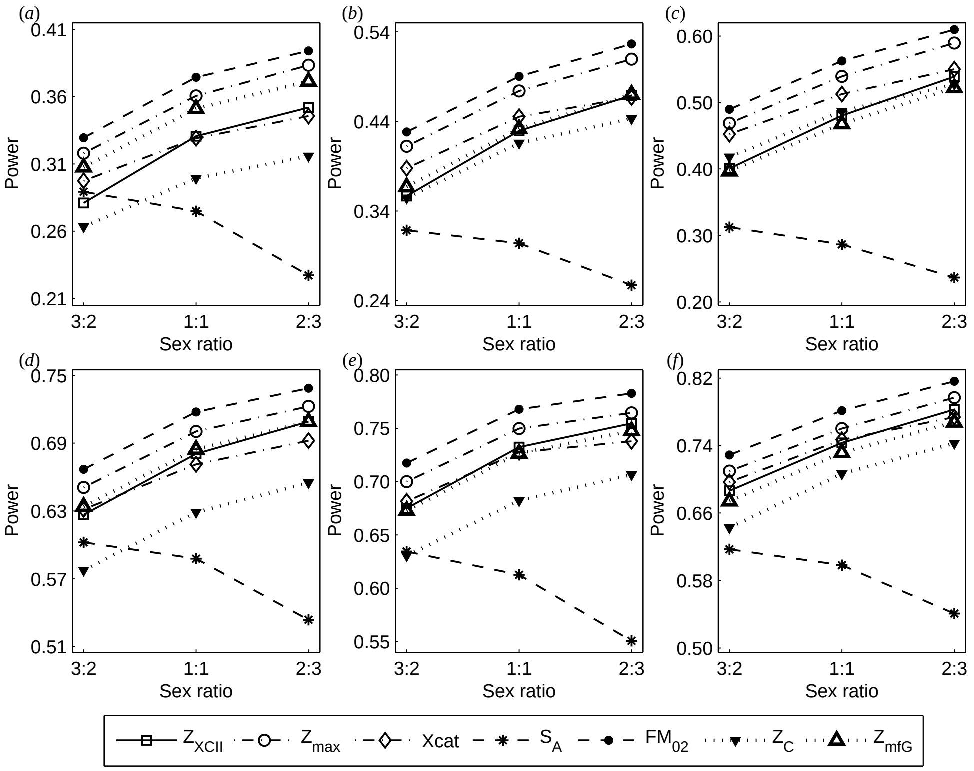

Supplementary Figure S2. Estimated powers of Z_{XCII}, $Z_{\max}$, Xcat, S_A, FM_{02}, Z_C and Z_{mfG} against sex ratio ($r_f : r_m = 3 : 2$, $1 : 1$ and $2 : 3$) under random mating when there are XCI with $\gamma = 0.492$ and incomplete maternal parent-of-origin effect. The simulation is based on 10 000 replicates with $N = 1000$, $\phi_{f0} = \phi_{m0} = 0.120$, $\phi_{f01} = 0.132$, $\phi_{f10} = 0.156$ and $\phi_{f2} = \phi_{m1} = 0.240$. (*a*) $p_F = 0.15$, $p_M = 0.25$. (*b*) $p_F = 0.20$, $p_M = 0.20$. (*c*) $p_F = 0.25$, $p_M = 0.15$. (*d*) $p_F = 0.25$, $p_M = 0.35$. (*e*) $p_F = 0.30$, $p_M = 0.30$. (*f*) $p_F = 0.35$, $p_M = 0.25$.

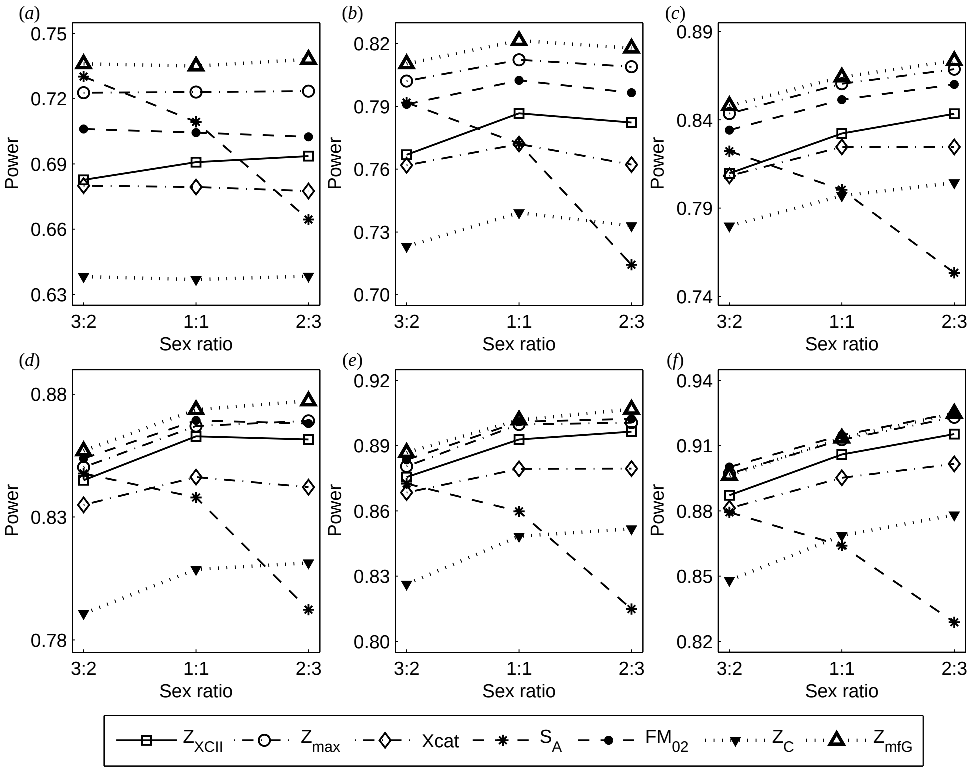

Supplementary Figure S3. Estimated powers of Z_{XCII}, $Z_{\max}$, Xcat, S_A, FM_{02}, Z_C and Z_{mfG} against sex ratio ($r_f : r_m = 3 : 2$, $1 : 1$ and $2 : 3$) under random mating when there are XCI with $\gamma = 1.503$ and no parent-of-origin effects. The simulation is based on 10 000 replicates with $N = 1000$, $\phi_{f0} = \phi_{m0} = 0.120$, $\phi_{f01} = \phi_{f10} = 0.204$ and $\phi_{f2} = \phi_{m1} = 0.240$. (a) $p_F = 0.15$, $p_M = 0.25$. (b) $p_F = 0.20$, $p_M = 0.20$. (c) $p_F = 0.25$, $p_M = 0.15$. (d) $p_F = 0.25$, $p_M = 0.35$. (e) $p_F = 0.30$, $p_M = 0.30$. (f) $p_F = 0.35$, $p_M = 0.25$.

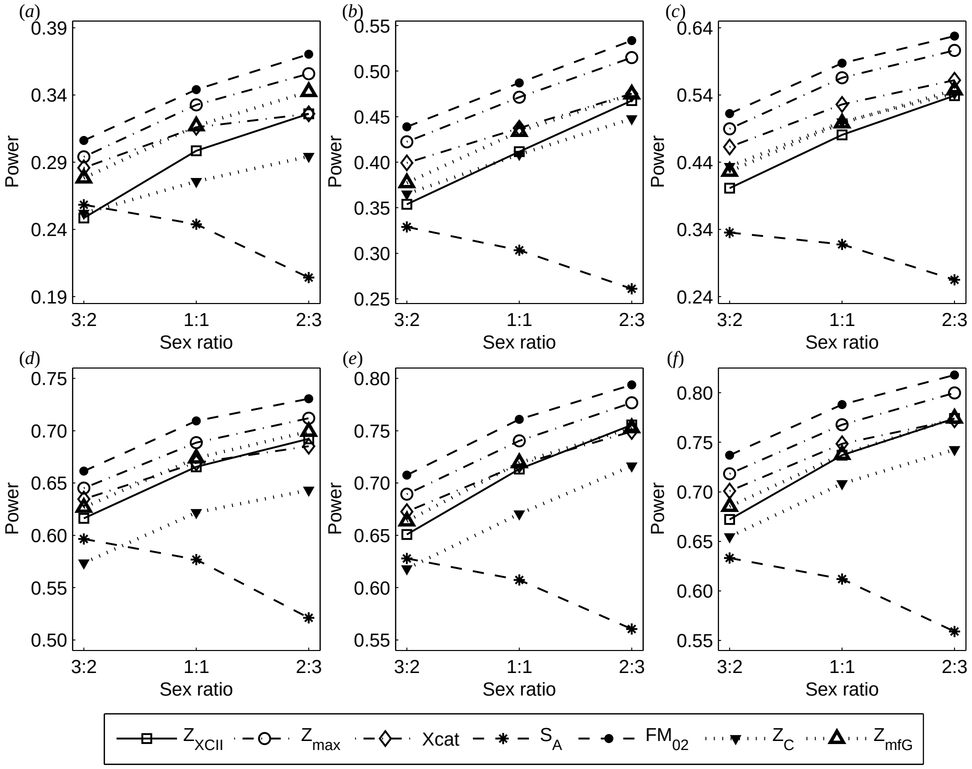

Supplementary Figure S4. Estimated powers of Z_{XCII}, $Z_{\max}$, Xcat, S_A, FM_{02}, Z_C and Z_{mfG} against sex ratio ($r_f : r_m = 3 : 2$, $1 : 1$ and $2 : 3$) under random mating when there are XCI with $\gamma = 0.500$ and no parent-of-origin effects. The simulation is based on 10 000 replicates with $N = 1000$, $\phi_{f0} = \phi_{m0} = 0.120$, $\phi_{f01} = \phi_{f10} = 0.144$ and $\phi_{f2} = \phi_{m1} = 0.240$. ($a$) $p_F = 0.15$, $p_M = 0.25$. (b) $p_F = 0.20$, $p_M = 0.20$. (c) $p_F = 0.25$, $p_M = 0.15$. (d) $p_F = 0.25$, $p_M = 0.35$. (e) $p_F = 0.30$, $p_M = 0.30$. (f) $p_F = 0.35$, $p_M = 0.25$.

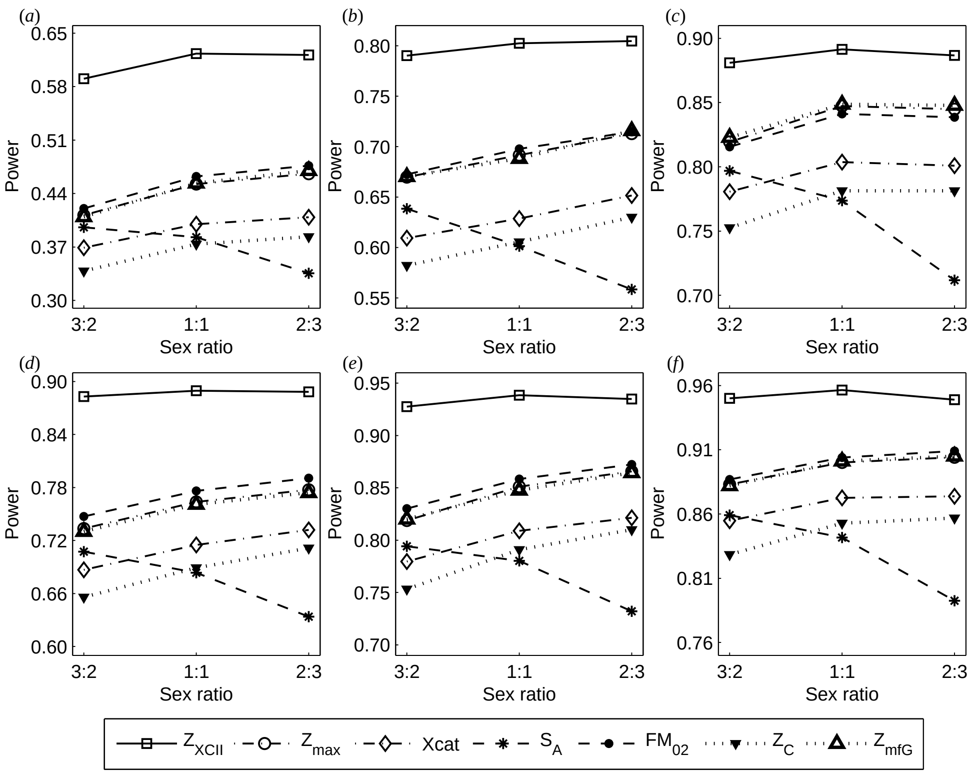

Supplementary Figure S5. Estimated powers of Z_{XCII}, $Z_{\max}$, Xcat, S_A, FM_{02}, Z_C and Z_{mfG} against sex ratio ($r_f : r_m = 3 : 2$, $1 : 1$ and $2 : 3$) under random mating when there are XCI with $\gamma = 1$ and complete paternal parent-of-origin effect. The simulation is based on 10 000 replicates with $N = 1000$, $\phi_{f0} = \phi_{m0} = \phi_{f10} = 0.120$ and $\phi_{f01} = \phi_{f2} = \phi_{m1} = 0.240$. ($a$) $p_F = 0.15$, $p_M = 0.25$. (b) $p_F = 0.20$, $p_M = 0.20$. (c) $p_F = 0.25$, $p_M = 0.15$. (d) $p_F = 0.25$, $p_M = 0.35$. (e) $p_F = 0.30$, $p_M = 0.30$. (f) $p_F = 0.35$, $p_M = 0.25$.

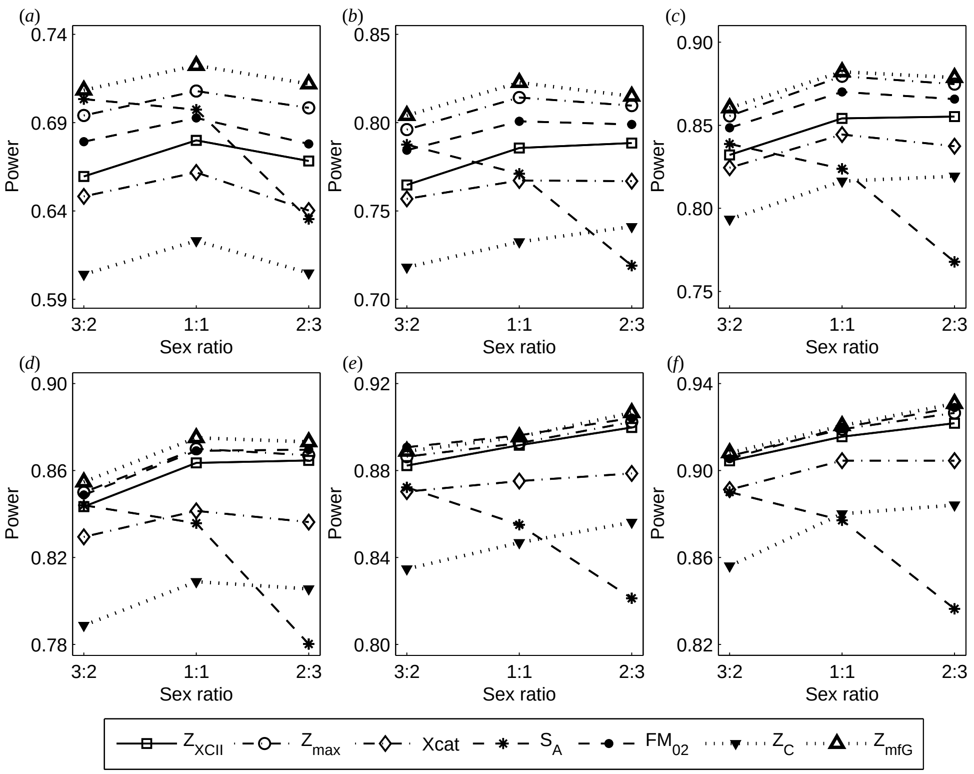

Supplementary Figure S6. Estimated powers of Z_{XCII}, $Z_{\max}$, Xcat, S_A, FM_{02}, Z_C and Z_{mfG} against sex ratio ($r_f : r_m = 3 : 2$, $1 : 1$ and $2 : 3$) under random mating when there are XCI with $\gamma = 1.499$ and incomplete paternal parent-of-origin effect. The simulation is based on 10 000 replicates with $N = 1000$, $\phi_{f0} = \phi_{m0} = 0.120$, $\phi_{f01} = 0.216$, $\phi_{f10} = 0.192$ and $\phi_{f2} = \phi_{m1} = 0.240$. (*a*) $p_F = 0.15$, $p_M = 0.25$. (*b*) $p_F = 0.20$, $p_M = 0.20$. (*c*) $p_F = 0.25$, $p_M = 0.15$. (*d*) $p_F = 0.25$, $p_M = 0.35$. (*e*) $p_F = 0.30$, $p_M = 0.30$. (*f*) $p_F = 0.35$, $p_M = 0.25$.

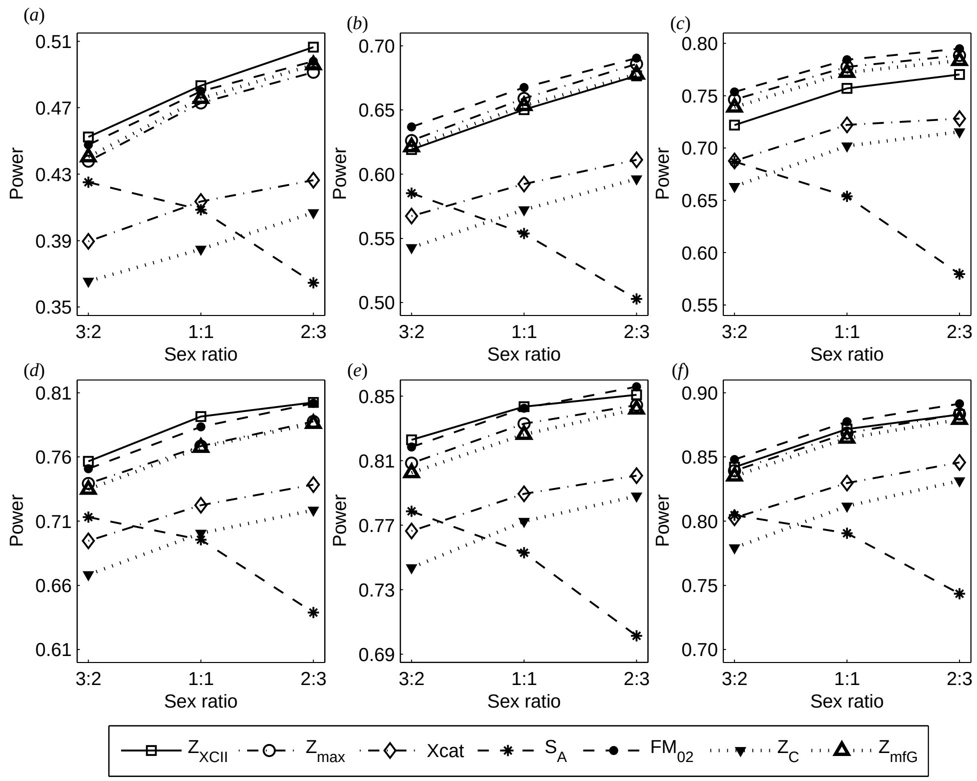

Supplementary Figure S7. Estimated powers of Z_{XCII}, $Z_{\max}$, Xcat, S_A, FM_{02}, Z_C and Z_{mfG} against sex ratio ($r_f : r_m = 3 : 2$, $1 : 1$ and $2 : 3$) under random mating when there are XCI with $\gamma = 1.001$ and incomplete paternal parent-of-origin effect. The simulation is based on 10 000 replicates with $N = 1000$, $\phi_{f0} = \phi_{m0} = 0.120$, $\phi_{f01} = 0.204$, $\phi_{f10} = 0.144$ and $\phi_{f2} = \phi_{m1} = 0.240$. (a) $p_F = 0.15$, $p_M = 0.25$. (b) $p_F = 0.20$, $p_M = 0.20$. (c) $p_F = 0.25$, $p_M = 0.15$. (d) $p_F = 0.25$, $p_M = 0.35$. (e) $p_F = 0.30$, $p_M = 0.30$. (f) $p_F = 0.35$, $p_M = 0.25$.

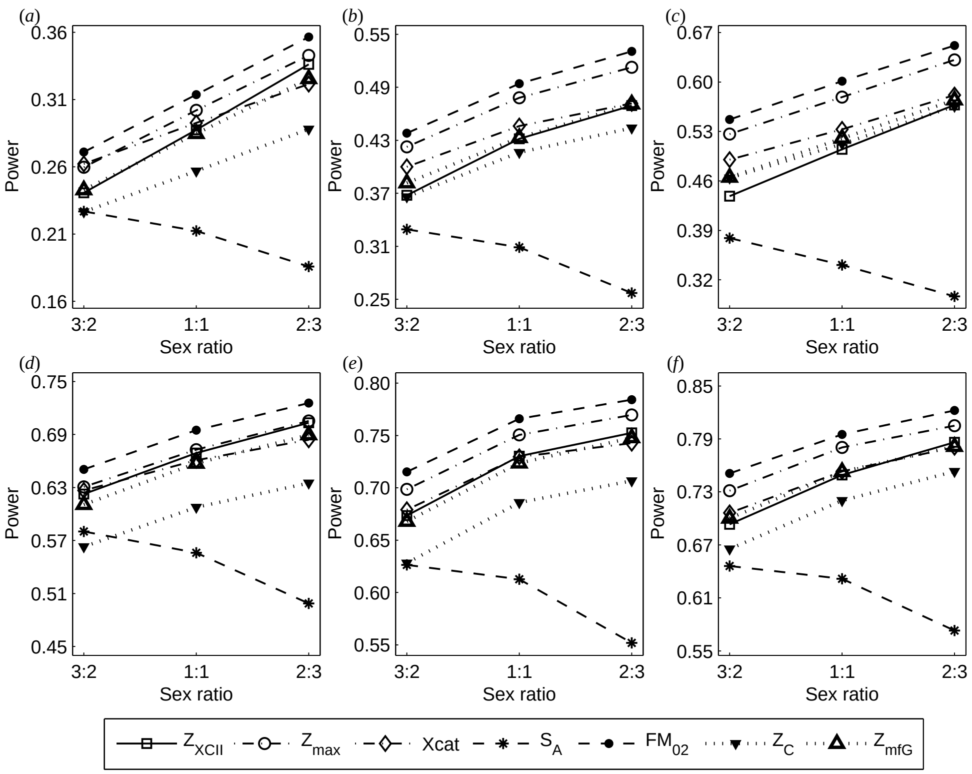

Supplementary Figure S8. Estimated powers of Z_{XCII}, $Z_{\max}$, Xcat, S_A, FM_{02}, Z_C and Z_{mfG} against sex ratio ($r_f : r_m = 3 : 2$, $1 : 1$ and $2 : 3$) under random mating when there are XCI with $\gamma = 0.492$ and incomplete paternal parent-of-origin effect. The simulation is based on 10 000 replicates with $N = 1000$, $\phi_{f0} = \phi_{m0} = 0.120$, $\phi_{f01} = 0.156$, $\phi_{f10} = 0.132$ and $\phi_{f2} = \phi_{m1} = 0.240$. ($a$) $p_F = 0.15$, $p_M = 0.25$. (b) $p_F = 0.20$, $p_M = 0.20$. (c) $p_F = 0.25$, $p_M = 0.15$. (d) $p_F = 0.25$, $p_M = 0.35$. (e) $p_F = 0.30$, $p_M = 0.30$. (f) $p_F = 0.35$, $p_M = 0.25$.

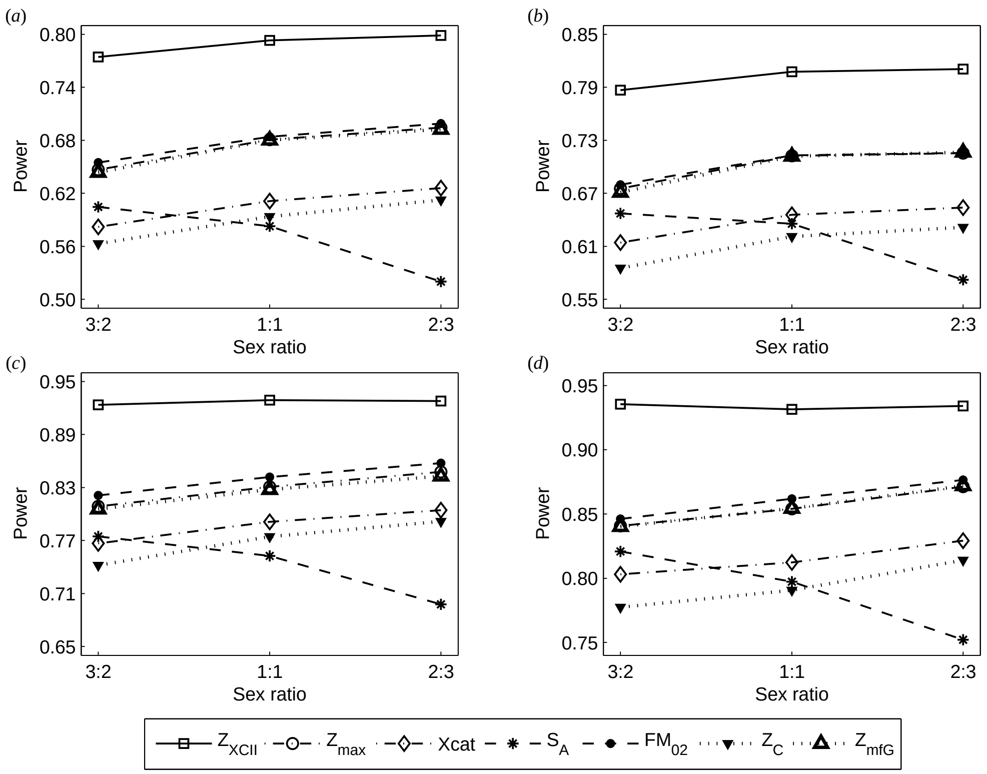

Supplementary Figure S9. Estimated powers of Z_{XCII}, $Z_{\max}$, Xcat, S_A, FM_{02}, Z_C and Z_{mfG} against sex ratio ($r_f : r_m = 3:2$, $1:1$ and $2:3$) under the situation where $p_F = p_M = p$ but HWE does not hold in female offspring when there are XCI with $\gamma = 1$ and complete maternal parent-of-origin effect. The simulation is based on 10 000 replicates with $N = 1000$, $\phi_{f0} = \phi_{m0} = \phi_{f01} = 0.120$ and $\phi_{f10} = \phi_{f2} = \phi_{m1} = 0.240$. ($a$) $p = 0.2$, $\rho = -0.05$. (b) $p = 0.2$, $\rho = 0.05$. (c) $p = 0.3$, $\rho = -0.05$. (d) $p = 0.3$, $\rho = 0.05$.

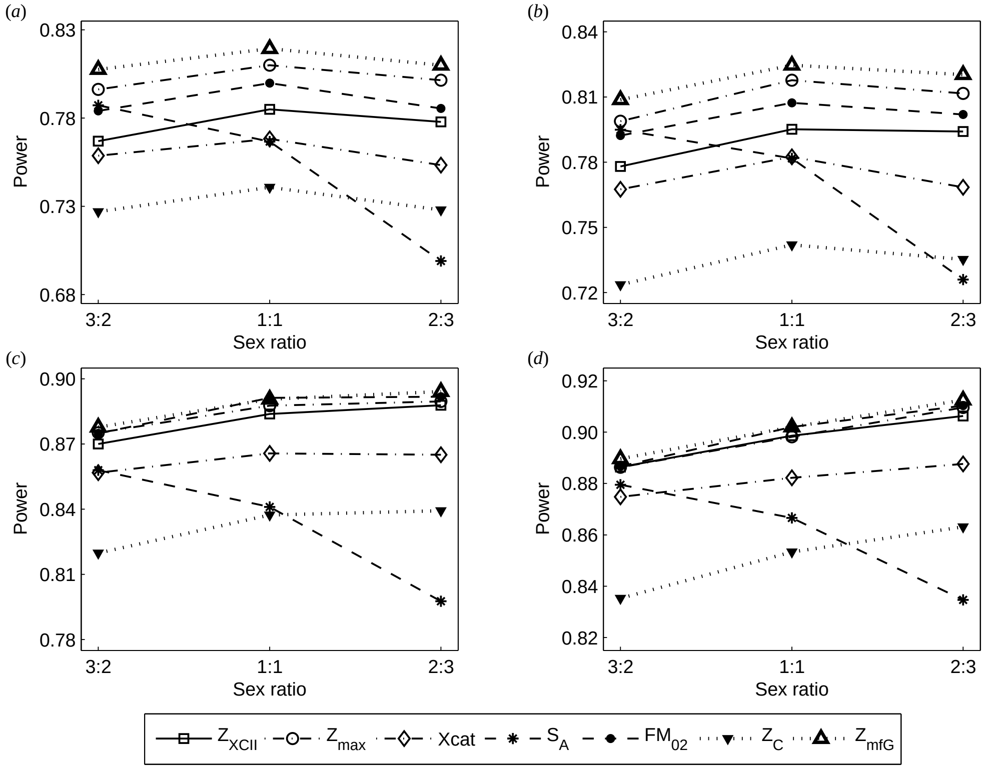

Supplementary Figure S10. Estimated powers of Z_{XCII}, $Z_{\max}$, Xcat, S_A, FM_{02}, Z_C and Z_{mfG} against sex ratio ($r_f : r_m = 3:2$, $1:1$ and $2:3$) under the situation where $p_{_F} = p_{_M} = p$ but HWE does not hold in female offspring when there are XCI with $\gamma = 1.499$ and incomplete maternal parent-of-origin effect. The simulation is based on 10 000 replicates with $N = 1000$, $\phi_{f0} = \phi_{m0} = 0.120$, $\phi_{f01} = 0.192$, $\phi_{f10} = 0.216$ and $\phi_{f2} = \phi_{m1} = 0.240$. ($a$) $p = 0.2$, $\rho = -0.05$. (b) $p = 0.2$, $\rho = 0.05$. (c) $p = 0.3$, $\rho = -0.05$. (d) $p = 0.3$, $\rho = 0.05$.

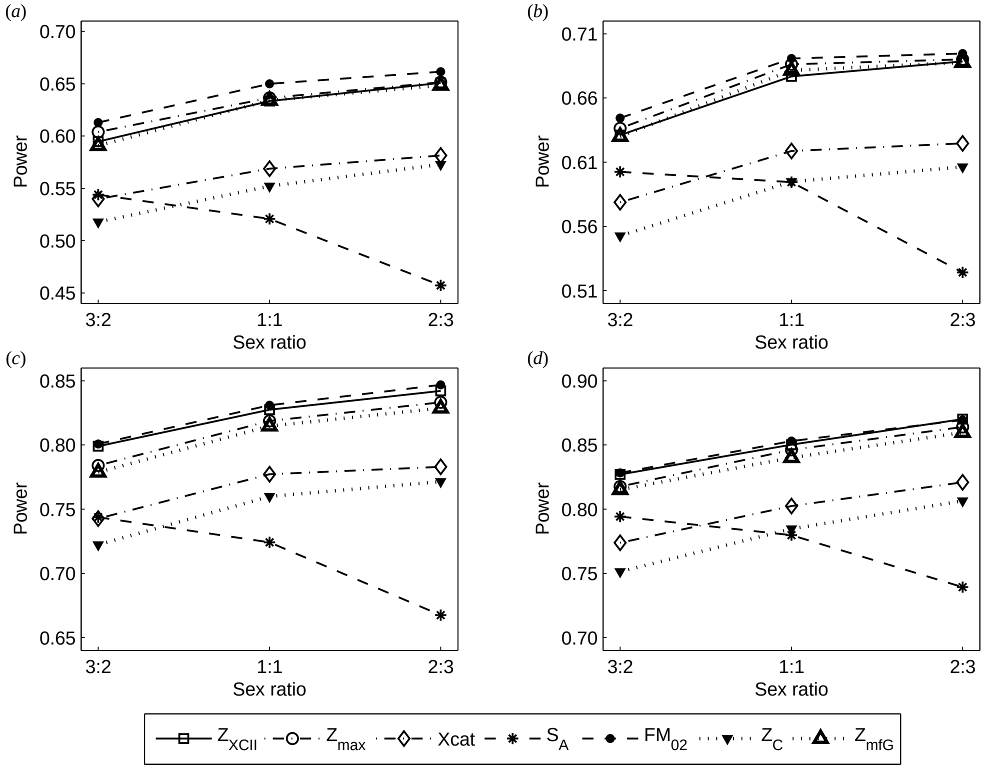

Supplementary Figure S11. Estimated powers of Z_{XCII}, $Z_{\max}$, Xcat, S_A, FM_{02}, Z_C and Z_{mfG} against sex ratio ($r_f : r_m = 3 : 2$, $1 : 1$ and $2 : 3$) under the situation where $p_{\scriptscriptstyle F} = p_{\scriptscriptstyle M} = p$ but HWE does not hold in female offspring when there are XCI with $\gamma = 1.001$ and incomplete maternal parent-of-origin effect. The simulation is based on 10 000 replicates with $N = 1000$, $\phi_{f0} = \phi_{m0} = 0.120$, $\phi_{f01} = 0.144$, $\phi_{f10} = 0.204$ and $\phi_{f2} = \phi_{m1} = 0.240$. ($a$) $p = 0.2$, $\rho = -0.05$. (b) $p = 0.2$, $\rho = 0.05$. (c) $p = 0.3$, $\rho = -0.05$. (d) $p = 0.3$, $\rho = 0.05$.

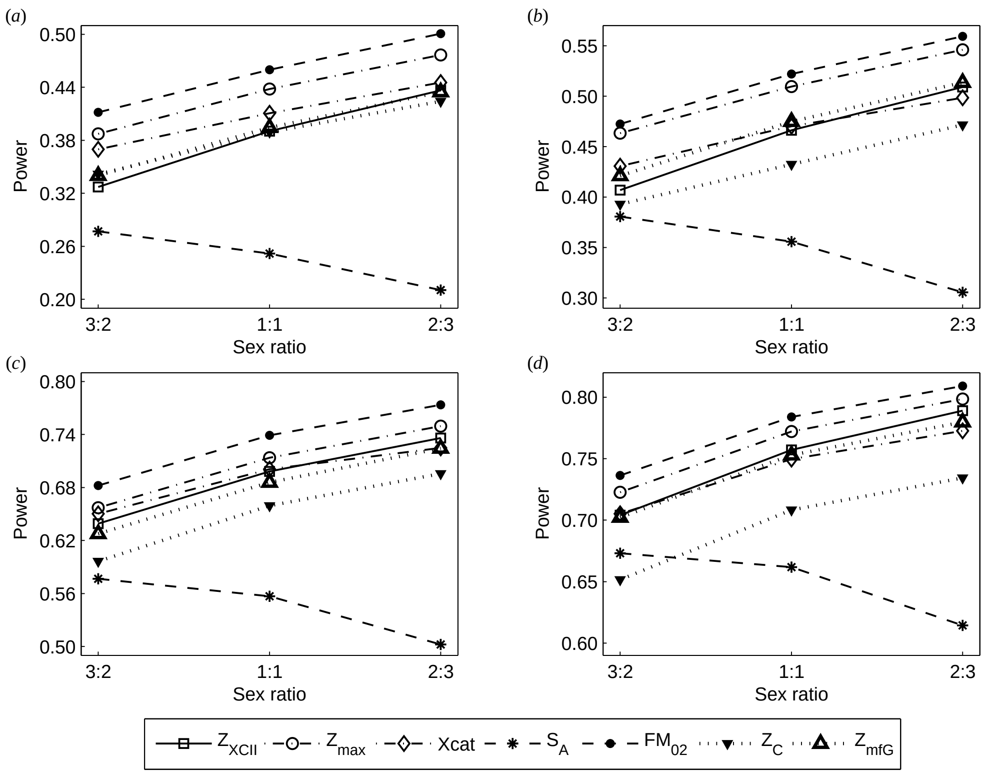

Supplementary Figure S12. Estimated powers of Z_{XCII}, $Z_{\max}$, Xcat, S_A, FM_{02}, Z_C and Z_{mfG} against sex ratio ($r_f : r_m = 3:2$, $1:1$ and $2:3$) under the situation where $p_F = p_M = p$ but HWE does not hold in female offspring when there are XCI with $\gamma = 0.492$ and incomplete maternal parent-of-origin effect. The simulation is based on 10 000 replicates with $N = 1000$, $\phi_{f0} = \phi_{m0} = 0.120$, $\phi_{f01} = 0.132$, $\phi_{f10} = 0.156$ and $\phi_{f2} = \phi_{m1} = 0.240$. ($a$) $p = 0.2$, $\rho = -0.05$. (b) $p = 0.2$, $\rho = 0.05$. (c) $p = 0.3$, $\rho = -0.05$. (d) $p = 0.3$, $\rho = 0.05$.

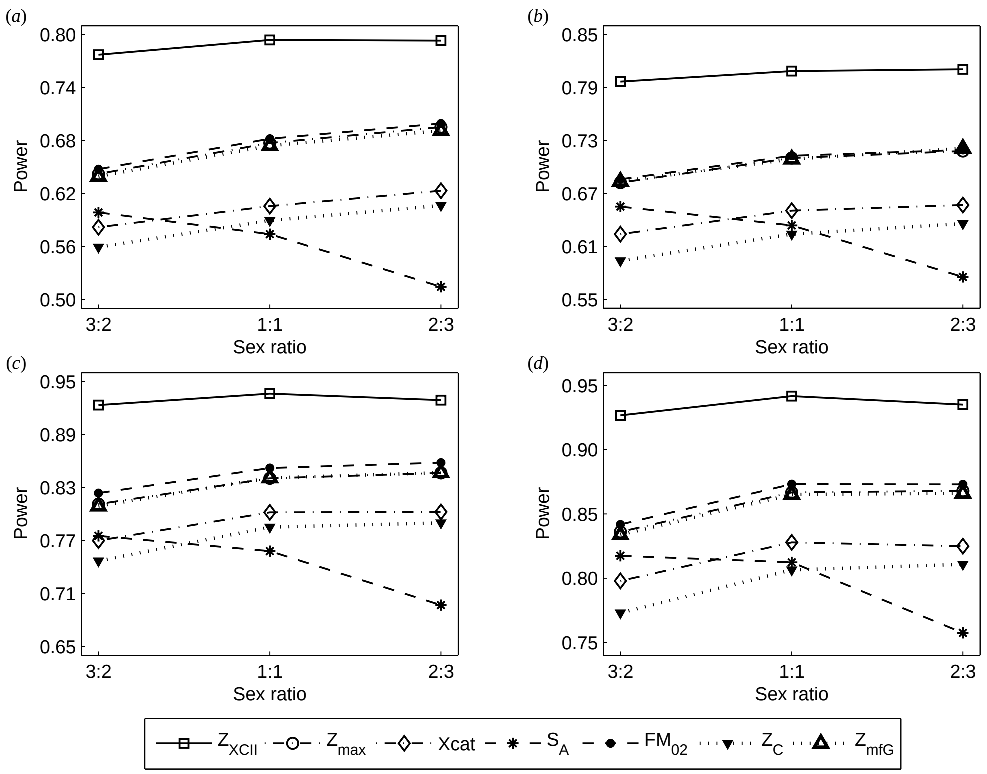

Supplementary Figure S13. Estimated powers of Z_{XCII}, $Z_{\max}$, Xcat, S_A, FM_{02}, Z_C and Z_{mfG} against sex ratio ($r_f : r_m = 3 : 2$, $1 : 1$ and $2 : 3$) under the situation where $p_{_F} = p_{_M} = p$ but HWE does not hold in female offspring when there are XCI with $\gamma = 1$ and complete paternal parent-of-origin effect. The simulation is based on 10 000 replicates with $N = 1000$, $\phi_{f0} = \phi_{m0} = \phi_{f10} = 0.120$ and $\phi_{f01} = \phi_{f2} = \phi_{m1} = 0.240$. ($a$) $p = 0.2$, $\rho = -0.05$. (b) $p = 0.2$, $\rho = 0.05$. (c) $p = 0.3$, $\rho = -0.05$. (d) $p = 0.3$, $\rho = 0.05$.

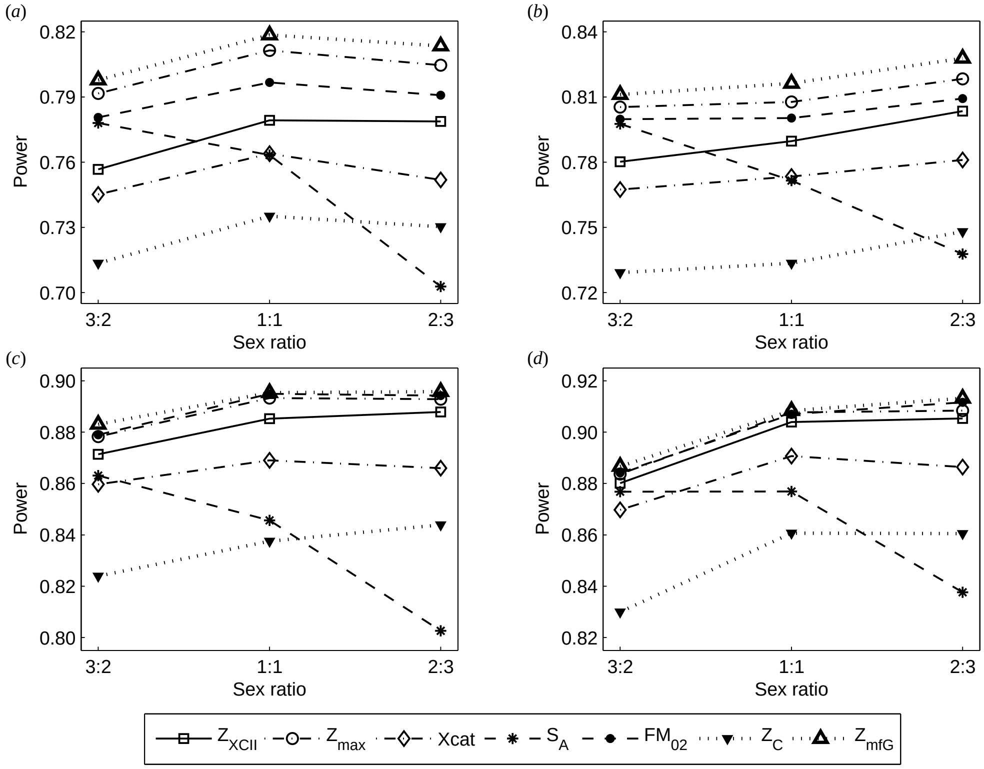

Supplementary Figure S14. Estimated powers of Z_{XCII}, $Z_{\max}$, Xcat, S_A, FM_{02}, Z_C and Z_{mfG} against sex ratio ($r_f : r_m = 3 : 2$, $1 : 1$ and $2 : 3$) under the situation where $p_{_F} = p_{_M} = p$ but HWE does not hold in female offspring when there are XCI with $\gamma = 1.499$ and incomplete paternal parent-of-origin effect. The simulation is based on 10 000 replicates with $N = 1000$, $\phi_{f0} = \phi_{m0} = 0.120$, $\phi_{f01} = 0.216$, $\phi_{f10} = 0.192$ and $\phi_{f2} = \phi_{m1} = 0.240$. ($a$) $p = 0.2$, $\rho = -0.05$. (b) $p = 0.2$, $\rho = 0.05$. (c) $p = 0.3$, $\rho = -0.05$. (d) $p = 0.3$, $\rho = 0.05$.

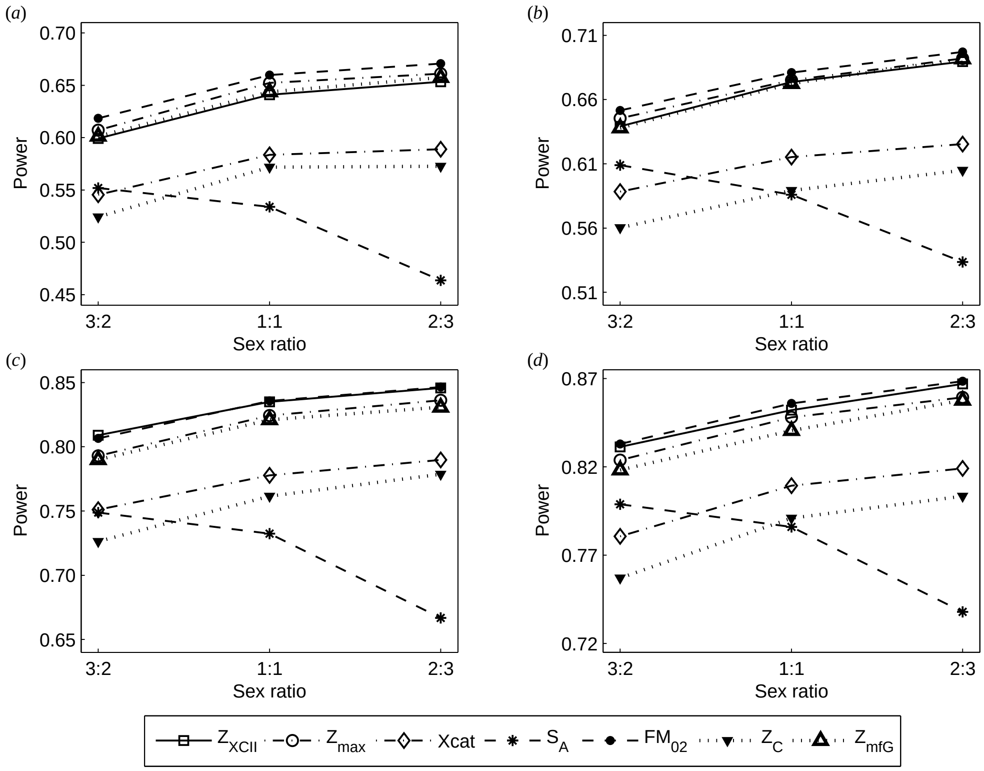

Supplementary Figure S15. Estimated powers of Z_{XCII}, $Z_{\max}$, Xcat, S_A, FM_{02}, Z_C and Z_{mfG} against sex ratio ($r_f : r_m = 3:2$, $1:1$ and $2:3$) under the situation where $p_{_F} = p_{_M} = p$ but HWE does not hold in female offspring when there are XCI with $\gamma = 1.001$ and incomplete paternal parent-of-origin effect. The simulation is based on 10 000 replicates with $N = 1000$, $\phi_{f0} = \phi_{m0} = 0.120$, $\phi_{f01} = 0.204$, $\phi_{f10} = 0.144$ and $\phi_{f2} = \phi_{m1} = 0.240$. ($a$) $p = 0.2$, $\rho = -0.05$. (b) $p = 0.2$, $\rho = 0.05$. (c) $p = 0.3$, $\rho = -0.05$. (d) $p = 0.3$, $\rho = 0.05$.

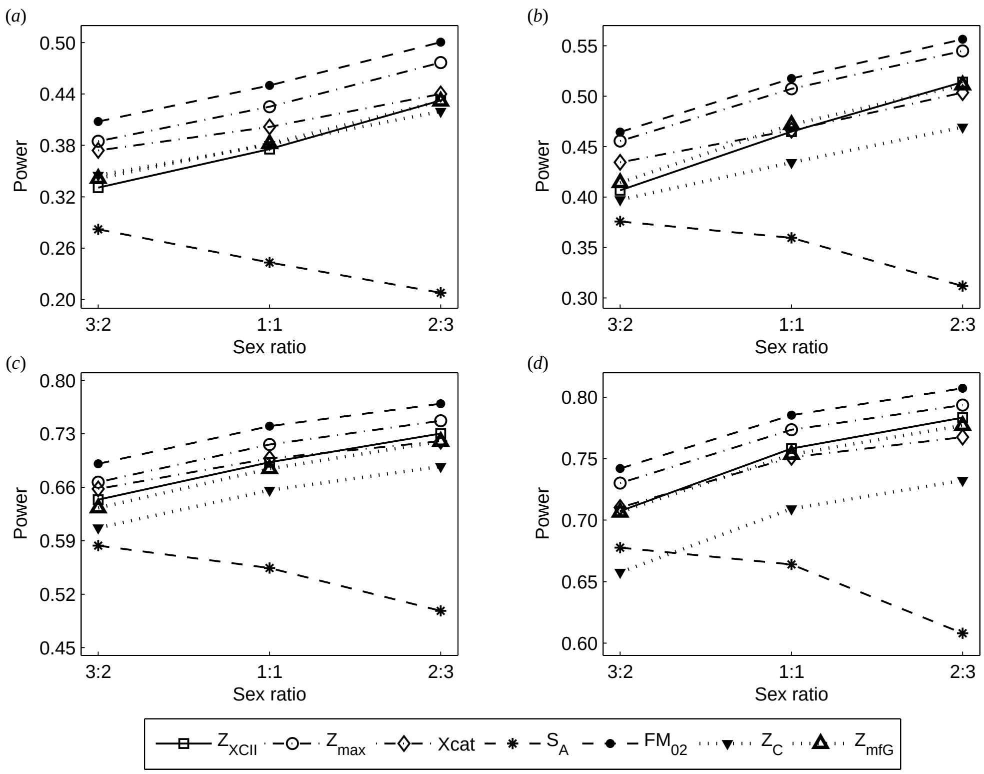

Supplementary Figure S16. Estimated powers of Z_{XCII}, $Z_{\max}$, Xcat, S_A, FM_{02}, Z_C and Z_{mfG} against sex ratio ($r_f : r_m = 3 : 2$, $1 : 1$ and $2 : 3$) under the situation where $p_{_F} = p_{_M} = p$ but HWE does not hold in female offspring when there are XCI with $\gamma = 0.492$ and incomplete paternal parent-of-origin effect. The simulation is based on 10 000 replicates with $N = 1000$, $\phi_{f0} = \phi_{m0} = 0.120$, $\phi_{f01} = 0.156$, $\phi_{f10} = 0.132$ and $\phi_{f2} = \phi_{m1} = 0.240$. ($a$) $p = 0.2$, $\rho = -0.05$. (b) $p = 0.2$, $\rho = 0.05$. (c) $p = 0.3$, $\rho = -0.05$. (d) $p = 0.3$, $\rho = 0.05$.

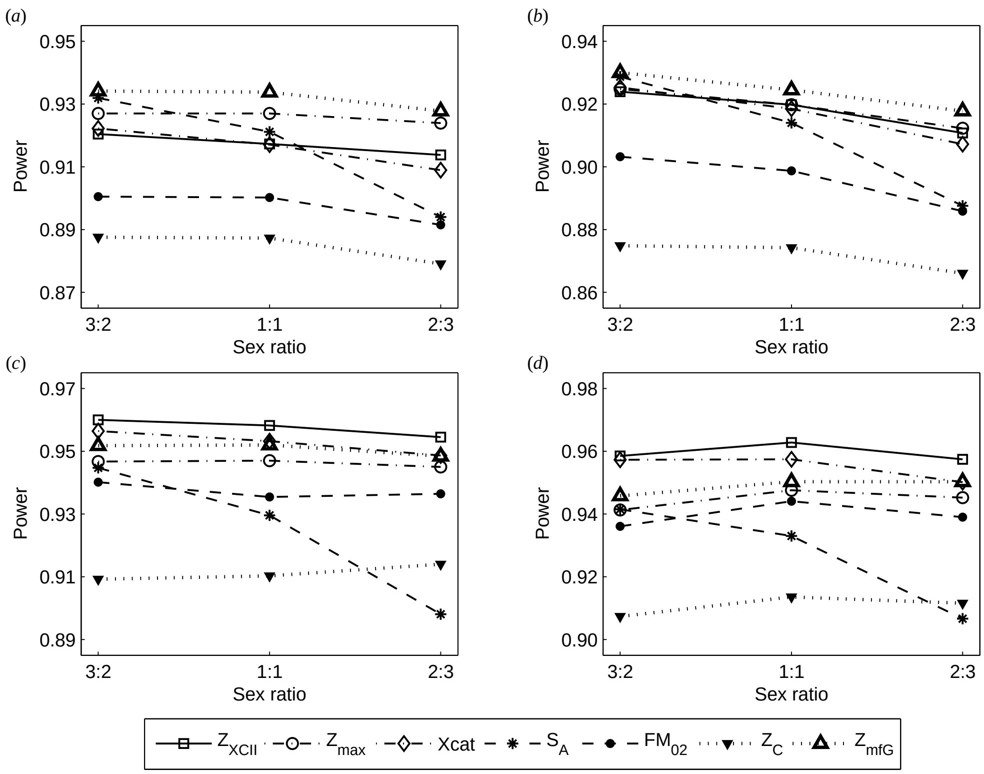

Supplementary Figure S17. Estimated powers of Z_{XCII}, $Z_{\max}$, Xcat, S_A, FM_{02}, Z_C and Z_{mfG} against sex ratio ($r_f : r_m = 3 : 2$, $1 : 1$ and $2 : 3$) under the situation where $p_{_F} = p_{_M} = p$ but HWE does not hold in female offspring when there are XCI with $\gamma = 2$ and no parent-of-origin effects. The simulation is based on 10 000 replicates with $N = 1000$, $\phi_{f0} = \phi_{m0} = 0.120$ and $\phi_{f01} = \phi_{f10} = \phi_{f2} = \phi_{m1} = 0.240$. (*a*) $p = 0.2$, $\rho = -0.05$. (*b*) $p = 0.2$, $\rho = 0.05$. (*c*) $p = 0.3$, $\rho = -0.05$. (*d*) $p = 0.3$, $\rho = 0.05$.

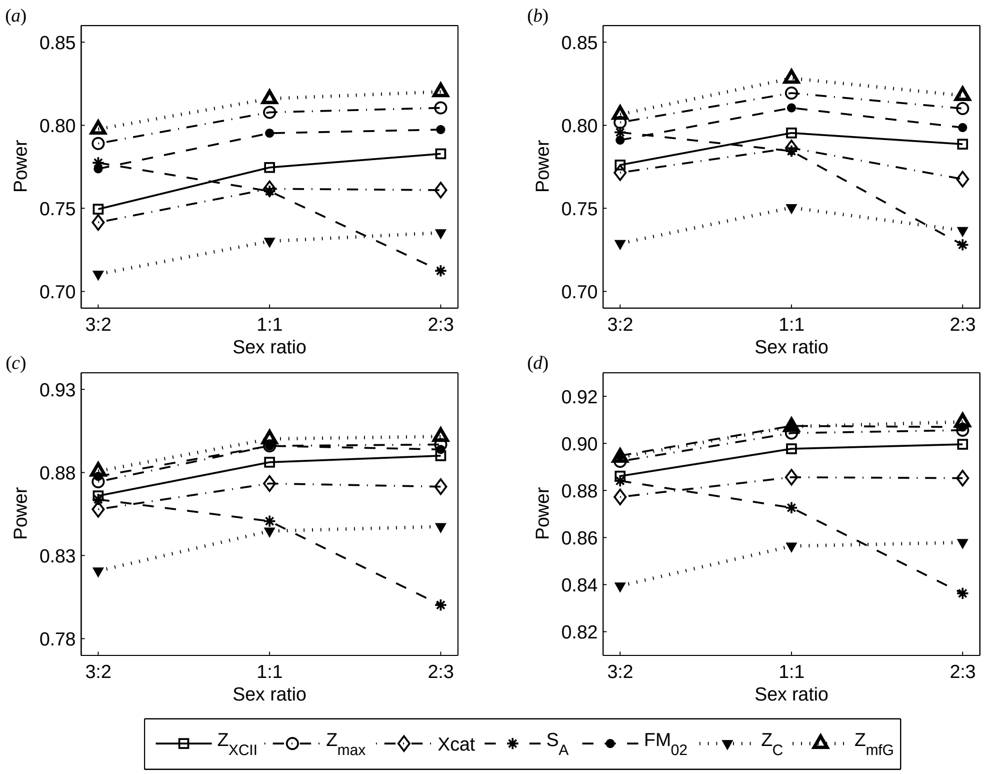

Supplementary Figure S18. Estimated powers of Z_{XCII}, $Z_{\max}$, Xcat, S_A, FM_{02}, Z_C and Z_{mfG} against sex ratio ($r_f : r_m = 3:2$, $1:1$ and $2:3$) under the situation where $p_{_F} = p_{_M} = p$ but HWE does not hold in female offspring when there are XCI with $\gamma = 1.503$ and no parent-of-origin effects. The simulation is based on 10 000 replicates with $N = 1000$, $\phi_{f0} = \phi_{m0} = 0.120$, $\phi_{f01} = \phi_{f10} = 0.204$ and $\phi_{f2} = \phi_{m1} = 0.240$. ($a$) $p = 0.2$, $\rho = -0.05$. (b) $p = 0.2$, $\rho = 0.05$. (c) $p = 0.3$, $\rho = -0.05$. (d) $p = 0.3$, $\rho = 0.05$.

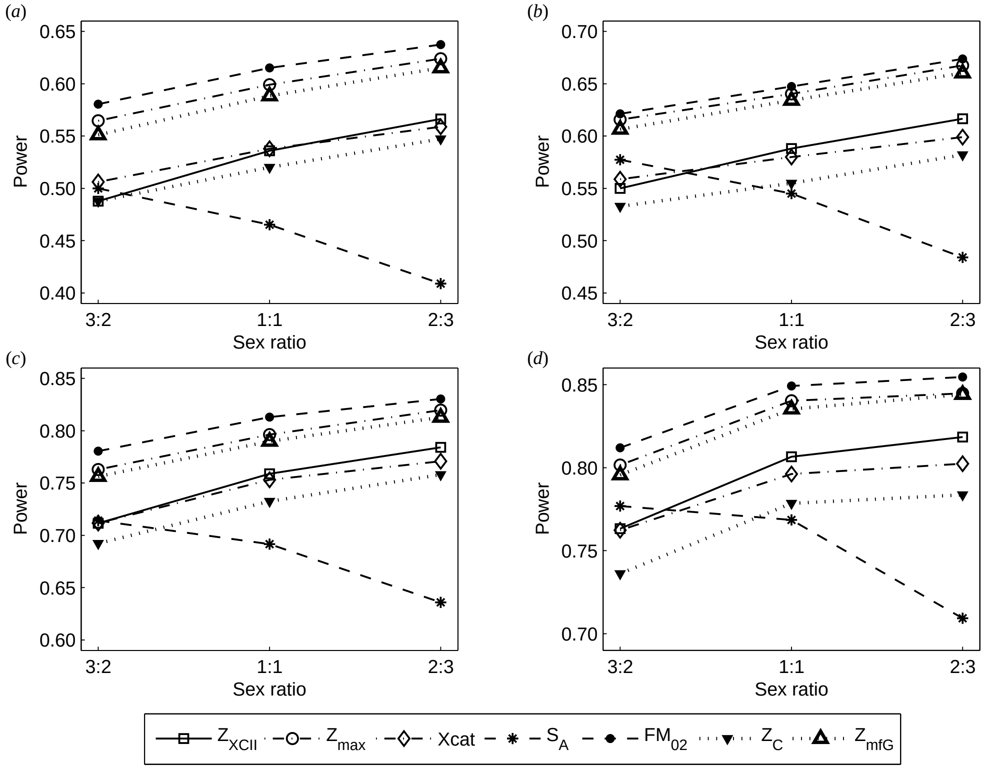

Supplementary Figure S19. Estimated powers of Z_{XCII}, $Z_{\max}$, Xcat, S_A, FM_{02}, Z_C and Z_{mfG} against sex ratio ($r_f : r_m = 3:2$, $1:1$ and $2:3$) under the situation where $p_{_F} = p_{_M} = p$ but HWE does not hold in female offspring when there are XCI with $\gamma = 0.935$ and no parent-of-origin effects. The simulation is based on 10 000 replicates with $N = 1000$, $\phi_{f0} = \phi_{m0} = 0.120$, $\phi_{f01} = \phi_{f10} = 0.168$ and $\phi_{f2} = \phi_{m1} = 0.240$. ($a$) $p = 0.2$, $\rho = -0.05$. (b) $p = 0.2$, $\rho = 0.05$. (c) $p = 0.3$, $\rho = -0.05$. (d) $p = 0.3$, $\rho = 0.05$.

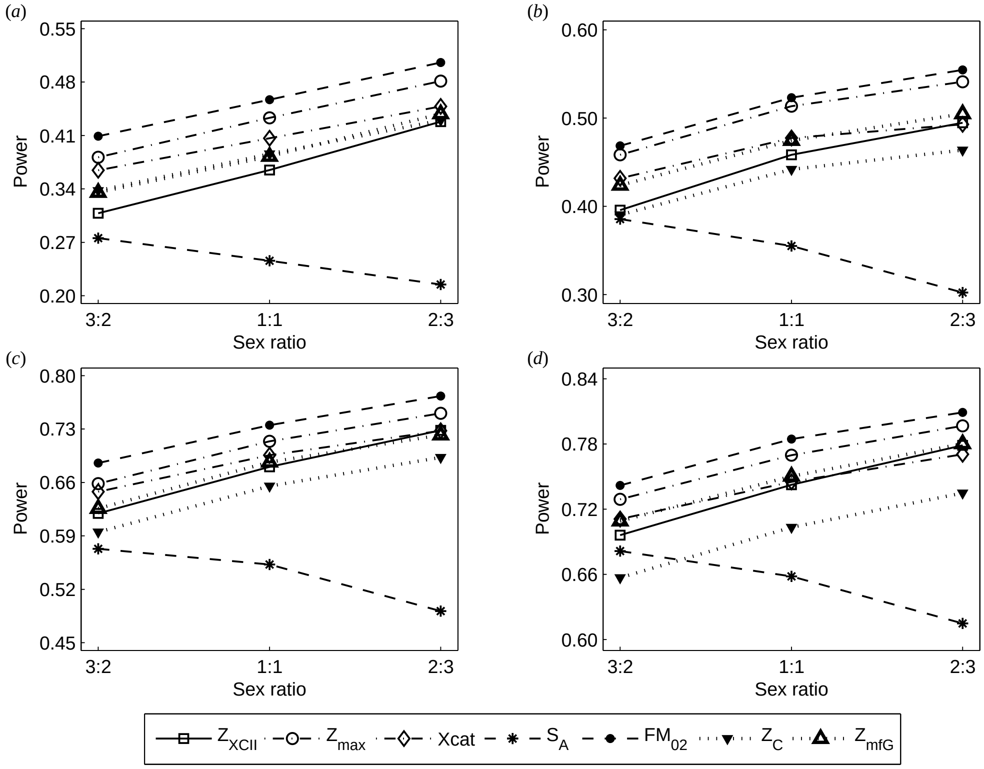

Supplementary Figure S20. Estimated powers of Z_{XCII}, $Z_{\max}$, Xcat, S_A, FM_{02}, Z_C and Z_{mfG} against sex ratio ($r_f : r_m = 3:2$, $1:1$ and $2:3$) under the situation where $p_{_F} = p_{_M} = p$ but HWE does not hold in female offspring when there are XCI with $\gamma = 0.500$ and no parent-of-origin effects. The simulation is based on 10 000 replicates with $N = 1000$, $\phi_{f0} = \phi_{m0} = 0.120$, $\phi_{f01} = \phi_{f10} = 0.144$ and $\phi_{f2} = \phi_{m1} = 0.240$. ($a$) $p = 0.2$, $\rho = -0.05$. (b) $p = 0.2$, $\rho = 0.05$. (c) $p = 0.3$, $\rho = -0.05$. (d) $p = 0.3$, $\rho = 0.05$.

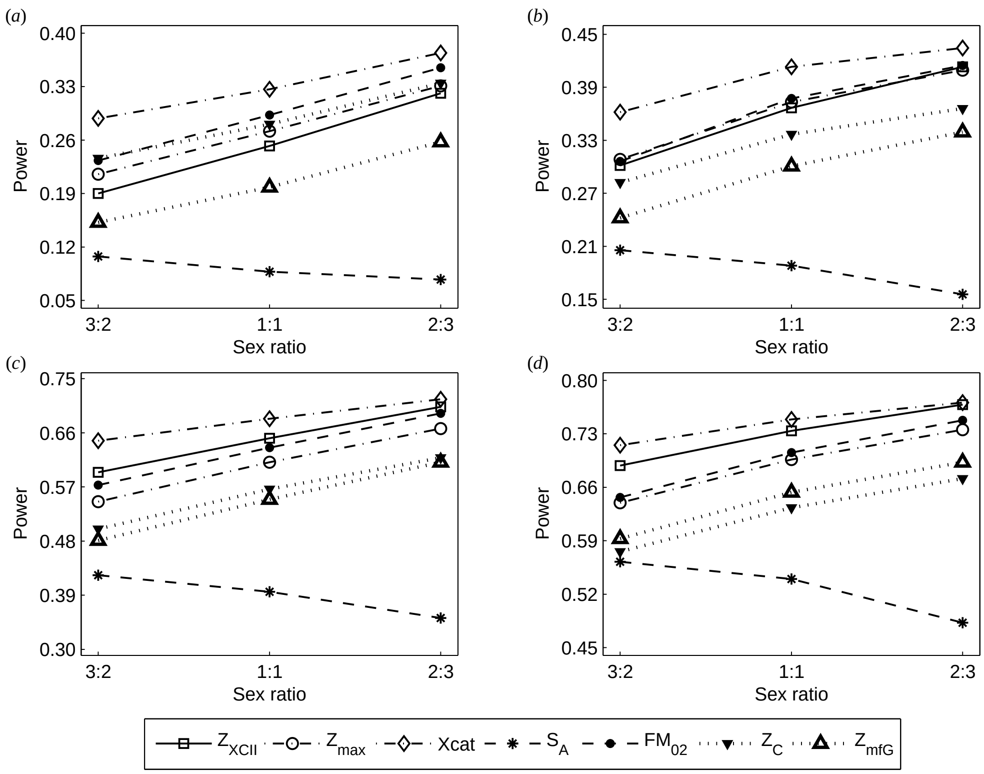

Supplementary Figure S21. Estimated powers of Z_{XCII}, $Z_{\max}$, Xcat, S_A, FM_{02}, Z_C and Z_{mfG} against sex ratio ($r_f : r_m = 3 : 2$, $1 : 1$ and $2 : 3$) under the situation where $p_F = p_M = p$ but HWE does not hold in female offspring when there are XCI with $\gamma = 0$ and no parent-of-origin effects. The simulation is based on 10 000 replicates with $N = 1000$, $\phi_{f0} = \phi_{m0} = \phi_{f01} = \phi_{f10} = 0.120$ and $\phi_{f2} = \phi_{m1} = 0.240$. ($a$) $p = 0.2$, $\rho = -0.05$. (b) $p = 0.2$, $\rho = 0.05$. (c) $p = 0.3$, $\rho = -0.05$. (d) $p = 0.3$, $\rho = 0.05$.

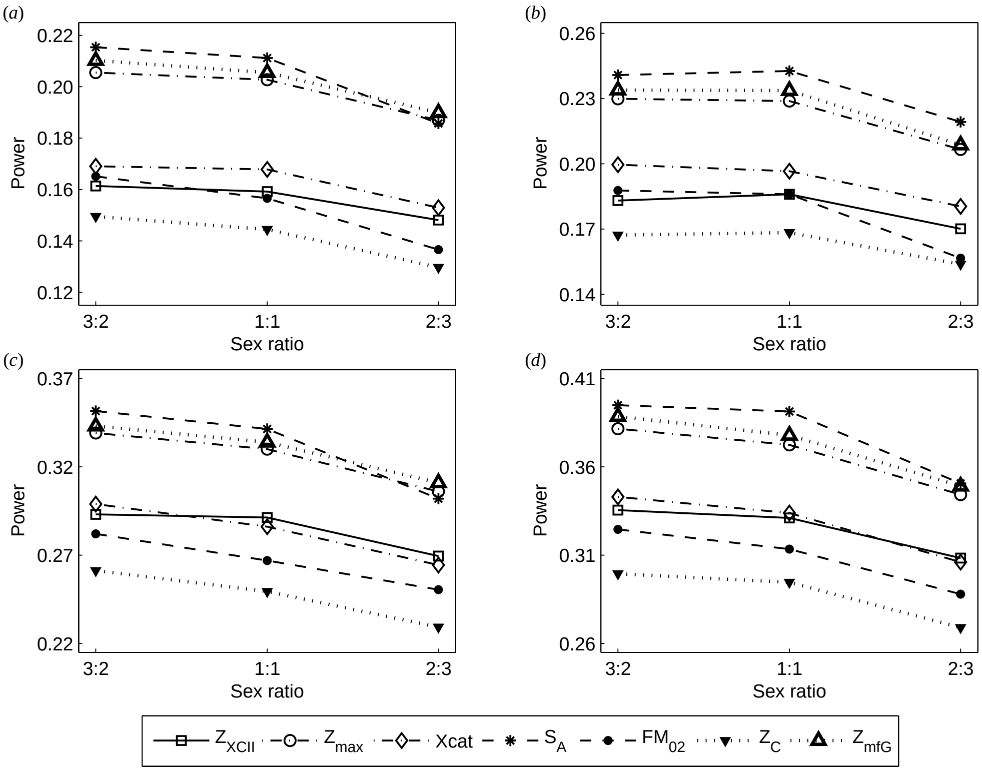

Supplementary Figure S22. Estimated powers of Z_{XCII}, $Z_{\max}$, Xcat, S_A, FM_{02}, Z_C and Z_{mfG} against sex ratio ($r_f : r_m = 3 : 2$, $1 : 1$ and $2 : 3$) under the situation where $p_F = p_M = p$ but HWE does not hold in female offspring when there is neither XCI nor parent-of-origin effects. The simulation is based on 10 000 replicates with $N = 1000$, $\phi_{f0} = \phi_{m0} = 0.120$, $\phi_{f01} = \phi_{f10} = \phi_{m1} = 0.180$ and $\phi_{f2} = 0.240$. (a) $p = 0.2$, $\rho = -0.05$. (b) $p = 0.2$, $\rho = 0.05$. (c) $p = 0.3$, $\rho = -0.05$. (d) $p = 0.3$, $\rho = 0.05$.

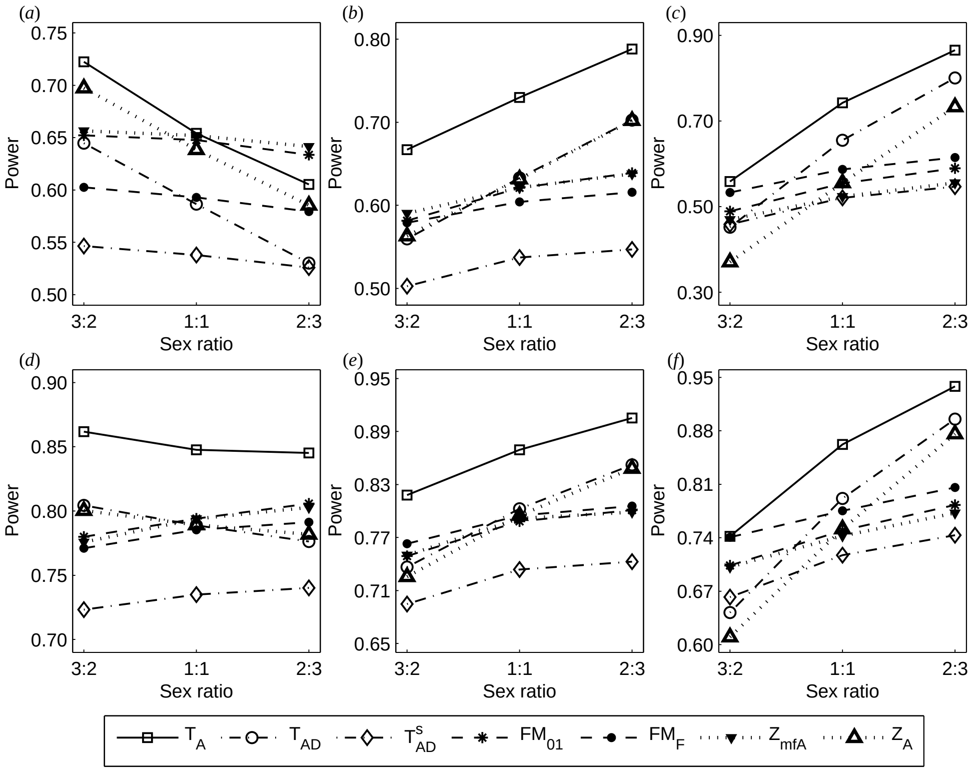

Supplementary Figure S23. Estimated powers of T_A, T_{AD}, T^s_{AD}, FM_{01}, FM_{F}, Z_{mfA} and Z_A against sex ratio ($r_f : r_m = 3:2$, $1:1$ and $2:3$) under random mating when there are XCI with $\gamma = 1$ and complete maternal parent-of-origin effect. The simulation is based on 10 000 replicates with $N = 1000$, $\phi_{f0} = \phi_{m0} = \phi_{f01} = 0.120$ and $\phi_{f10} = \phi_{f2} = \phi_{m1} = 0.240$. ($a$) $p_F = 0.15$, $p_M = 0.25$. (b) $p_F = 0.20$, $p_M = 0.20$. (c) $p_F = 0.25$, $p_M = 0.15$. (d) $p_F = 0.25$, $p_M = 0.35$. (e) $p_F = 0.30$, $p_M = 0.30$. (f) $p_F = 0.35$, $p_M = 0.25$.

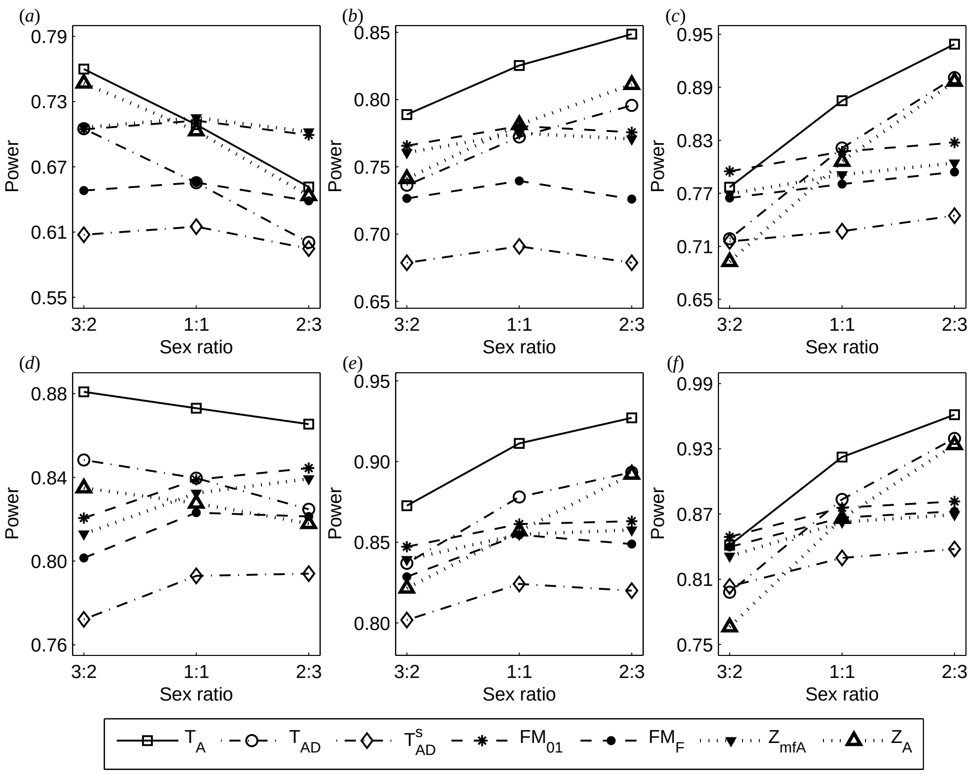

Supplementary Figure S24. Estimated powers of T_A, T_{AD}, T_{AD}^s, FM_{01}, FM_{F}, Z_{mfA} and Z_A against sex ratio ($r_f : r_m = 3 : 2$, $1 : 1$ and $2 : 3$) under random mating when there are XCI with $\gamma = 1.499$ and incomplete maternal parent-of-origin effect. The simulation is based on 10 000 replicates with $N = 1000$, $\phi_{f0} = \phi_{m0} = 0.120$, $\phi_{f01} = 0.192$, $\phi_{f10} = 0.216$ and $\phi_{f2} = \phi_{m1} = 0.240$. ($a$) $p_F = 0.15$, $p_M = 0.25$. (b) $p_F = 0.20$, $p_M = 0.20$. (c) $p_F = 0.25$, $p_M = 0.15$. (d) $p_F = 0.25$, $p_M = 0.35$. (e) $p_F = 0.30$, $p_M = 0.30$. (f) $p_F = 0.35$, $p_M = 0.25$.

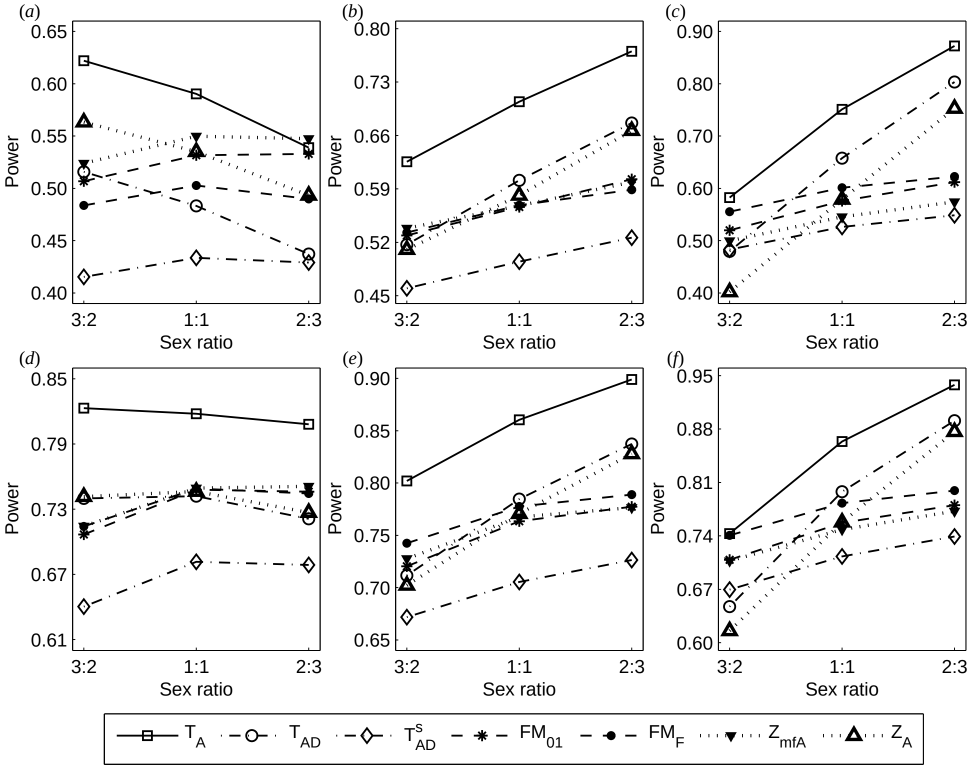

Supplementary Figure S25. Estimated powers of T_A, T_{AD}, T^s_{AD}, FM_{01}, FM_{F}, Z_{mfA} and Z_A against sex ratio ($r_f : r_m = 3 : 2$, $1 : 1$ and $2 : 3$) under random mating when there are XCI with $\gamma = 1.001$ and incomplete maternal parent-of-origin effect. The simulation is based on 10 000 replicates with $N = 1000$, $\phi_{f0} = \phi_{m0} = 0.120$, $\phi_{f01} = 0.144$, $\phi_{f10} = 0.204$ and $\phi_{f2} = \phi_{m1} = 0.240$. ($a$) $p_F = 0.15$, $p_M = 0.25$. (b) $p_F = 0.20$, $p_M = 0.20$. (c) $p_F = 0.25$, $p_M = 0.15$. (d) $p_F = 0.25$, $p_M = 0.35$. (e) $p_F = 0.30$, $p_M = 0.30$. (f) $p_F = 0.35$, $p_M = 0.25$.

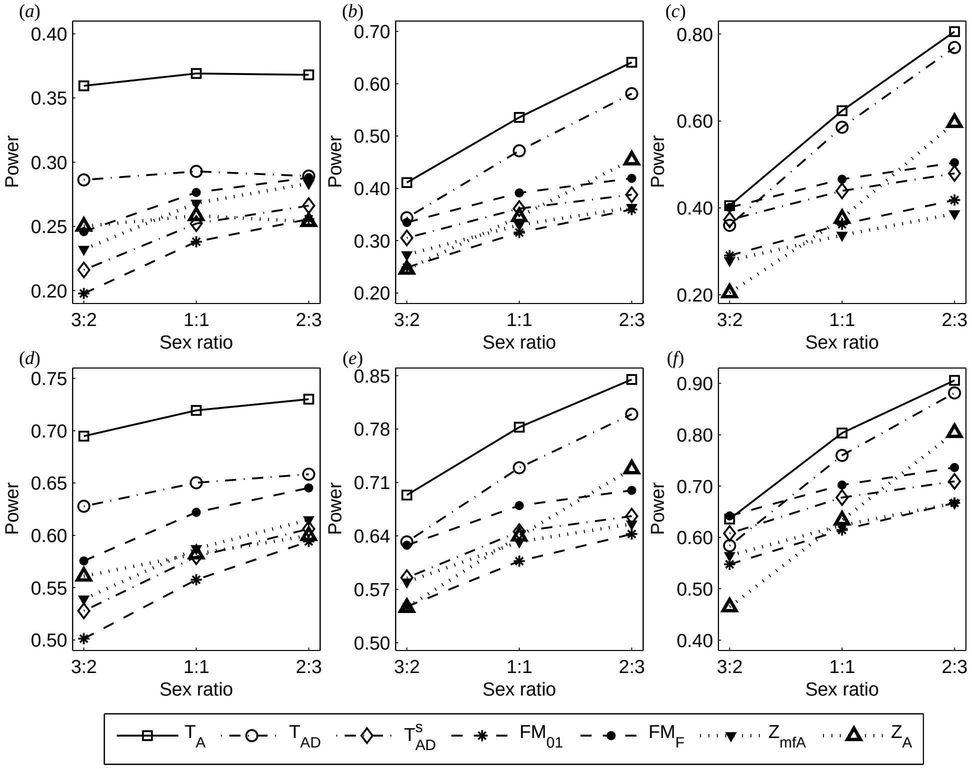

Supplementary Figure S26. Estimated powers of T_A, T_{AD}, T_{AD}^s, FM_{01}, FM_{F}, Z_{mfA} and Z_A against sex ratio ($r_f : r_m = 3 : 2$, $1 : 1$ and $2 : 3$) under random mating when there are XCI with $\gamma = 0.492$ and incomplete maternal parent-of-origin effect. The simulation is based on 10 000 replicates with $N = 1000$, $\phi_{f0} = \phi_{m0} = 0.120$, $\phi_{f01} = 0.132$, $\phi_{f10} = 0.156$ and $\phi_{f2} = \phi_{m1} = 0.240$. (*a*) $p_F = 0.15$, $p_M = 0.25$. (*b*) $p_F = 0.20$, $p_M = 0.20$. (*c*) $p_F = 0.25$, $p_M = 0.15$. (*d*) $p_F = 0.25$, $p_M = 0.35$. (*e*) $p_F = 0.30$, $p_M = 0.30$. (*f*) $p_F = 0.35$, $p_M = 0.25$.

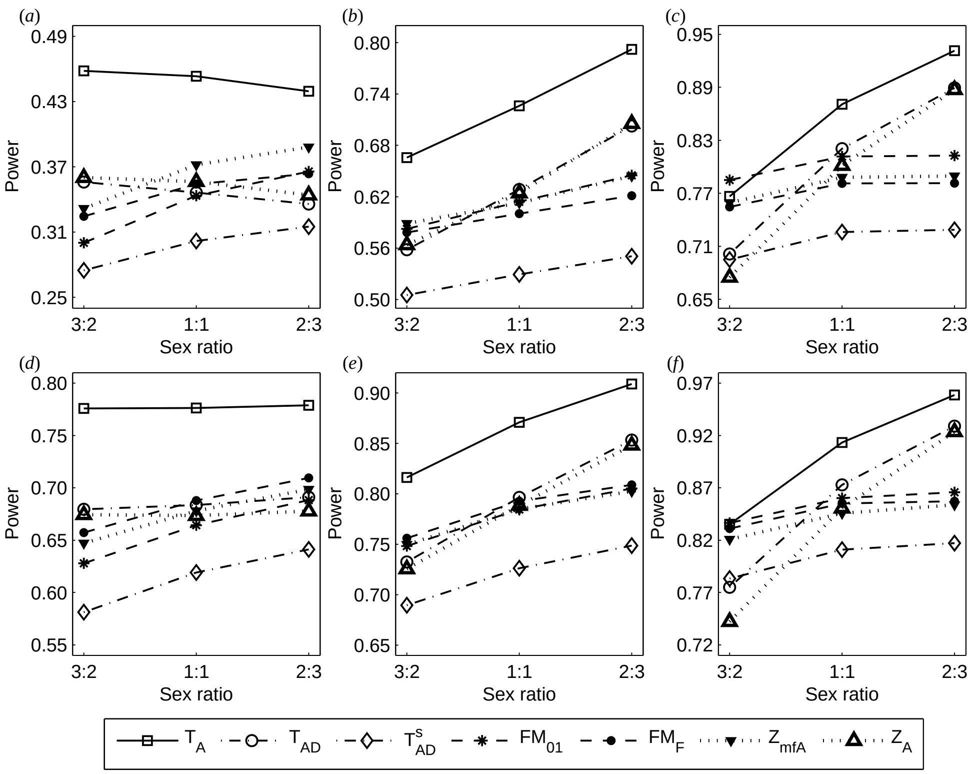

Supplementary Figure S27. Estimated powers of T_A, T_{AD}, T_{AD}^s, FM_{01}, FM_{F}, Z_{mfA} and Z_A against sex ratio ($r_f : r_m = 3 : 2$, $1 : 1$ and $2 : 3$) under random mating when there are XCI with $\gamma = 1$ and complete paternal parent-of-origin effect. The simulation is based on 10 000 replicates with $N = 1000$, $\phi_{f0} = \phi_{m0} = \phi_{f10} = 0.120$ and $\phi_{f01} = \phi_{f2} = \phi_{m1} = 0.240$. (*a*) $p_F = 0.15$, $p_M = 0.25$. (*b*) $p_F = 0.20$, $p_M = 0.20$. (*c*) $p_F = 0.25$, $p_M = 0.15$. (*d*) $p_F = 0.25$, $p_M = 0.35$. (*e*) $p_F = 0.30$, $p_M = 0.30$. (*f*) $p_F = 0.35$, $p_M = 0.25$.

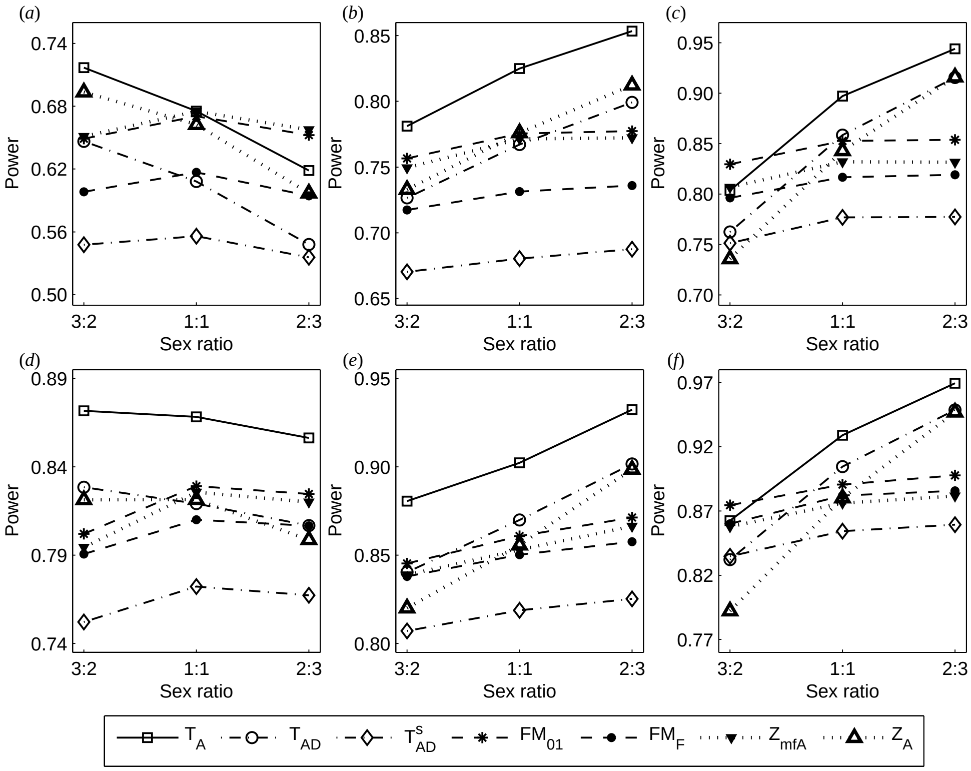

Supplementary Figure S28. Estimated powers of T_A, T_{AD}, T_{AD}^s, FM_{01}, FM_F, Z_{mfA} and Z_A against sex ratio ($r_f : r_m = 3 : 2$, $1 : 1$ and $2 : 3$) under random mating when there are XCI with $\gamma = 1.499$ and incomplete paternal parent-of-origin effect. The simulation is based on 10 000 replicates with $N = 1000$, $\phi_{f0} = \phi_{m0} = 0.120$, $\phi_{f01} = 0.216$, $\phi_{f10} = 0.192$ and $\phi_{f2} = \phi_{m1} = 0.240$. (*a*) $p_F = 0.15$, $p_M = 0.25$. (*b*) $p_F = 0.20$, $p_M = 0.20$. (*c*) $p_F = 0.25$, $p_M = 0.15$. (*d*) $p_F = 0.25$, $p_M = 0.35$. (*e*) $p_F = 0.30$, $p_M = 0.30$. (*f*) $p_F = 0.35$, $p_M = 0.25$.

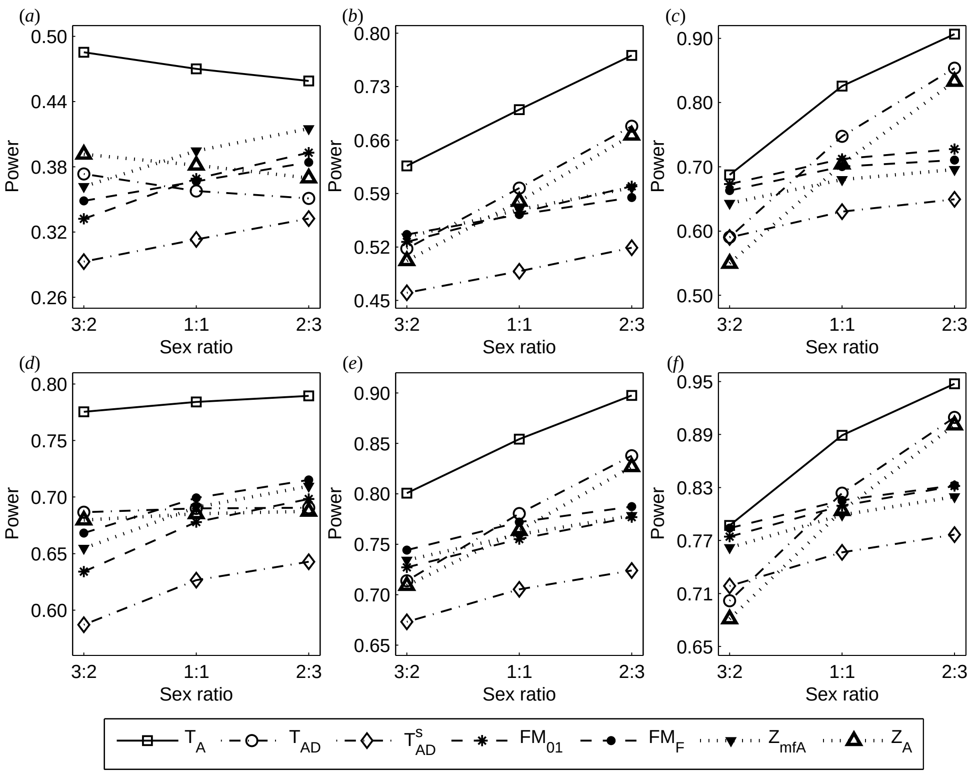

Supplementary Figure S29. Estimated powers of T_A, T_{AD}, T^s_{AD}, FM_{01}, FM_{F}, Z_{mfA} and Z_A against sex ratio ($r_f : r_m = 3 : 2$, $1 : 1$ and $2 : 3$) under random mating when there are XCI with $\gamma = 1.001$ and incomplete paternal parent-of-origin effect. The simulation is based on 10 000 replicates with $N = 1000$, $\phi_{f0} = \phi_{m0} = 0.120$, $\phi_{f01} = 0.204$, $\phi_{f10} = 0.144$ and $\phi_{f2} = \phi_{m1} = 0.240$. (a) $p_F = 0.15$, $p_M = 0.25$. (b) $p_F = 0.20$, $p_M = 0.20$. (c) $p_F = 0.25$, $p_M = 0.15$. (d) $p_F = 0.25$, $p_M = 0.35$. (e) $p_F = 0.30$, $p_M = 0.30$. (f) $p_F = 0.35$, $p_M = 0.25$.

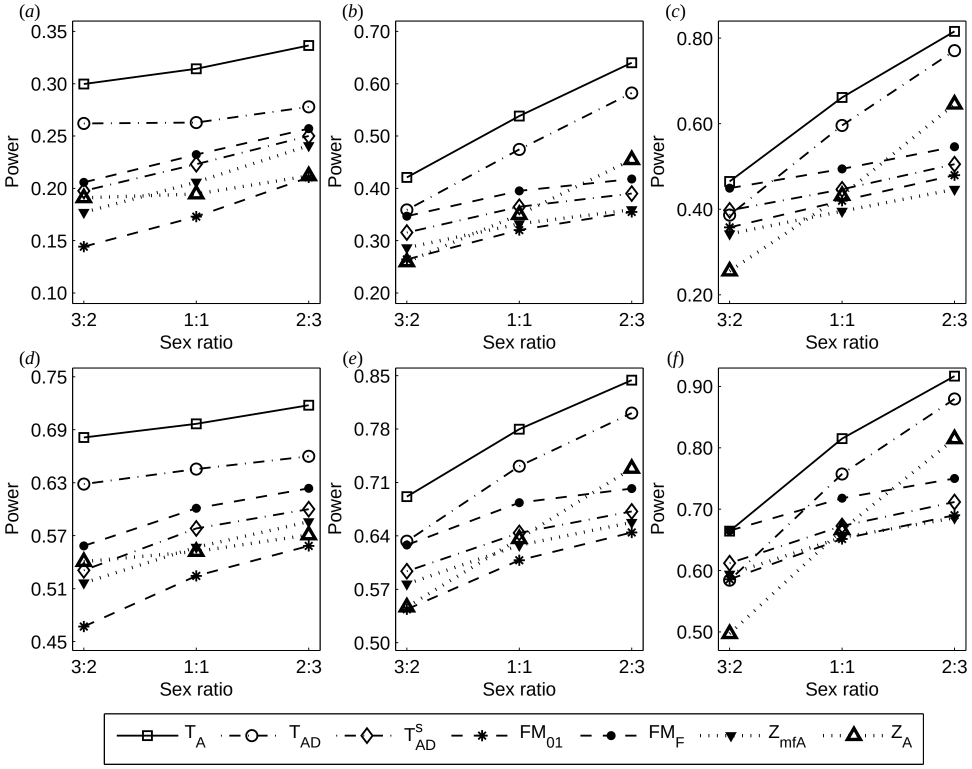

Supplementary Figure S30. Estimated powers of T_A, T_{AD}, T^s_{AD}, FM_{01}, FM_{F}, Z_{mfA} and Z_A against sex ratio ($r_f : r_m = 3:2$, $1:1$ and $2:3$) under random mating when there are XCI with $\gamma = 0.492$ and incomplete paternal parent-of-origin effect. The simulation is based on 10 000 replicates with $N = 1000$, $\phi_{f0} = \phi_{m0} = 0.120$, $\phi_{f01} = 0.156$, $\phi_{f10} = 0.132$ and $\phi_{f2} = \phi_{m1} = 0.240$. (*a*) $p_F = 0.15$, $p_M = 0.25$. (*b*) $p_F = 0.20$, $p_M = 0.20$. (*c*) $p_F = 0.25$, $p_M = 0.15$. (*d*) $p_F = 0.25$, $p_M = 0.35$. (*e*) $p_F = 0.30$, $p_M = 0.30$. (*f*) $p_F = 0.35$, $p_M = 0.25$.

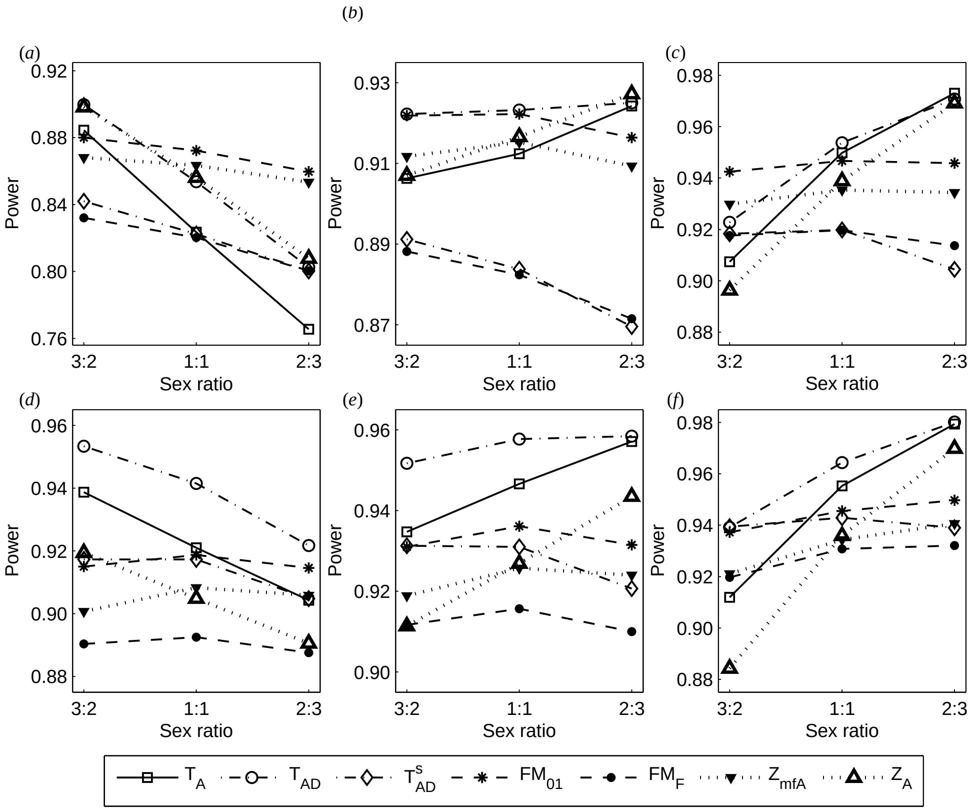

Supplementary Figure S31. Estimated powers of T_A, T_{AD}, T_{AD}^s, FM_{01}, FM_{F}, Z_{mfA} and Z_A against sex ratio ($r_f : r_m = 3:2$, $1:1$ and $2:3$) under random mating when there are XCI with $\gamma = 2$ and no parent-of-origin effects. The simulation is based on 10 000 replicates with $N = 1000$, $\phi_{f0} = \phi_{m0} = 0.120$ and $\phi_{f01} = \phi_{f10} = \phi_{f2} = \phi_{m1} = 0.240$. ($a$) $p_F = 0.15$, $p_M = 0.25$. (b) $p_F = 0.20$, $p_M = 0.20$. (c) $p_F = 0.25$, $p_M = 0.15$. (d) $p_F = 0.25$, $p_M = 0.35$. (e) $p_F = 0.30$, $p_M = 0.30$. (f) $p_F = 0.35$, $p_M = 0.25$.

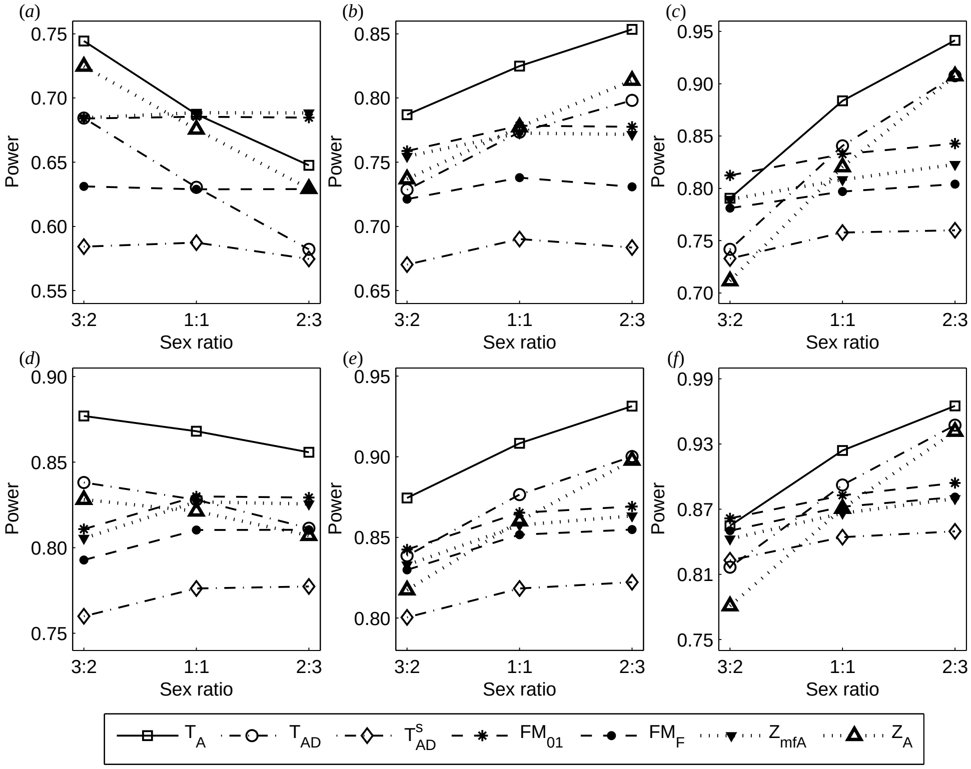

Supplementary Figure S32. Estimated powers of T_A, T_{AD}, T^s_{AD}, FM_{01}, FM_{F}, Z_{mfA} and Z_A against sex ratio ($r_f : r_m = 3:2$, $1:1$ and $2:3$) under random mating when there are XCI with $\gamma = 1.503$ and no parent-of-origin effects. The simulation is based on 10 000 replicates with $N = 1000$, $\phi_{f0} = \phi_{m0} = 0.120$, $\phi_{f01} = \phi_{f10} = 0.204$ and $\phi_{f2} = \phi_{m1} = 0.240$. (a) $p_F = 0.15$, $p_M = 0.25$. (b) $p_F = 0.20$, $p_M = 0.20$. (c) $p_F = 0.25$, $p_M = 0.15$. (d) $p_F = 0.25$, $p_M = 0.35$. (e) $p_F = 0.30$, $p_M = 0.30$. (f) $p_F = 0.35$, $p_M = 0.25$.

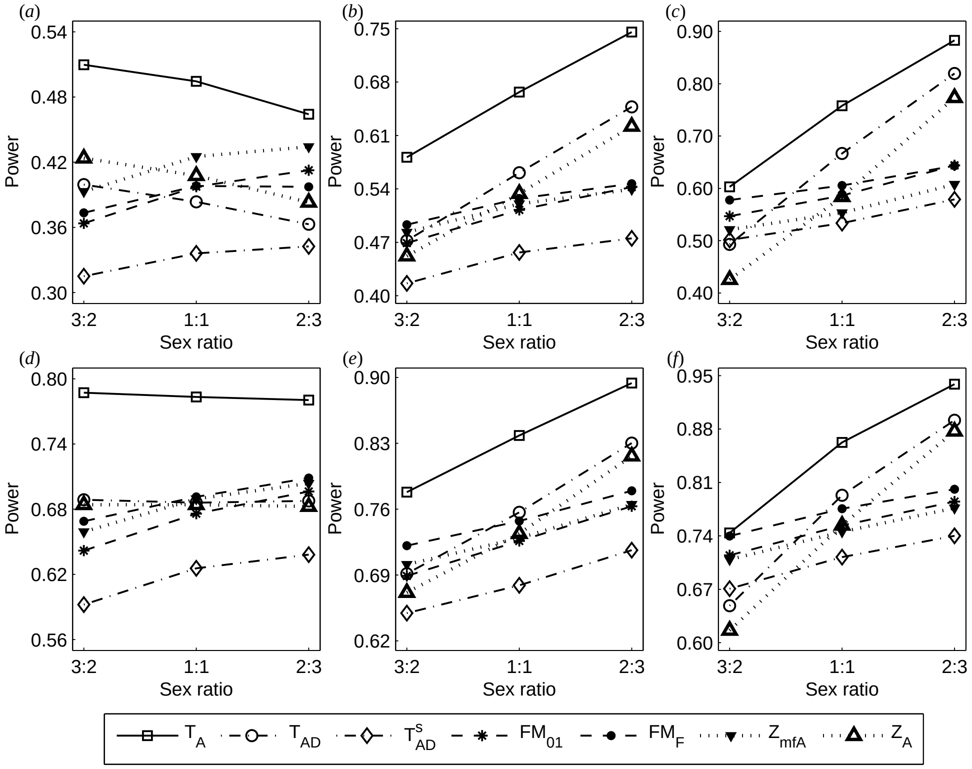

Supplementary Figure S33. Estimated powers of T_A, T_{AD}, T^s_{AD}, FM_{01}, FM_{F}, Z_{mfA} and Z_A against sex ratio ($r_f : r_m = 3 : 2$, $1 : 1$ and $2 : 3$) under random mating when there are XCI with $\gamma = 0.935$ and no parent-of-origin effects. The simulation is based on 10 000 replicates with $N = 1000$, $\phi_{f0} = \phi_{m0} = 0.120$, $\phi_{f01} = \phi_{f10} = 0.168$ and $\phi_{f2} = \phi_{m1} = 0.240$. ($a$) $p_F = 0.15$, $p_M = 0.25$. (b) $p_F = 0.20$, $p_M = 0.20$. (c) $p_F = 0.25$, $p_M = 0.15$. (d) $p_F = 0.25$, $p_M = 0.35$. (e) $p_F = 0.30$, $p_M = 0.30$. (f) $p_F = 0.35$, $p_M = 0.25$.

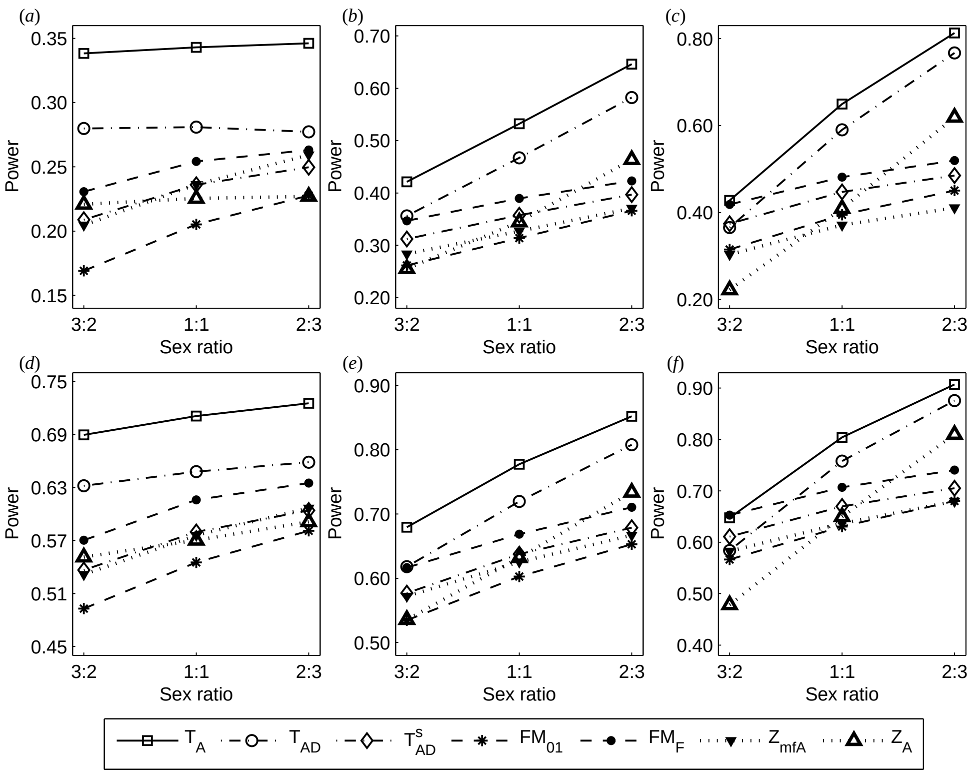

Supplementary Figure S34. Estimated powers of T_A, T_{AD}, T^s_{AD}, FM_{01}, FM_{F}, Z_{mfA} and Z_A against sex ratio ($r_f : r_m = 3:2$, $1:1$ and $2:3$) under random mating when there are XCI with $\gamma = 0.500$ and no parent-of-origin effects. The simulation is based on 10 000 replicates with $N = 1000$, $\phi_{f0} = \phi_{m0} = 0.120$, $\phi_{f01} = \phi_{f10} = 0.144$ and $\phi_{f2} = \phi_{m1} = 0.240$. ($a$) $p_F = 0.15$, $p_M = 0.25$. (b) $p_F = 0.20$, $p_M = 0.20$. (c) $p_F = 0.25$, $p_M = 0.15$. (d) $p_F = 0.25$, $p_M = 0.35$. (e) $p_F = 0.30$, $p_M = 0.30$. (f) $p_F = 0.35$, $p_M = 0.25$.

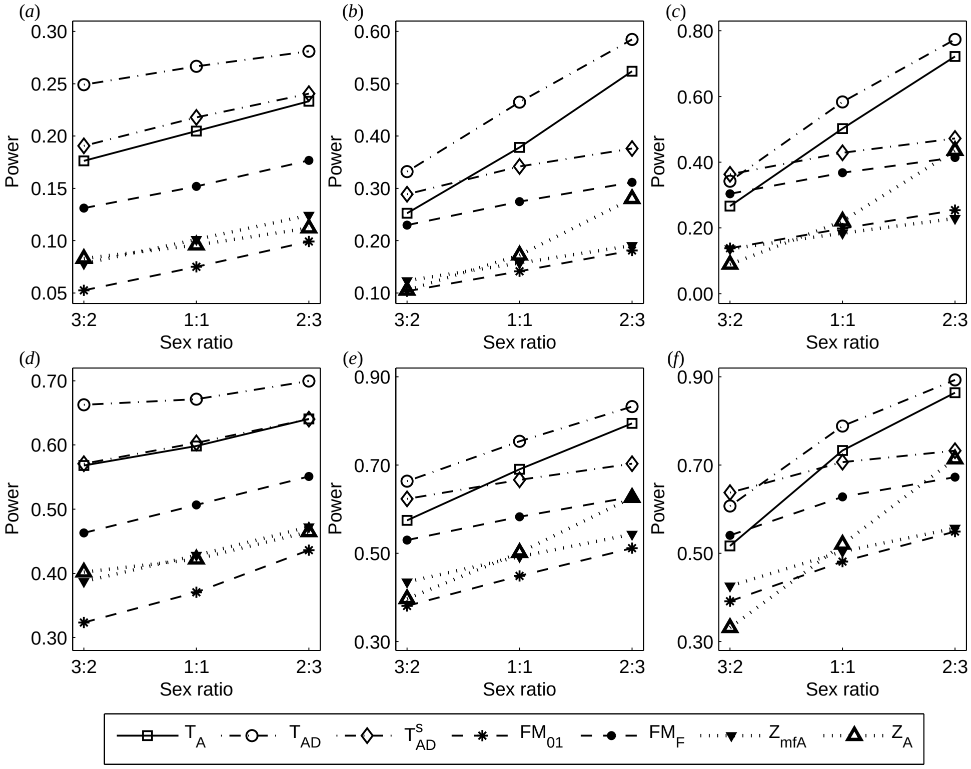

Supplementary Figure S35. Estimated powers of T_A, T_{AD}, T_{AD}^s, FM_{01}, FM_{F}, Z_{mfA} and Z_A against sex ratio ($r_f : r_m = 3 : 2$, $1 : 1$ and $2 : 3$) under random mating when there are XCI with $\gamma = 0$ and no parent-of-origin effects. The simulation is based on 10 000 replicates with $N = 1000$, $\phi_{f0} = \phi_{m0} = \phi_{f01} = \phi_{f10} = 0.120$ and $\phi_{f2} = \phi_{m1} = 0.240$. (a) $p_F = 0.15$, $p_M = 0.25$. (b) $p_F = 0.20$, $p_M = 0.20$. (c) $p_F = 0.25$, $p_M = 0.15$. (d) $p_F = 0.25$, $p_M = 0.35$. (e) $p_F = 0.30$, $p_M = 0.30$. (f) $p_F = 0.35$, $p_M = 0.25$.

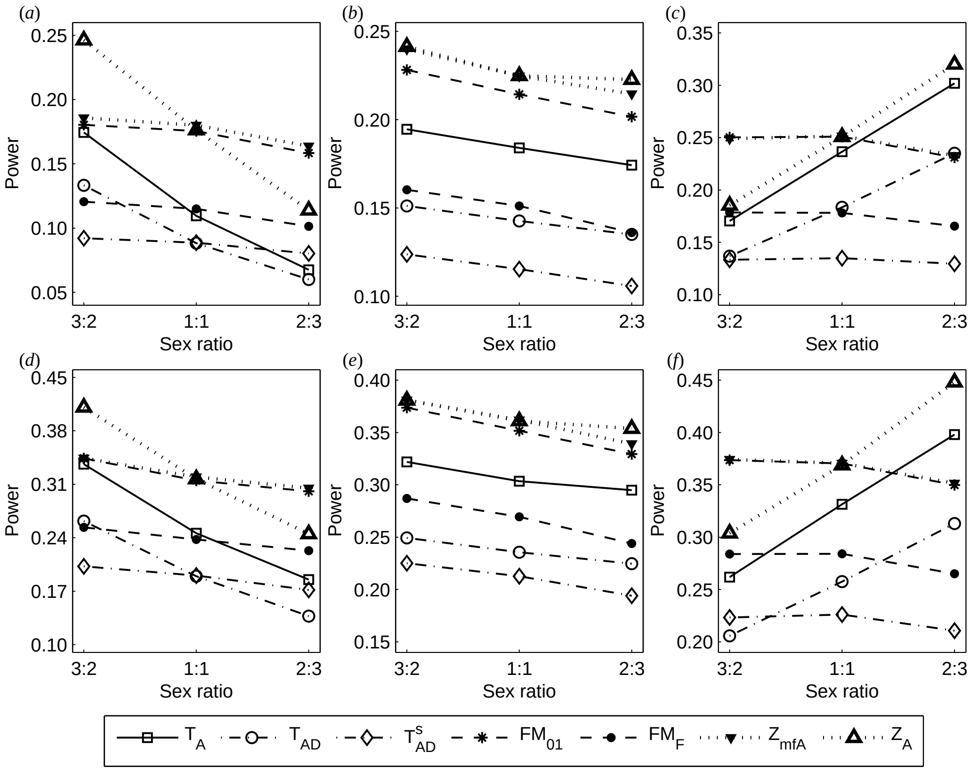

Supplementary Figure S36. Estimated powers of T_A, T_{AD}, T^s_{AD}, FM_{01}, FM_{F}, Z_{mfA} and Z_A against sex ratio ($r_f : r_m = 3 : 2$, $1 : 1$ and $2 : 3$) under random mating when there is neither XCI nor parent-of-origin effects. The simulation is based on 10 000 replicates with $N = 1000$, $\phi_{f0} = \phi_{m0} = 0.120$, $\phi_{f01} = \phi_{f10} = \phi_{m1} = 0.180$ and $\phi_{f2} = 0.240$. (a) $p_F = 0.15$, $p_M = 0.25$. (b) $p_F = 0.20$, $p_M = 0.20$. (c) $p_F = 0.25$, $p_M = 0.15$. (d) $p_F = 0.25$, $p_M = 0.35$. (e) $p_F = 0.30$, $p_M = 0.30$. (f) $p_F = 0.35$, $p_M = 0.25$.

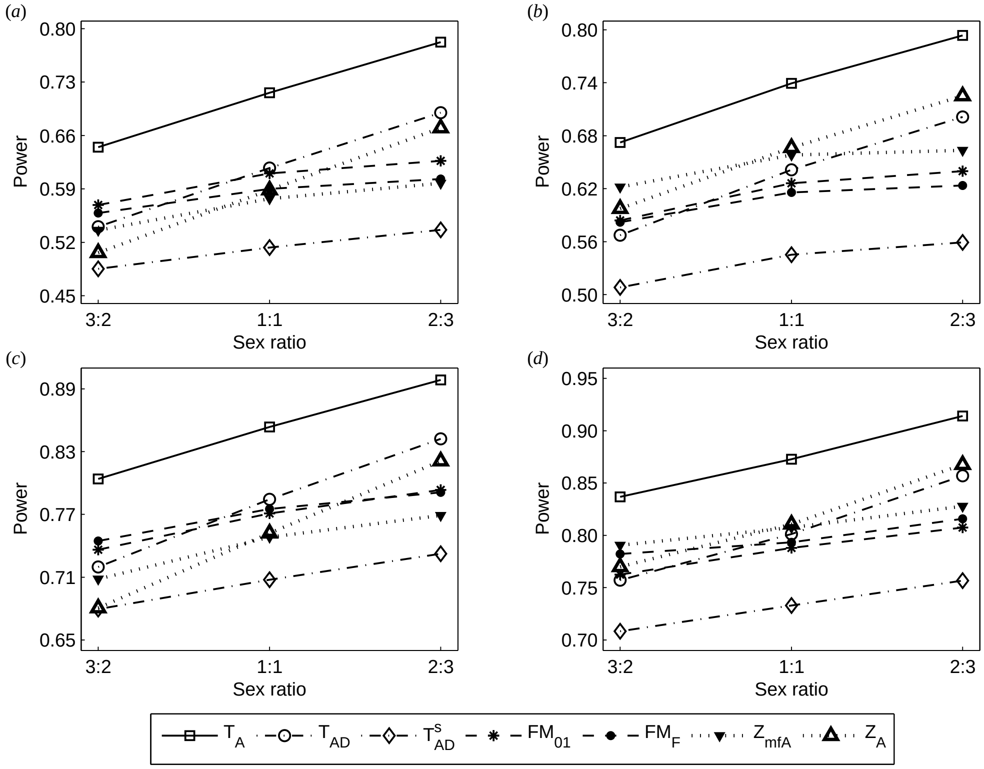

Supplementary Figure S37. Estimated powers of T_A, T_{AD}, T^s_{AD}, FM_{01}, FM_F, Z_{mfA} and Z_A against sex ratio ($r_f : r_m = 3 : 2$, $1 : 1$ and $2 : 3$) under the situation where $p_F = p_M = p$ but HWE does not hold in female offspring when there are XCI with $\gamma = 1$ and complete maternal parent-of-origin effect. The simulation is based on 10 000 replicates with $N = 1000$, $\phi_{f0} = \phi_{m0} = \phi_{f01} = 0.120$ and $\phi_{f10} = \phi_{f2} = \phi_{m1} = 0.240$. ($a$) $p = 0.2$, $\rho = -0.05$. (b) $p = 0.2$, $\rho = 0.05$. (c) $p = 0.3$, $\rho = -0.05$. (d) $p = 0.3$, $\rho = 0.05$.

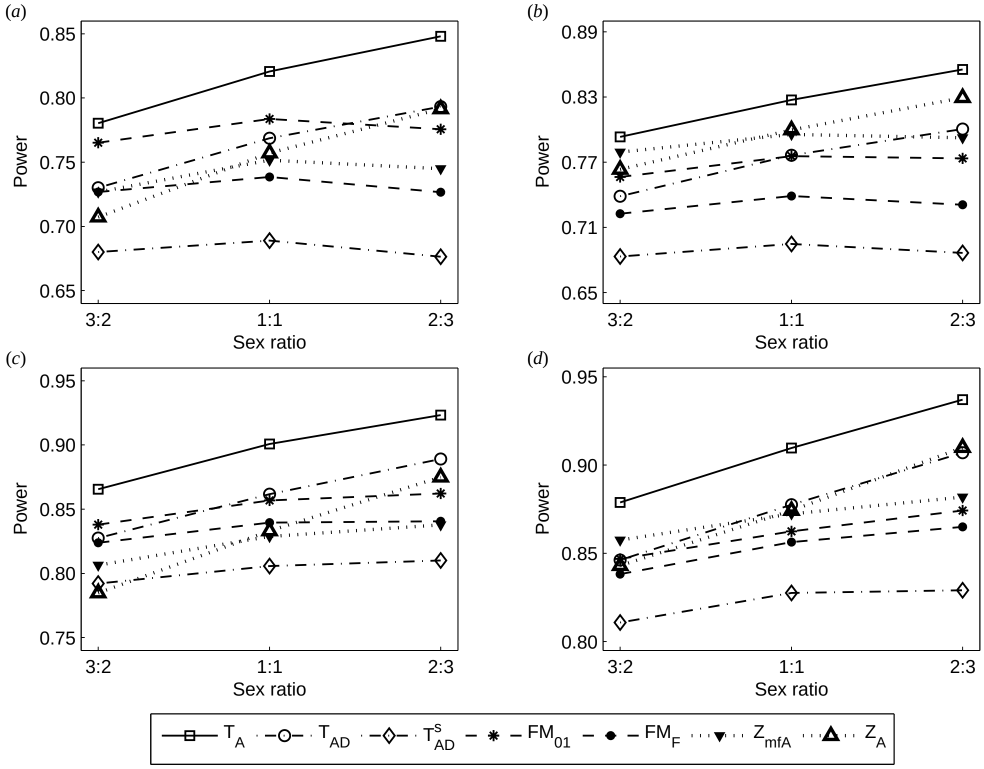

Supplementary Figure S38. Estimated powers of T_A, T_{AD}, T^s_{AD}, FM_{01}, FM_{F}, Z_{mfA} and Z_A against sex ratio ($r_f : r_m = 3 : 2$, $1 : 1$ and $2 : 3$) under the situation where $p_{_F} = p_{_M} = p$ but HWE does not hold in female offspring when there are XCI with $\gamma = 1.499$ and incomplete maternal parent-of-origin effect. The simulation is based on 10 000 replicates with $N = 1000$, $\phi_{f0} = \phi_{m0} = 0.120$, $\phi_{f01} = 0.192$, $\phi_{f10} = 0.216$ and $\phi_{f2} = \phi_{m1} = 0.240$. (*a*) $p = 0.2$, $\rho = -0.05$. (*b*) $p = 0.2$, $\rho = 0.05$. (*c*) $p = 0.3$, $\rho = -0.05$. (*d*) $p = 0.3$, $\rho = 0.05$.

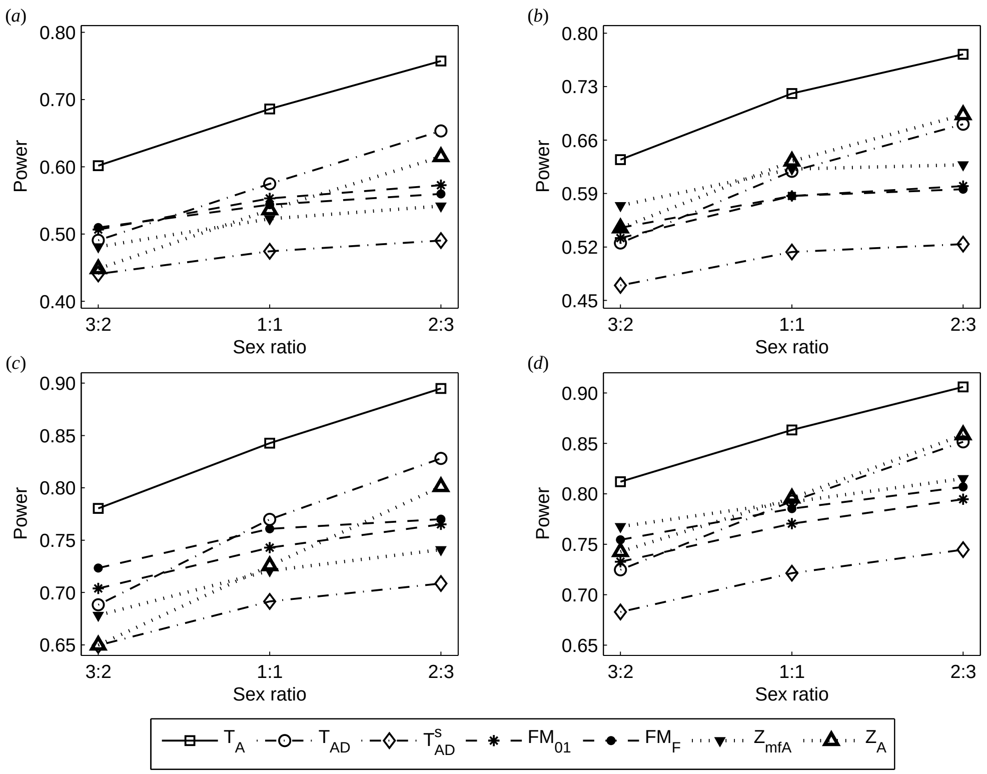

Supplementary Figure S39. Estimated powers of T_A, T_{AD}, T^s_{AD}, FM_{01}, FM_{F}, Z_{mfA} and Z_A against sex ratio ($r_f : r_m = 3:2$, $1:1$ and $2:3$) under the situation where $p_{_F} = p_{_M} = p$ but HWE does not hold in female offspring when there are XCI with $\gamma = 1.001$ and incomplete maternal parent-of-origin effect. The simulation is based on 10 000 replicates with $N = 1000$, $\phi_{f0} = \phi_{m0} = 0.120$, $\phi_{f01} = 0.144$, $\phi_{f10} = 0.204$ and $\phi_{f2} = \phi_{m1} = 0.240$. (a) $p = 0.2$, $\rho = -0.05$. (b) $p = 0.2$, $\rho = 0.05$. (c) $p = 0.3$, $\rho = -0.05$. (d) $p = 0.3$, $\rho = 0.05$.

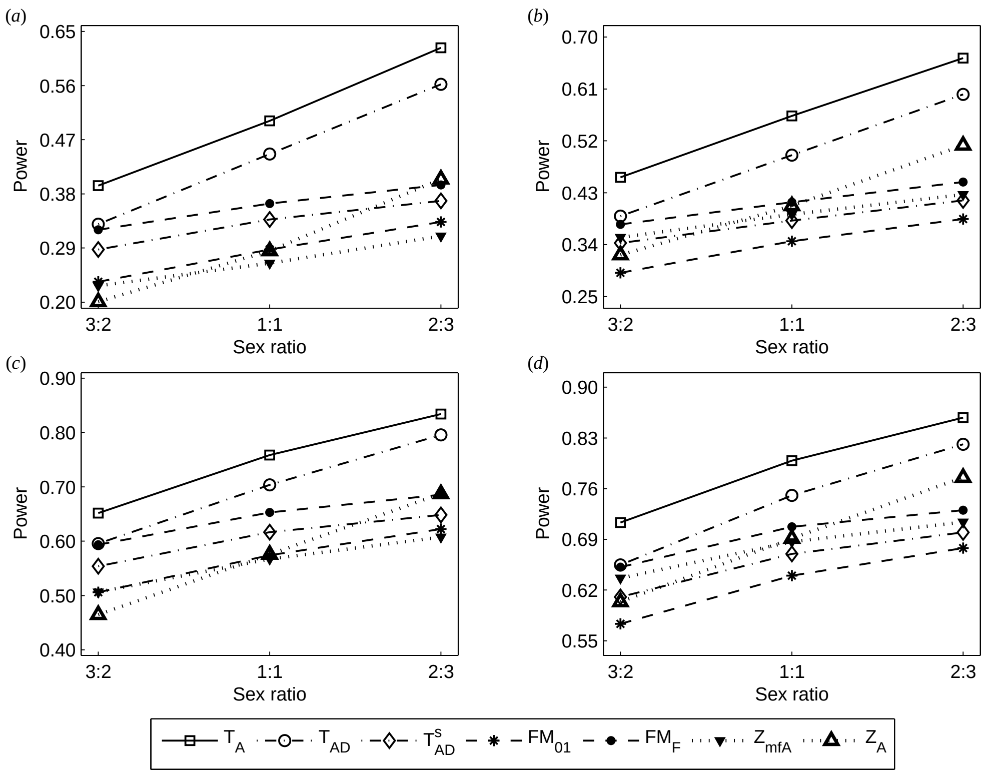

Supplementary Figure S40. Estimated powers of T_A, T_{AD}, T^s_{AD}, FM_{01}, FM_{F}, Z_{mfA} and Z_A against sex ratio ($r_f : r_m = 3 : 2$, $1 : 1$ and $2 : 3$) under the situation where $p_{_F} = p_{_M} = p$ but HWE does not hold in female offspring when there are XCI with $\gamma = 0.492$ and incomplete maternal parent-of-origin effect. The simulation is based on 10 000 replicates with $N = 1000$, $\phi_{f0} = \phi_{m0} = 0.120$, $\phi_{f01} = 0.132$, $\phi_{f10} = 0.156$ and $\phi_{f2} = \phi_{m1} = 0.240$. ($a$) $p = 0.2$, $\rho = -0.05$. (b) $p = 0.2$, $\rho = 0.05$. (c) $p = 0.3$, $\rho = -0.05$. (d) $p = 0.3$, $\rho = 0.05$.

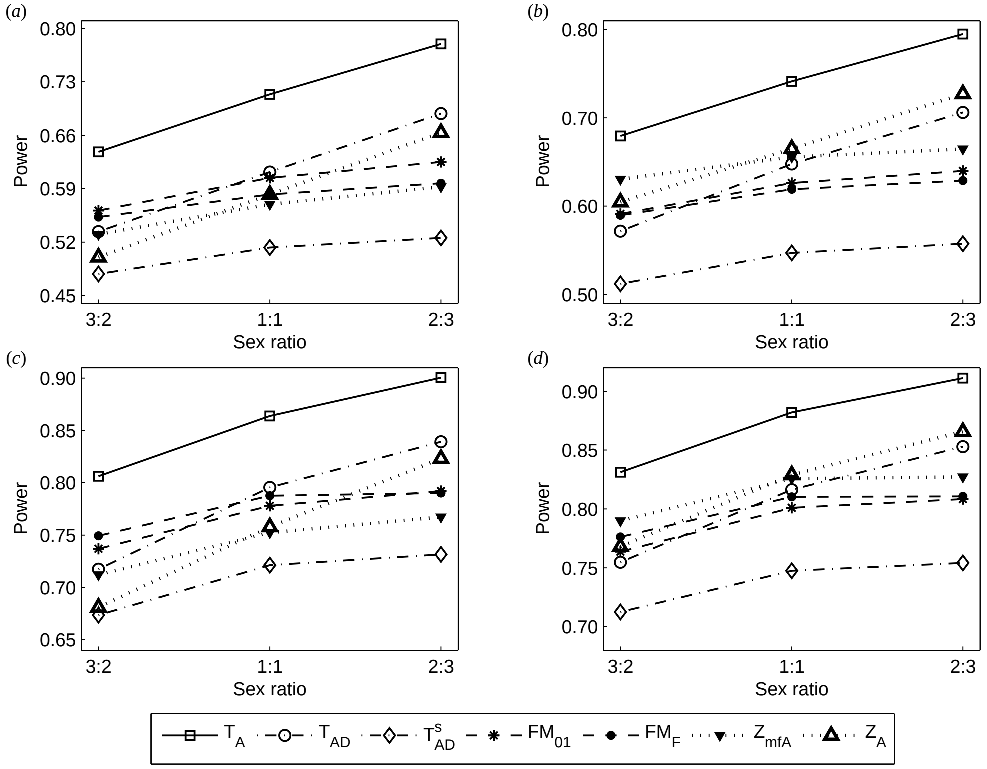

Supplementary Figure S41. Estimated powers of T_A, T_{AD}, T_{AD}^s, FM_{01}, FM_{F}, Z_{mfA} and Z_A against sex ratio ($r_f : r_m = 3 : 2$, $1 : 1$ and $2 : 3$) under the situation where $p_{_F} = p_{_M} = p$ but HWE does not hold in female offspring when there are XCI with $\gamma = 1$ and complete paternal parent-of-origin effect. The simulation is based on 10 000 replicates with $N = 1000$, $\phi_{f0} = \phi_{m0} = \phi_{f10} = 0.120$ and $\phi_{f01} = \phi_{f2} = \phi_{m1} = 0.240$. ($a$) $p = 0.2$, $\rho = -0.05$. (b) $p = 0.2$, $\rho = 0.05$. (c) $p = 0.3$, $\rho = -0.05$. (d) $p = 0.3$, $\rho = 0.05$.

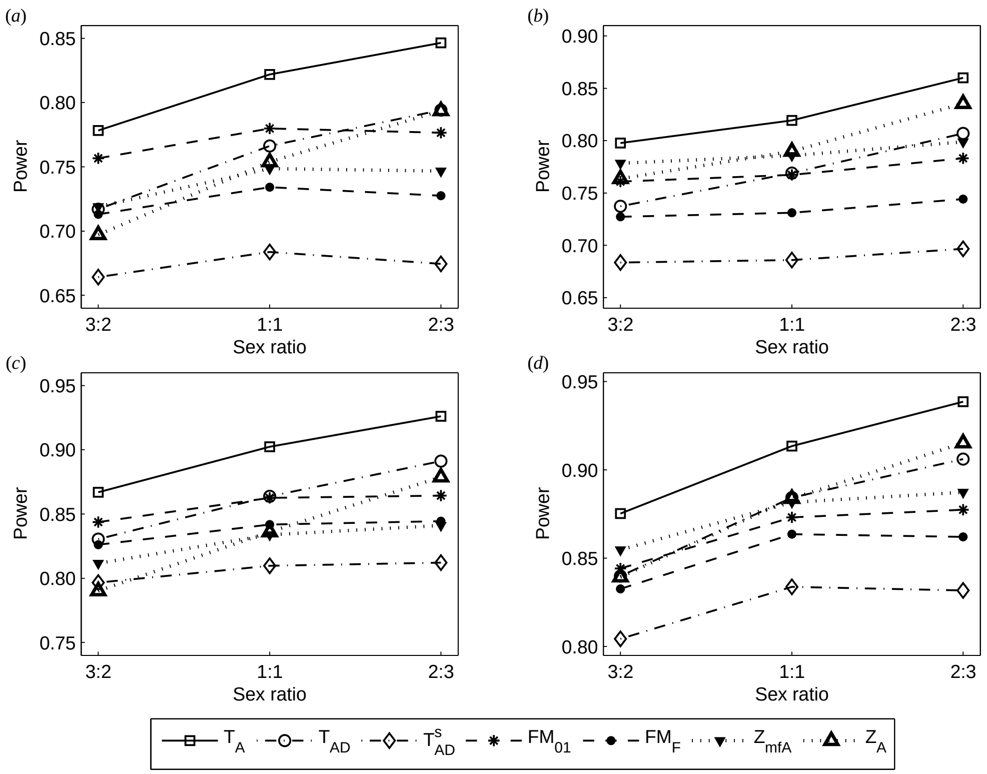

Supplementary Figure S42. Estimated powers of T_A, T_{AD}, T_{AD}^s, FM_{01}, FM_{F}, Z_{mfA} and Z_A against sex ratio ($r_f : r_m = 3:2$, $1:1$ and $2:3$) under the situation where $p_{_F} = p_{_M} = p$ but HWE does not hold in female offspring when there are XCI with $\gamma = 1.499$ and incomplete paternal parent-of-origin effect. The simulation is based on 10 000 replicates with $N = 1000$, $\phi_{f0} = \phi_{m0} = 0.120$, $\phi_{f01} = 0.216$, $\phi_{f10} = 0.192$ and $\phi_{f2} = \phi_{m1} = 0.240$. ($a$) $p = 0.2$, $\rho = -0.05$. (b) $p = 0.2$, $\rho = 0.05$. (c) $p = 0.3$, $\rho = -0.05$. (d) $p = 0.3$, $\rho = 0.05$.

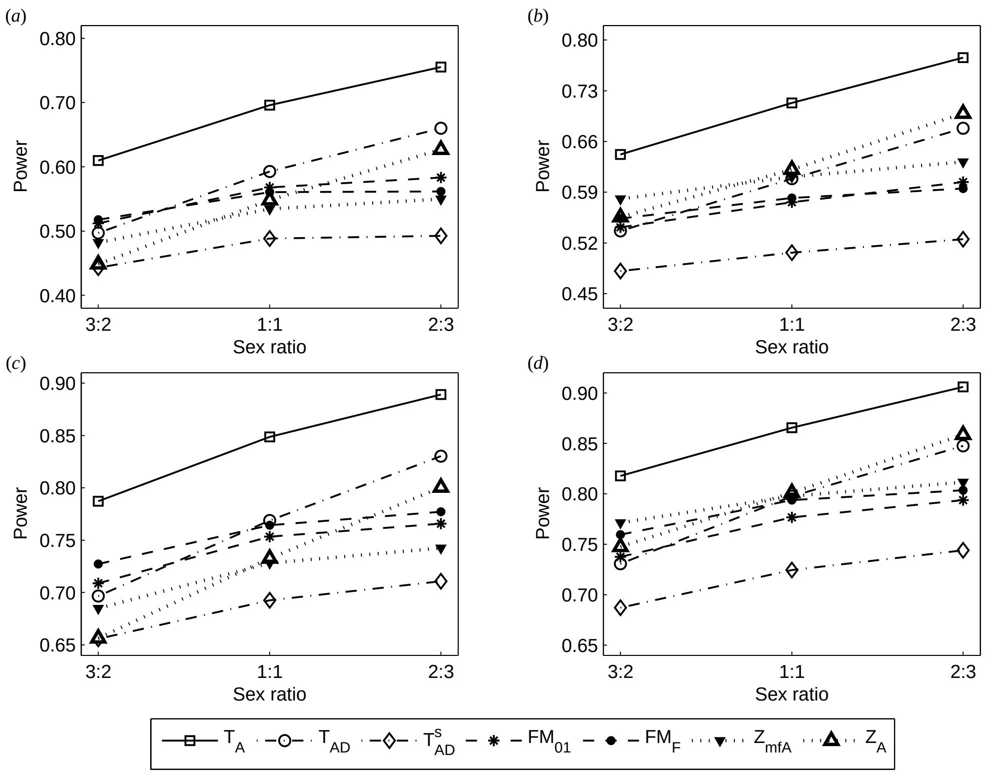

Supplementary Figure S43. Estimated powers of T_A, T_{AD}, T^s_{AD}, FM_{01}, FM_{F}, Z_{mfA} and Z_A against sex ratio ($r_f : r_m = 3:2$, $1:1$ and $2:3$) under the situation where $p_{_F} = p_{_M} = p$ but HWE does not hold in female offspring when there are XCI with $\gamma = 1.001$ and incomplete paternal parent-of-origin effect. The simulation is based on 10 000 replicates with $N = 1000$, $\phi_{f0} = \phi_{m0} = 0.120$, $\phi_{f01} = 0.204$, $\phi_{f10} = 0.144$ and $\phi_{f2} = \phi_{m1} = 0.240$. ($a$) $p = 0.2$, $\rho = -0.05$. (b) $p = 0.2$, $\rho = 0.05$. (c) $p = 0.3$, $\rho = -0.05$. (d) $p = 0.3$, $\rho = 0.05$.

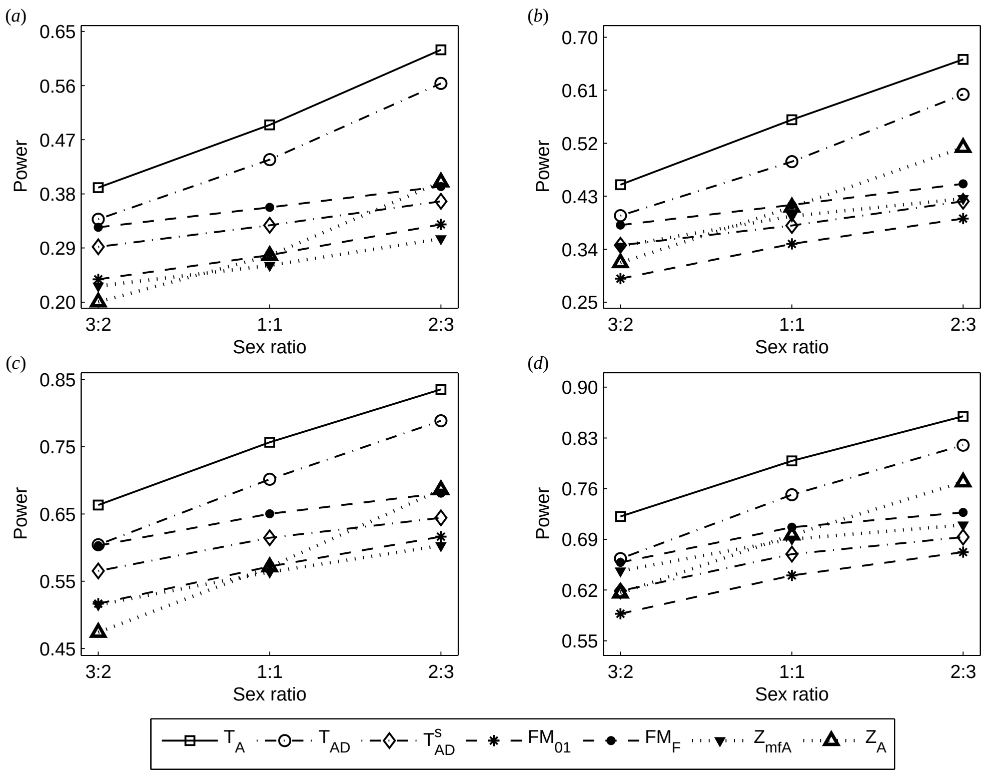

Supplementary Figure S44. Estimated powers of T_A, T_{AD}, T^s_{AD}, FM_{01}, FM_{F}, Z_{mfA} and Z_A against sex ratio ($r_f : r_m = 3 : 2$, $1 : 1$ and $2 : 3$) under the situation where $p_F = p_M = p$ but HWE does not hold in female offspring when there are XCI with $\gamma = 0.492$ and incomplete paternal parent-of-origin effect. The simulation is based on 10 000 replicates with $N = 1000$, $\phi_{f0} = \phi_{m0} = 0.120$, $\phi_{f01} = 0.156$, $\phi_{f10} = 0.132$ and $\phi_{f2} = \phi_{m1} = 0.240$. ($a$) $p = 0.2$, $\rho = -0.05$. (b) $p = 0.2$, $\rho = 0.05$. (c) $p = 0.3$, $\rho = -0.05$. (d) $p = 0.3$, $\rho = 0.05$.

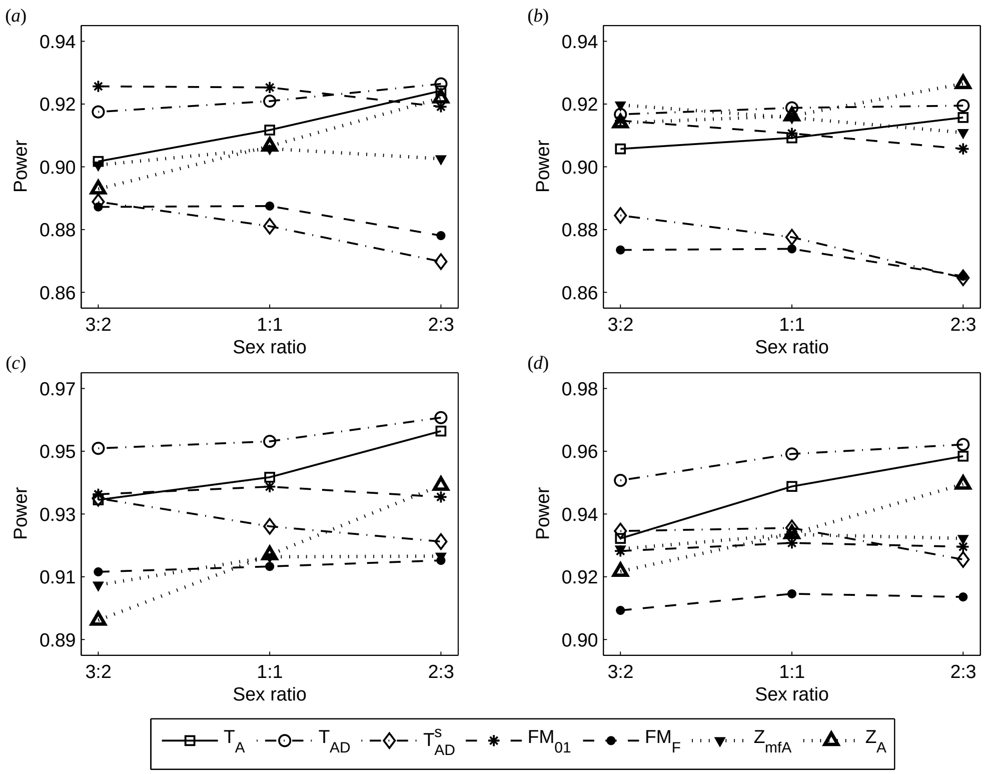

Supplementary Figure S45. Estimated powers of T_A, T_{AD}, T^s_{AD}, FM_{01}, FM_F, Z_{mfA} and Z_A against sex ratio ($r_f : r_m = 3:2$, $1:1$ and $2:3$) under the situation where $p_{_F} = p_{_M} = p$ but HWE does not hold in female offspring when there are XCI with $\gamma = 2$ and no parent-of-origin effects. The simulation is based on 10 000 replicates with $N = 1000$, $\phi_{f0} = \phi_{m0} = 0.120$ and $\phi_{f01} = \phi_{f10} = \phi_{f2} = \phi_{m1} = 0.240$. ($a$) $p = 0.2$, $\rho = -0.05$. (b) $p = 0.2$, $\rho = 0.05$. (c) $p = 0.3$, $\rho = -0.05$. (d) $p = 0.3$, $\rho = 0.05$.

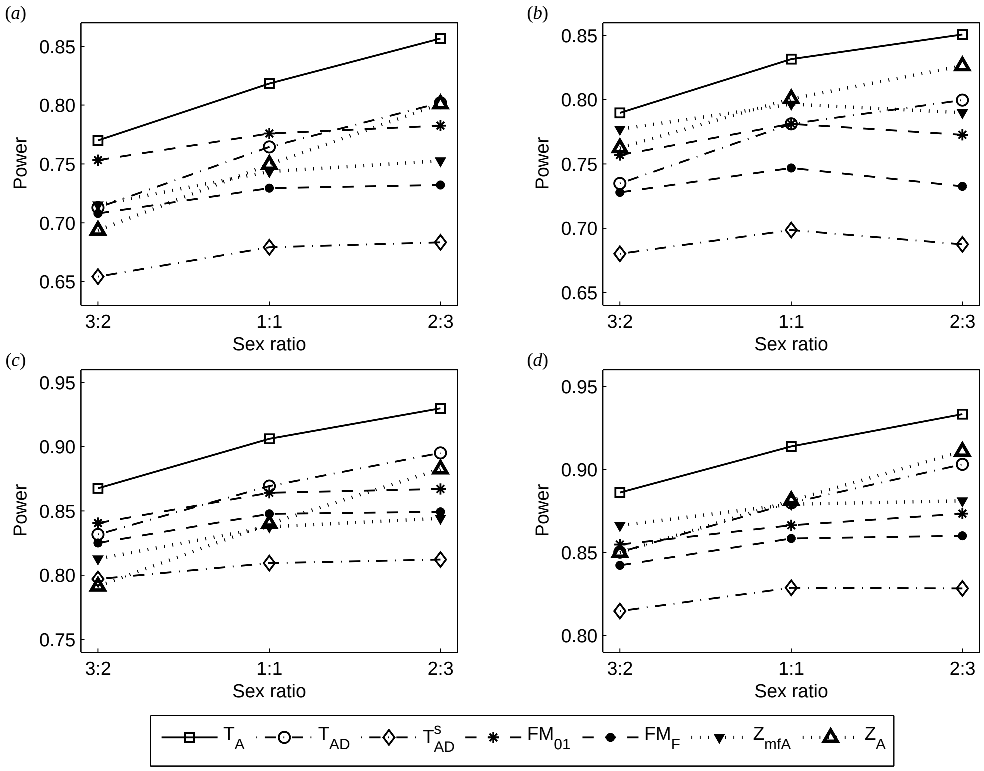

Supplementary Figure S46. Estimated powers of T_A, T_{AD}, T^s_{AD}, FM_{01}, FM_{F}, Z_{mfA} and Z_A against sex ratio ($r_f : r_m = 3:2$, $1:1$ and $2:3$) under the situation where $p_{_F} = p_{_M} = p$ but HWE does not hold in female offspring when there are XCI with $\gamma = 1.503$ and no parent-of-origin effects. The simulation is based on 10 000 replicates with $N = 1000$, $\phi_{f0} = \phi_{m0} = 0.120$, $\phi_{f01} = \phi_{f10} = 0.204$ and $\phi_{f2} = \phi_{m1} = 0.240$. ($a$) $p = 0.2$, $\rho = -0.05$. (b) $p = 0.2$, $\rho = 0.05$. (c) $p = 0.3$, $\rho = -0.05$. (d) $p = 0.3$, $\rho = 0.05$.

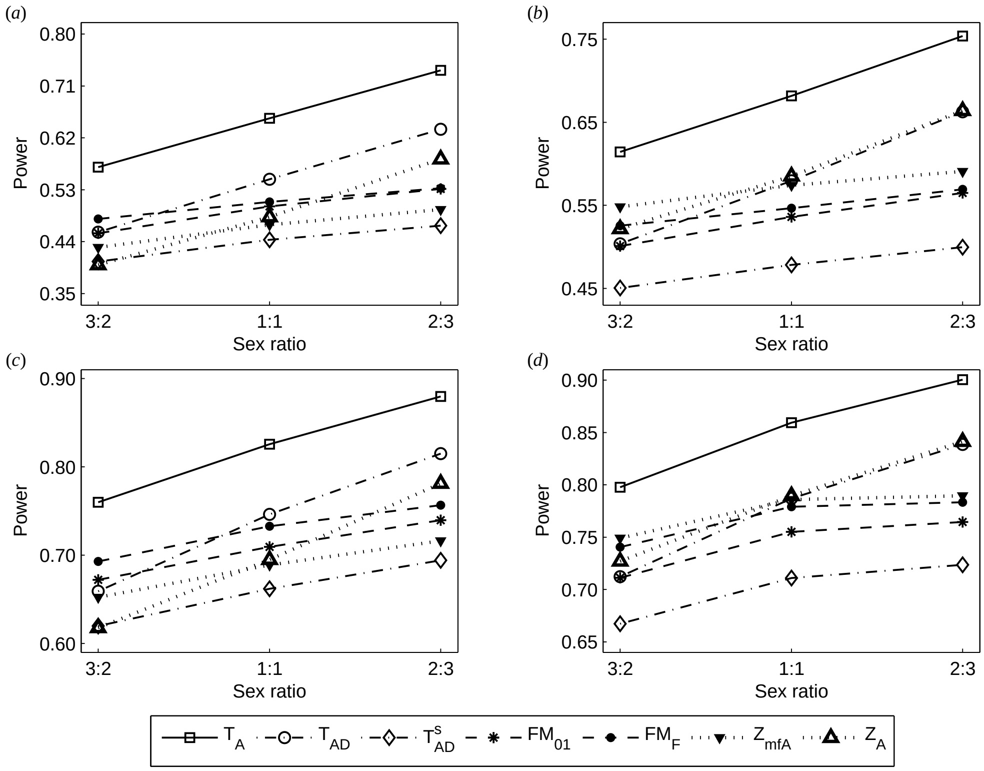

Supplementary Figure S47. Estimated powers of T_A, T_{AD}, T^s_{AD}, FM_{01}, FM_{F}, Z_{mfA} and Z_A against sex ratio ($r_f : r_m = 3:2$, $1:1$ and $2:3$) under the situation where $p_{_F} = p_{_M} = p$ but HWE does not hold in female offspring when there are XCI with $\gamma = 0.935$ and no parent-of-origin effects. The simulation is based on 10 000 replicates with $N = 1000$, $\phi_{f0} = \phi_{m0} = 0.120$, $\phi_{f01} = \phi_{f10} = 0.168$ and $\phi_{f2} = \phi_{m1} = 0.240$. ($a$) $p = 0.2$, $\rho = -0.05$. (b) $p = 0.2$, $\rho = 0.05$. (c) $p = 0.3$, $\rho = -0.05$. (d) $p = 0.3$, $\rho = 0.05$.

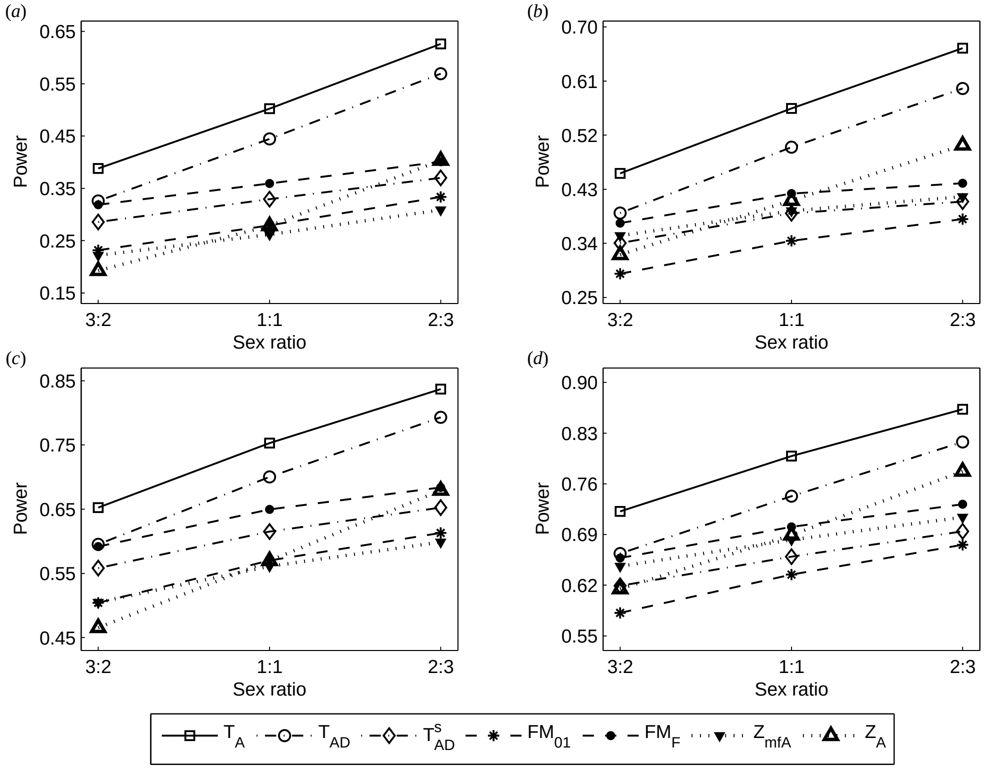

Supplementary Figure S48. Estimated powers of T_A, T_{AD}, T_{AD}^s, FM_{01}, FM_{F}, Z_{mfA} and Z_A against sex ratio ($r_f : r_m = 3:2$, $1:1$ and $2:3$) under the situation where $p_F = p_M = p$ but HWE does not hold in female offspring when there are XCI with $\gamma = 0.500$ and no parent-of-origin effects. The simulation is based on 10 000 replicates with $N = 1000$, $\phi_{f0} = \phi_{m0} = 0.120$, $\phi_{f01} = \phi_{f10} = 0.144$ and $\phi_{f2} = \phi_{m1} = 0.240$. ($a$) $p = 0.2$, $\rho = -0.05$. (b) $p = 0.2$, $\rho = 0.05$. (c) $p = 0.3$, $\rho = -0.05$. (d) $p = 0.3$, $\rho = 0.05$.

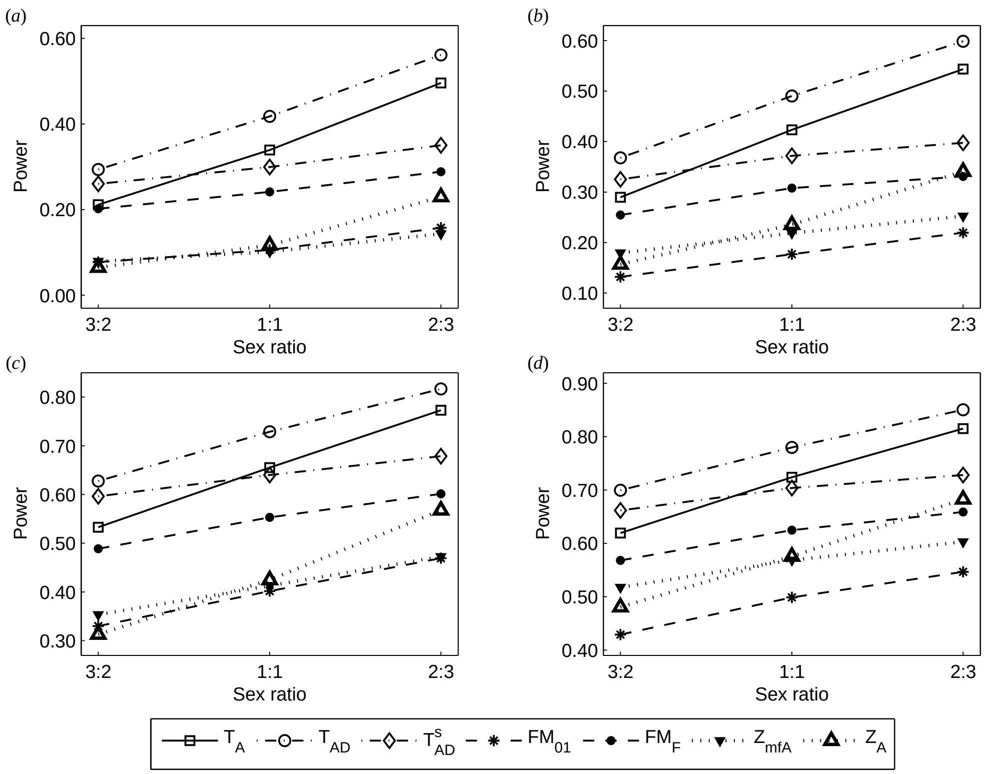

Supplementary Figure S49. Estimated powers of T_A, T_{AD}, T_{AD}^s, FM_{01}, FM_{F}, Z_{mfA} and Z_A against sex ratio ($r_f : r_m = 3:2$, $1:1$ and $2:3$) under the situation where $p_{_F} = p_{_M} = p$ but HWE does not hold in female offspring when there are XCI with $\gamma = 0$ and no parent-of-origin effects. The simulation is based on 10 000 replicates with $N = 1000$, $\phi_{f0} = \phi_{m0} = \phi_{f01} = \phi_{f10} = 0.120$ and $\phi_{f2} = \phi_{m1} = 0.240$. ($a$) $p = 0.2$, $\rho = -0.05$. (b) $p = 0.2$, $\rho = 0.05$. (c) $p = 0.3$, $\rho = -0.05$. (d) $p = 0.3$, $\rho = 0.05$.

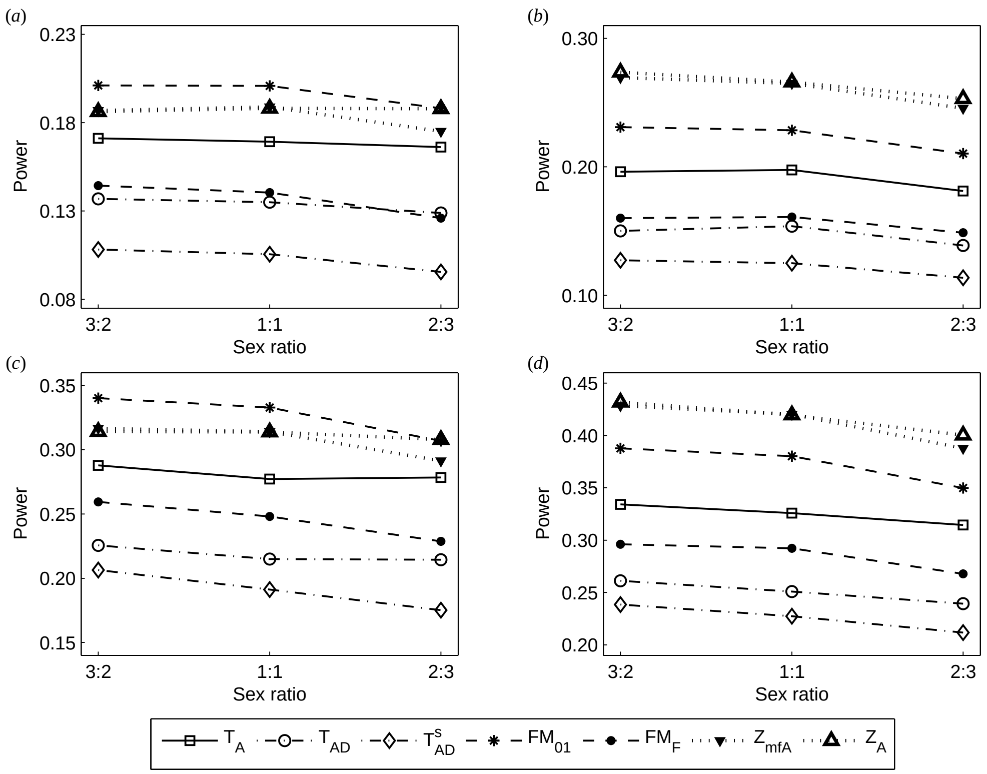

Supplementary Figure S50. Estimated powers of T_A, T_{AD}, T^s_{AD}, FM_{01}, FM_{F}, Z_{mfA} and Z_A against sex ratio ($r_f : r_m = 3 : 2$, $1 : 1$ and $2 : 3$) under the situation where $p_F = p_M = p$ but HWE does not hold in female offspring when there is neither XCI nor parent-of-origin effects. The simulation is based on 10 000 replicates with $N = 1000$, $\phi_{f0} = \phi_{m0} = 0.120$, $\phi_{f01} = \phi_{f10} = \phi_{m1} = 0.180$ and $\phi_{f2} = 0.240$. (a) $p = 0.2$, $\rho = -0.05$. (b) $p = 0.2$, $\rho = 0.05$. (c) $p = 0.3$, $\rho = -0.05$. (d) $p = 0.3$, $\rho = 0.05$.
